# Comparative effects of different exercise training modalities on exercise capacity, pulmonary function, and health-related quality of life in patients with interstitial lung disease: a systematic review and network meta-analysis

**DOI:** 10.3389/fmed.2026.1953668

**Published:** 2026-10-08

**Authors:** Yaozong Li, Jian Wang, Shujuan Zhang, Fan Wu, Wei Zhang

**Affiliations:** 1 Department of Pulmonary Diseases, Shuguang Hospital Affiliated to Shanghai University of Traditional Chinese Medicine, Shanghai, China; 2 School of Rehabilitation Science, Shanghai University of Traditional Chinese Medicine, Shanghai, China; 3 Department of Pulmonary Diseases, Shuguang Hospital, Shanghai Institute of Infectious Diseases and Biosecurity, Shanghai University of Traditional Chinese Medicine, Shanghai, China; 4 Wei Zhang Famous Traditional Chinese Medicine Expert Inheritance Studio, Shanghai Baoshan Hospital of Integrated Traditional Chinese and Western Medicine, Shanghai, China; 5 Institute of Infectious Diseases, Shanghai Institute of Traditional Chinese Medicine, Shanghai, China

**Keywords:** exercise training, health-related quality of life, interstitial lung disease, network meta-analysis, pulmonary rehabilitation, six-minute walk distance

## Abstract

**Introduction:**

Exercise training is central to pulmonary rehabilitation for interstitial lung disease (ILD), but the comparative effects of different exercise modalities remain uncertain. We compared their effects on exercise capacity, pulmonary function, and health-related quality of life.

**Methods:**

Six databases were searched from inception to June 11, 2026. Randomized controlled trials of exercise training in adults with ILD were included. Interventions were classified as aerobic training (AER), aerobic plus resistance training (AER_RES), respiratory-focused training comprising breathing exercises or inspiratory muscle training (BREATH_IMT), aerobic plus resistance plus breathing training (AER_RES_BREATH), mind–body exercise (MIND_BODY), or control (CON). Frequentist random-effects network meta-analyses evaluated changes in 6-min walk distance (6MWD), percentage predicted forced vital capacity (FVC% predicted), and St George's Respiratory Questionnaire (SGRQ) total score. Mean differences (MDs) with 95% confidence intervals (CIs) were estimated. RoB 2 and CINeMA were used to assess risk of bias and confidence in the evidence.

**Results:**

Twenty-six trials involving 1,409 participants were included. Compared with CON, all active modalities improved 6MWD, with MDs ranging from 29.46 m for AER (95% CI 11.82–47.10) to 57.12 m for AER_RES_BREATH (95% CI 41.76–72.48). AER_RES_BREATH produced greater estimated improvements than AER (MD 27.67 m, 95% CI 4.25–51.08) and AER_RES (MD 25.18 m, 95% CI 6.69–43.68), although these comparisons relied mainly on indirect evidence. Only AER_RES_BREATH showed a small improvement in FVC% predicted versus CON (MD 3.08 percentage points, 95% CI 0.05–6.12), with limited robustness and low confidence. AER_RES, AER_RES_BREATH, and MIND_BODY improved SGRQ total scores (MDs 10.08–15.20 points). Findings for 6MWD were the most robust across sensitivity analyses.

**Discussion:**

Exercise training improves functional exercise capacity and may improve health-related quality of life in adults with ILD. AER_RES_BREATH showed the strongest signal for 6MWD, but limited direct comparisons preclude its designation as the optimal intervention. Evidence for FVC% predicted was less robust and should not be interpreted as evidence of disease modification.

**Systematic Review Registration:**

https://www.crd.york.ac.uk/PROSPERO/view/CRD420261399024, identifier PROSPERO CRD420261399024.

## Introduction

Interstitial lung disease (ILD) comprises a heterogeneous group of disorders characterized primarily by inflammation and/or fibrosis of the lung parenchyma, including idiopathic pulmonary fibrosis, connective tissue disease-associated ILD, hypersensitivity pneumonitis, nonspecific interstitial pneumonia, and sarcoidosis (1). Evidence from the Global Burden of Disease studies indicates that the morbidity and mortality burden associated with ILD continues to increase worldwide (2, 3). Although the etiology and clinical course vary across ILD subtypes, patients commonly experience reduced lung compliance, impaired gas exchange, and exercise-induced hypoxemia, frequently accompanied by exertional dyspnea, fatigue, and reduced exercise capacity. These functional impairments may further contribute to decreased physical activity, limitations in activities of daily living, and deterioration in health-related quality of life (4, 5).

Current pharmacological treatments primarily aim to slow disease progression and target the underlying pathological processes, but their effects on established impairments in exercise function and limitations in physical activity remain relatively limited (1). Pulmonary rehabilitation seeks to improve patients’ overall functional status through exercise training, health education, symptom management, and behavioral interventions, with exercise training serving as its core component (6, 7). The American Thoracic Society clinical practice guideline has issued a strong recommendation for pulmonary rehabilitation in adults with ILD (6). Previous randomized controlled trials and systematic reviews have shown that pulmonary rehabilitation is generally safe and can improve functional exercise capacity, dyspnea, and health-related quality of life in patients with ILD in the short term (7–9).

Exercise interventions used in patients with ILD are diverse and include aerobic training, resistance training, respiratory muscle training, breathing exercises, mind–body exercise, and combinations of multiple training modalities. Different exercise modalities have distinct therapeutic emphases. Aerobic training primarily targets cardiorespiratory endurance, skeletal muscle oxygen utilization, and sustained exercise capacity, whereas resistance training focuses on improving peripheral muscle strength and endurance. Respiratory muscle training may alleviate dyspnea by increasing inspiratory muscle strength and endurance. Mind–body exercises, such as Tai Chi, Baduanjin, and yoga, integrate low- to moderate-intensity physical activity with breathing regulation (10, 11). Existing evidence suggests that different exercise programs may improve exercise capacity or quality of life; however, the magnitude and consistency of these effects vary across interventions (12). Therefore, although exercise training is recognized as an essential component of pulmonary rehabilitation for ILD, the optimal training modality and combination of exercise components remain uncertain.

Previous systematic reviews and conventional meta-analyses have generally grouped different exercise programs under the broad categories of “pulmonary rehabilitation” or “exercise training” and primarily compared their effects with those of usual care or no-exercise control. More recent studies have further examined program characteristics, such as intervention duration, level of supervision, and the use of high-intensity interval training. However, these studies have largely relied on pairwise meta-analyses or subgroup analyses and therefore could not simultaneously compare multiple interventions, including aerobic training, resistance training, respiratory muscle training, combined training, and mind–body exercise (12). In addition, published network meta-analyses of pulmonary rehabilitation have mainly focused on different delivery models, such as center-based, home-based, and telerehabilitation programs. These analyses have included patients with various chronic respiratory diseases and have not specifically evaluated the relative effects of different exercise components in patients with ILD (13). Given the limited number of direct head-to-head trials comparing different exercise modalities, conventional meta-analysis alone is insufficient to determine which training modality or combination of training components is most effective.

Provided that the assumptions of transitivity and consistency are reasonably satisfied, network meta-analysis can integrate direct and indirect evidence and simultaneously compare multiple interventions (14). Accordingly, we conducted this systematic review and network meta-analysis to compare the relative effects of different exercise training modalities on exercise capacity, pulmonary function, and health-related quality of life in adults with ILD, while interpreting intervention rankings in conjunction with the magnitude and certainty of the evidence.

## Methods

This systematic review was designed and reported in accordance with the PRISMA 2020 statement and the PRISMA extension for network meta-analyses (PRISMA-NMA) (15, 16).The review protocol was registered prospectively in PROSPERO (CRD420261399024).

### Search strategy

We systematically searched PubMed, Embase, Web of Science, Scopus, the Cochrane Library (CENTRAL), and PEDro from database inception. The search was updated before manuscript submission, with the final search conducted on June 11, 2026.The reference lists of the included studies and relevant systematic reviews were also screened to identify potentially eligible studies that may have been missed by the electronic searches. The search strategies combined controlled vocabulary terms and free-text terms related to interstitial lung disease, pulmonary rehabilitation, exercise training, and randomized controlled trials and were adapted according to the indexing systems and search requirements of each database. The complete search strategies are provided in Supplementary Appendix S1.

### Eligibility criteria

Studies were eligible if they met the following criteria: (1) participants were adults aged ≥18 years with interstitial lung disease (ILD) or related interstitial or fibrotic lung diseases; studies involving mixed populations were included only when data for the target population could be extracted separately; (2) the intervention consisted of structured, repeated active exercise training or pulmonary rehabilitation centered on active exercise training and could be classified into one of the prespecified network nodes according to its actual training components. Breathing exercises and inspiratory muscle training (IMT) were grouped within the same respiratory-focused training node because nodes were defined according to major training components rather than specific techniques. This pragmatic classification did not imply therapeutic equivalence and was retained to avoid excessively sparse nodes; (3) the study included at least one randomized between-group comparison across different intervention nodes, with comparators comprising usual care, health education, no exercise, a waiting-list control, sham training, or another eligible exercise intervention; (4) the study reported at least one of the following outcomes: 6-min walk distance (6MWD), percentage predicted forced vital capacity (FVC% predicted), or the total score of the standard St George's Respiratory Questionnaire (SGRQ), and provided sufficient data to extract directly or derive the group-level change scores and corresponding measures of dispersion at the end of the intervention. Absolute FVC and FVC% predicted, as well as the standard SGRQ total score and SGRQ-I, were treated as distinct outcomes and were not pooled; and (5) the study used a parallel-group, multi-arm, randomized pilot, or randomized feasibility design, and a full-text report in English containing sufficient methodological and outcome data was available. Complete preprints meeting all other eligibility criteria were also considered eligible.

We excluded non-randomized studies or studies in which randomization could not be confirmed; studies that compared interventions only within the same network node and included no randomized comparison across different nodes; studies in which passive neuromuscular electrical stimulation or whole-body vibration was the primary intervention without a structured active exercise component; studies that did not report any prespecified outcome or from which the required data could not be obtained; and reviews, study protocols, conference abstracts, and other non-original publications. Multi-arm trials were eligible if they included at least one comparison across different intervention nodes. Multiple arms classified within the same node were combined before inclusion in the network meta-analysis.

### Data extraction

Two reviewers independently extracted study characteristics, intervention details, and outcome data using a predesigned standardized form and independently classified each intervention into a network node according to prespecified criteria. Data requiring conversion from baseline and post-intervention values, standard errors, confidence intervals, medians and interquartile ranges, or graphical information were calculated using prespecified methods and cross-checked by the two reviewers. Disagreements were resolved through discussion, with arbitration by a third reviewer when necessary.

Mean change scores and their corresponding standard deviations at the end of the intervention were preferentially extracted. When the standard deviation of the change score had to be estimated, a correlation coefficient of (*r* = 0.50) was assumed in the primary analysis. Changes in SGRQ scores were reverse-coded before analysis so that positive values consistently indicated improvement. Multiple publications arising from the same trial were consolidated at the level of the independent trial, shared control groups were included only once, and multiple study arms assigned to the same network node within a trial were combined before analysis. Detailed data-conversion methods are provided in Supplementary Appendix S2 (17, 18).

### Risk-of-bias assessment

Two reviewers independently assessed the risk of bias in the included randomized controlled trials using the revised Cochrane risk-of-bias tool for randomized trials (RoB 2), with the effect of assignment to intervention as the target estimand (19). The assessment covered five domains: bias arising from the randomization process (D1), bias due to deviations from intended interventions (D2), bias due to missing outcome data (D3), bias in outcome measurement (D4), and bias in selection of the reported result (D5). Each domain was judged as having a low risk of bias, some concerns, or a high risk of bias, and an overall judgment was derived according to the RoB 2 algorithm.

Because blinding of participants and intervention providers is generally not feasible in exercise-based interventions, the absence of blinding alone was not considered sufficient to assign a high risk of bias. Instead, we further examined whether the lack of blinding resulted in deviations from the intended interventions or influenced outcome measurement. Disagreements were resolved through discussion, with arbitration by a third reviewer when necessary.

### Statistical analysis

Network meta-analyses were conducted within a frequentist framework using Stata/MP version 18.0 (20). The prespecified primary outcomes were 6-min walk distance (6MWD), percentage predicted forced vital capacity (FVC% predicted), and the total score of the standard St George's Respiratory Questionnaire (SGRQ), all of which were analyzed using change scores measured at the end of the intervention. Treatment effects were expressed as mean differences (MDs) on the original measurement scale with 95% confidence intervals (CIs): meters for 6MWD, percentage points for FVC% predicted, and points for the SGRQ total score. Changes in SGRQ scores were reverse-coded before analysis so that positive MDs consistently indicated a benefit of the intervention.

Between-study variance was estimated using restricted maximum likelihood, and a random-effects consistency model assuming a common heterogeneity variance was fitted within each outcome network. Correlations among effect estimates from multi-arm trials were accounted for in the model. Between-study heterogeneity was characterized using *τ* and *τ*^2^ and evaluated using Cochran's Q statistic; 95% prediction intervals were also calculated. Global and local inconsistency were evaluated using the design-by-treatment interaction model and node-splitting analyses, respectively.

The plausibility of the transitivity assumption was assessed separately for each outcome by comparing the distributions of prespecified potential effect modifiers across sets of direct evidence. These factors included ILD subtype, age, baseline outcome values and pulmonary function, intervention duration, delivery setting, and level of supervision; supplemental oxygen conditions and inspiratory muscle weakness were also examined when sufficient data were available. Transitivity was judged primarily on the basis of clinical comparability and the extent of overlap in the distributions of these variables, with additional consideration of the network structure, network meta-regression findings, and inconsistency assessments.

Intervention rankings were summarized using cumulative ranking probabilities, mean ranks, and the surface under the cumulative ranking curve (SUCRA), and were interpreted in conjunction with the corresponding relative effects and their uncertainty. In the league tables, the lower triangle presented random-effects network estimates, whereas the upper triangle presented direct-comparison results (21). When a direct comparison included at least two studies, a random-effects model with restricted maximum-likelihood estimation and Hartung–Knapp adjustment was used (22); when only one study was available, the estimate from that individual study was reported. Comparison-adjusted funnel plots were used to qualitatively explore small-study effects.

Sensitivity analyses included a fixed-effect MD model, a random-effects SMD model, leave-one-out analyses, and a restricted analysis including only trials that directly reported both mean change scores and their corresponding standard deviations. The restricted analysis was generally conducted using a random-effects consistency model with restricted maximum-likelihood estimation; however, a fixed-effect consistency model was used for the FVC% predicted network because the between-study variance could not be estimated. Exploratory subgroup analyses and network meta-regressions were further conducted according to intervention setting, intervention duration, disease type, and level of supervision. Confidence in the evidence was assessed using the CINeMA framework (23).

All statistical tests were two-sided. The primary analyses emphasized effect estimates and their 95% CIs rather than relying solely on *P* values. *P* values from subgroup analyses and network meta-regressions were not adjusted for multiple comparisons and were therefore interpreted as exploratory.

## Results

### Study selection

Two reviewers independently screened titles, abstracts, and full-text articles according to the prespecified eligibility criteria. Disagreements were resolved through discussion or, when necessary, by consultation with a third reviewer. Multiple publications, secondary analyses, and follow-up reports arising from the same trial were consolidated, and only the primary report was counted.

A total of 7,679 records were identified through searches of Embase, Scopus, the Cochrane Library, PubMed, Web of Science, and PEDro. After removal of 2,276 duplicates, 5,403 records underwent title and abstract screening, of which 5,353 were excluded. Full-text reports were sought for 50 articles; three could not be retrieved, leaving 47 reports for eligibility assessment. Following full-text review, 21 reports were excluded: three were non-randomized or had an unclear randomization status, four compared interventions classified within the same network node, six were secondary or companion reports, and eight did not meet the prespecified outcome or data requirements. Ultimately, 26 primary reports representing 26 independent randomized controlled trials were included in the review (Figure 1).

**Figure 1:**
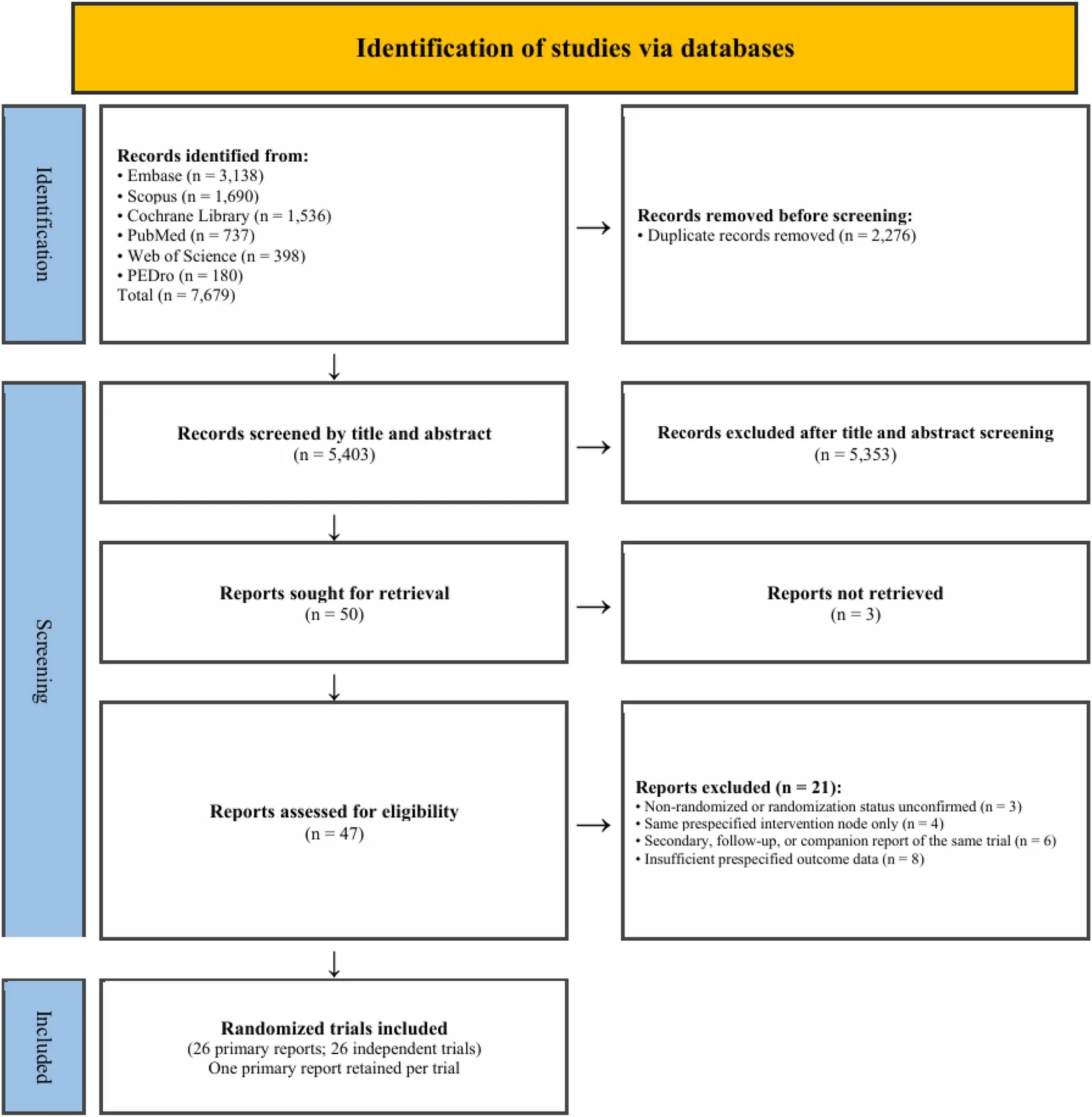
PRISMA 2020 flow diagram of the study selection process.

### Characteristics of the included studies

A total of 26 independent randomized controlled trials conducted in 12 countries were included, comprising 1,409 randomized participants. Sample sizes ranged from 16 to 210 participants per trial. Because outcome reporting and data completeness varied across studies, the outcome-specific analysis populations included 1,339 participants for 6MWD, 446 for FVC% predicted, and 431 for the SGRQ total score. The reported mean or median age across studies ranged from approximately 45.1 to 74.3 years.

The study populations included patients with idiopathic pulmonary fibrosis, mixed or fibrotic ILD, pulmonary sarcoidosis, systemic sclerosis-associated ILD, pneumoconiosis, asbestos- or dust-related lung disease, chronic hypersensitivity pneumonitis, and other connective tissue disease-associated ILDs. Based on the actual training components, active interventions were classified into five nodes: aerobic training (AER), combined aerobic and resistance training (AER_RES), respiratory-focused training comprising breathing exercises or inspiratory muscle training(BREATH_IMT), combined aerobic, resistance, and breathing training (AER_RES_BREATH), and mind–body exercise (MIND_BODY). The control node (CON) included usual care, health education, waiting-list control, no additional structured exercise, or sham training without an effective training load. When exercise advice or other background interventions were delivered equally across randomized groups, they were treated as co-interventions.

A separate pulmonary rehabilitation node was not created; instead, interventions were classified according to their explicitly reported training components. No eligible trial evaluated balance training as a standalone principal intervention; therefore, a separate balance-training node was not created. No eligible resistance-training-only node was available for the primary analysis. Intervention duration ranged from 3 to 52 weeks. Detailed study characteristics are presented in Table 1, and the node definitions and outcome-specific network structures are provided in Supplementary Appendix S2 and Supplementary Table S1.

**Table 1:** Characteristics of the 26 included independent randomized trials.

Study	Country	Exercise	Sample size, randomized/analyzed; sex (M/F)	ILD type/severity	Mean age, years	Interventions and intensity	Intervention duration	Outcomes
Holland 2008 (24)	Australia	AER_RES vs. CON	57 (M/F NR)	Mixed ILD; IPF *n* = 34; stable symptomatic disease; FVC 75 ± 25% (control) and 77 ± 24% (exercise) predicted	Control 67 ± 13; exercise 70 ± 8	Exercise: supervised cycling and walking for 30 min (walking at 80% of baseline 6MWT speed) plus upper-limb endurance and lower-limb functional strength, twice weekly; home exercise to reach 5 sessions/week. Control: weekly telephone support/usual care.	8 weeks	6MWD; CPET/peak VO₂; FVC; DLCO; CRQ; SF-36; mMRC
Nishiyama 2008 (25)	Japan	AER_RES vs. CON	30 randomized; 28 analyzed (21/7)	IPF; FVC 68.7 ± 19.5% (control) and 66.1 ± 13.2% (rehabilitation) predicted	Control 64.5 ± 9.1; rehabilitation 68.1 ± 8.9	Rehabilitation: outpatient treadmill/cycle training at 80% of baseline maximal walking speed/workload plus upper- and lower-limb resistance using elastic bands (∼20 min), twice weekly; education also provided. Control: usual care.	10 weeks	6MWD; FVC; FEV₁; TLC; DLCO; PaO₂/PaCO₂; MIP/MEP; BDI; SGRQ
Dale 2014 (26)	Australia	AER vs. CON	35 (35/0); 33 completed	Asbestos-related and other dust-related respiratory diseases; 69% asbestos-related pleural disease; baseline FVC 86% predicted in both groups	Exercise 70 ± 7; control 72 ± 6	Exercise: supervised walking and cycling, 3 sessions/week, initially prescribed at ∼80% of baseline capacity and progressed as tolerated; the protocol explicitly did not include strength training. Control: usual care.	8 weeks	6MWD; CRQ; SGRQ; muscle strength; physical activity
Jackson 2014 (27)	USA	AER_RES vs. CON	21 (M/F NR); rehabilitation/control 11/10	IPF; mild-to-moderate restriction (mean FVC 61 ± 12% predicted) and moderate-to-severe diffusion impairment (mean DLCO 44 ± 10% predicted)	Rehabilitation 71 ± 6; control 66 ± 7	Rehabilitation: 24 supervised 2-h sessions (twice weekly) with treadmill/cycle aerobic exercise at 60%–80% of age-predicted maximal heart rate, flexibility and Theraband strength training; home strength sessions were prescribed. Control: observation/usual care.	12 weeks	6MWD; constant-load cycle endurance and VO₂; MIP/MEP; muscle strength; oxidative-stress/biomarker measures; SGRQ
Vainshelboim 2014 (28)	Israel	AER_RES_BREATH vs. CON	34 randomized; 32 analyzed (21/11)	IPF; FVC 70.1 ± 17.4% (control) and 66.1 ± 14.8% (exercise) predicted	Control 66 ± 9; exercise 68.8 ± 6.0	Exercise: supervised 60-min sessions twice weekly, including interval treadmill/cycle/step aerobic training (60%–70% peak work rate; walking 80%–90% baseline speed), dumbbell resistance, deep-breathing exercises and flexibility. Control: usual care.	12 weeks	6MWD; CPET; 30-s chair stand; pulmonary function; mMRC; SGRQ
Karadalli 2016 (29)	Türkiye	BREATH_IMT vs. CON	34 assigned; 30 completed (11/19)	Pulmonary sarcoidosis; radiographic stage I *n* = 9 and stage II *n* = 21	IMT 45.1 ± 8.1; sham 47.5 ± 12.9	IMT: threshold loading at 40% PImax, recalibrated weekly; sham: fixed 5% PImax. Both groups trained 30 min/day, 7 days/week (6 home and 1 supervised session/week), progressing from 10 to 15 to 25–30 breaths/load.	6 weeks	PImax/PEmax; 6MWD; incremental shuttle walk test; CPET; peripheral muscle strength; pulmonary function/DLCO; dyspnea; fatigue; depression; SGRQ
Dowman 2017 (30)	Australia	AER_RES vs. CON	142 (M/F NR)	Mixed ILD: IPF *n* = 61, asbestosis *n* = 22, CTD-ILD *n* = 23, other ILD *n* = 36; subgroup baseline FVC approximately 71%–85% predicted	IPF usual care/exercise 73 ± 9/70 ± 10; asbestosis 72 ± 9/72 ± 7; CTD-ILD 65 ± 11/63 ± 10; other ILD NR	Exercise: supervised walking and cycling (30 min; walking at 80% peak 6MWT speed, cycling at 70% estimated maximum) plus upper-/lower-limb resistance at 10–12 RM, twice weekly, with home exercise. Control: usual care and weekly support calls.	8 weeks	6MWD; knee-extensor strength; CRDQ; SGRQ-I; UCSD-SOBQ/mMRC dyspnea; HADS; pulmonary function
Naz 2018 (31)	Türkiye	AER_RES_BREATH vs. CON	18 (6/12); exercise/control 9/9	Stable pulmonary sarcoidosis; radiographic stages III–IV	Exercise median 59 (IQR 52–64); control 51 (45–57)	Exercise: supervised twice weekly; treadmill walking (80% peak 6MWT speed) and cycle ergometry (70% estimated maximum work rate), 15 min each, plus progressive upper-/lower-limb resistance, breathing and stretching; Borg dyspnea/exertion target 4–6. Control: usual care.	12 weeks	6MWD; respiratory/peripheral muscle strength; pulmonary function/DLCO; blood gases; mMRC; FSS; SGRQ; SF-36; HADS
Perez-Bogerd 2018 (32)	Belgium	AER_RES vs. CON	60 (37/23); rehabilitation/control 30/30	Chronic mixed ILD; IPF *n* = 14 (23%); other IIP, chronic HP, CTD-ILD and unclassifiable ILD included	Control 64 ± 8; rehabilitation 64 ± 13	Rehabilitation: multidisciplinary outpatient PR (60 sessions), 3/week for 3 months then 2/week for 3 months; progressive aerobic and resistance training. Control: maximal medical/usual care.	26 weeks	6MWD; CPET/maximal work rate; SVC/FVC; DLCO; quadriceps/handgrip force; SGRQ; CRQ; objectively measured physical activity
El-Komy 2019 (33)	Egypt	AER_RES_BREATH vs. CON	50 (18/32); PR/control 25/25	Stable ILD: IPF *n* = 12, CTD-ILD *n* = 18, hypersensitivity pneumonitis *n* = 20; baseline FVC ranges 54%–60% and 55%–58% predicted in PR/control groups	PR 47.3 ± 12.7; control 48.8 ± 10.14	PR plus medication: 2 supervised and 3 home sessions/week; interval cycle/arm-wheel endurance (initially 80%–100% maximum HR; progressed as tolerated), upper-limb resistance at 50%–85% 1RM, pursed-lip/diaphragmatic breathing and incentive spirometry, plus education. Control: medication alone.	8 weeks	6MWD; spirometry/MVV; arterial blood gases; mMRC; SF-36
Yuen 2019 (34)	USA	AER vs. CON	20 (13/7); exergame/control 10/10	IPF; baseline FVC 2.0 ± 0.5 L (exergame) and 3.1 ± 0.7 L (control)	Exergame 67.4 ± 7.4; video-game control 72.2 ± 8.4	Exergame: home Wii Fit whole-body activity at least 3 days/week, with a target of ≥150 min/week. Control: sedentary Wii video games; both groups received usual exercise advice.	12 weeks	Adherence/feasibility; 6MWD; exercise dyspnea; SGRQ; pulmonary function
Jarosch 2020 (35)	Germany	AER_RES_BREATH vs. CON	54 randomized; 51 analyzed (38/13)	IPF; FVC ≥50% predicted; stable treatment	Usual care 65 ± 10; PR 68 ± 9	Comprehensive inpatient PR: cycle endurance/interval training at 60%/100% peak work rate and resistance at 15–20 RM, 5–6 days/week (15–18 sessions), plus breathing therapy, education, psychosocial and medical care. Control: usual care.	3 weeks	6MWD; CRQ; SF-36; HADS; pulmonary function; physical activity
Cerdan-de-las-Heras 2021 (36)	Denmark	AER_RES vs. CON	29 (21/8); tele-rehabilitation/control 15/14	Stable IPF; FVC 76.73 ± 16.4% (tele-rehabilitation) and 90.8 ± 16.5% (control) predicted; DLCO 46.46 ± 11.0% and 55 ± 14.0% predicted	Tele-rehabilitation 70.1 ± 8.8; control 72.4 ± 7.6	VAPA tele-rehabilitation plus usual care: tailored 10–20-min home workouts using step, elastics and weights, 3–5 times/week, with video consultations, e-learning and monitoring. Control: usual care without rehabilitation.	12 weeks	6MWD; 7-day pedometry; FVC; DLCO; SGRQ-I; K-BILD; GAD-7; adherence/satisfaction; adverse events
Shen 2021 (37)	China	BREATH_IMT vs. CON	82 (77/5); exercise/control 39/43	Stable IPF; baseline FVC 2.50 ± 0.59 L (exercise) and 2.45 ± 0.68 L (control); DLCO 66.83 ± 19.94% and 59.11 ± 21.49%	Exercise 65.31 ± 6.11; control 64.95 ± 7.97	Liuzijue health-preserving respiratory physical fitness program plus usual care: 3 sets of breathing movements, 3 times/day (4–6 min each), with twice-weekly supervised training before trial initiation and ongoing clinic management. Control: usual medical care.	52 weeks	FVC; chest-x-ray lung volume; FEV₁; DLCO; 6MWD; SGRQ; acute exacerbation; survival/safety
Wang 2021 (38)	China	AER_RES_BREATH vs. CON	64 enrolled; 60 analyzed (29/31)	Idiopathic interstitial pneumonia/ILD; 58 IPF, 3 NSIP, 1 asbestosis and 2 rheumatoid arthritis-associated ILD among 64 enrolled; analyzed groups had mild restriction	Control 56.7 ± 6.3; home PR 55.9 ± 7.2	Home PR plus medication: walking/brisk walking/jogging/stairs/cycling and local muscle training 3–5 times/week, respiratory muscle/breathing exercises, oxygen when indicated, education and psychological/behavioral support. Control: conventional drug treatment.	12 weeks	6MWD; FVC; FEV₁; DLCO; mMRC; SGRQ; 12-month durability
Zhou 2021 (39)	China	MIND_BODY vs. AER vs. CON	96 randomized; 94 in baseline table (54/40); 89 completed	Stable IPF	Control 67 ± 10; cycle exercise 64 ± 9; pulmonary Daoyin 66 ± 11	Pulmonary Daoyin: 1 h/day (may be split), 5 days/week for 1 supervised month then home practice twice daily during month 2. Active comparator: stationary cycle ergometer 30 min/day, 5 days/week. Control: usual activities/no extra exercise.	8 weeks	6MWD; SGRQ-I; mMRC; FVC; DLCO; adverse events; 6-month follow-up
Cerdan-de-las-Heras 2022 (40)	Denmark	AER_RES vs. CON	30 (19/11); tele-rehabilitation/control 15/15	Stable sarcoidosis; FVC 93.13 ± 19.38% (tele-rehabilitation) and 84.73 ± 17.93% (control) predicted; DLCO 65.87 ± 15.91% and 64.30 ± 17.18% predicted	Tele-rehabilitation 56.1 ± 14.4; control 51.6 ± 12.7	VAPA tele-rehabilitation: individualized program combining aerobic and strength exercises (≥60 min/week), video consultations, e-learning, monitoring and usual care. Control: usual care without systematic training.	12 weeks	6MWD; dyspnea/Borg; maximal voluntary contraction; pedometry; FVC/FEV₁/DLCO; SGRQ-I; K-BILD; GAD-7; adherence/satisfaction; safety
Kataoka 2023 (41)	Japan	AER_RES vs. CON	88 (69/19); PR/control 45/43	Stable IPF receiving nintedanib; baseline FVC 74.3 ± 14.7% (PR) and 74.0 ± 13.8% (control) predicted	PR 71.2 ± 4.9; control 70.4 ± 5.5	Both groups continued nintedanib. PR: 12-week induction with supervised cycle/walking twice weekly (80% baseline capacity), progressive upper-/lower-limb resistance and home exercise, followed by 40-week maintenance with home training and outpatient supervision ≥ once every 4 weeks. Control: usual care without PR.	12 weeks (induction endpoint)	6MWD; cycle endurance time; SGRQ; CAT; dyspnea; daily steps; FVC; DLCO; adverse events
Zaki 2023 (42)	India	AER_RES_BREATH vs. AER_RES	51 (27/24); PR + IMT/PR 26/25	Mixed ILD with inspiratory muscle weakness; specific ILD type/severity distribution NR	PR + IMT 53.35 ± 13.61; PR 53.12 ± 13.33	Both groups: supervised multidisciplinary PR 3/week with cycle and treadmill (15 min each; 70% maximum work rate and 80% peak 6MWT speed), upper-/lower-limb resistance at 10–12 RM, progressed to RPE 12–14. PR + IMT additionally received 21-min threshold IMT 3/week, starting at 30% PImax and increasing 10% every 2 weeks to 60%.	8 weeks	PImax; 6MWD; SGRQ; mMRC; FEV₁; FVC; TLC; RV; DLCO
Aktan 2024 (43)	Türkiye	BREATH_IMT vs. CON	28 (19/9); IMT/sham 14/14	Clinically stable IPF; overall FVC 78.6 ± 14.6% predicted	IMT 66.5 ± 5.5; sham 68.9 ± 7.0	Telerehabilitation-assisted home IMT: 50% baseline MIP, progressed to Borg 4–6, two sets of 30 repetitions twice daily, 7 days/week; sham IMT without resistance. Weekly video supervision was provided.	8 weeks	MIP; FVC; FEV₁; DLCO; 6MWD; mMRC
Atmaca 2025 (44)	Türkiye	MIND_BODY vs. CON	28 (22/6); exercise/control 14/14	IPF; baseline FVC 60.14 ± 10.65% (exercise) and 63.93 ± 15.63% (control) predicted	Exercise 61.64 ± 8.01; control 59.00 ± 7.32	Baduanjin: supervised online sessions 3/week plus 1 unsupervised home session/week (24 supervised sessions); intensity individualized within tolerance. Control: no exercise training/usual care.	8 weeks	6MWD; FVC/FEV₁/PEF; MIP/MEP; SGRQ
Kadura 2025 (45)	USA	MIND_BODY vs. CON	63 randomized; 60 completed/analyzed (41/19)	IPF; analyzed sample FVC 79.6 ± 20.28% (yoga) and 70.0 ± 21.84% (control) predicted	Yoga 72.5 ± 8.97; control 74.3 ± 7.41 (analyzed sample)	Modified chair yoga with seated postures, breathing and meditation: virtual 60-min group sessions twice weekly, plus optional video-guided practice. Control: standard care/usual activities.	12 weeks	L-IPF symptoms; R-Scale-PF; K-BILD; EQ-5D-5L; HADS; PROMIS sleep measures; Epworth Sleepiness Scale; FVC; DLCO; 6MWD; safety/adherence
Sarıtaş Arslan 2026 (46)	Türkiye	AER (AVG) vs. AER (TAE) vs. CON; the two AER arms were pooled within study for the NMA	45 (16/29); AVG/TAE/control 15/15/15	Mixed ILD: IPF *n* = 12, systemic sclerosis-associated ILD *n* = 16, chronic HP *n* = 6 and chronic sarcoidosis *n* = 11; baseline FVC 83.36 ± 23.98% (AVG), 77.83 ± 19.74% (TAE) and 76.34 ± 15.39% (control) predicted	AVG 57.80 ± 7.57; TAE 57.33 ± 7.35; control 58.33 ± 6.41	All groups received lifestyle advice, breathing exercises twice daily, and walking for 30 min twice weekly at 80% of mean baseline 6MWT speed. TAE received supervised hospital cycling for 30 min twice weekly at 60%–80% of maximum heart rate and Borg dyspnea 4–6. AVG received the same cycling program plus 30 min of Wii Fit exergaming after a 10–15-min rest. The control group received no additional supervised cycling or exergaming intervention.	8 weeks	6MWD; ISWT; ESWT; FVC; DLCO; MIP/MEP; quadriceps strength; mMRC; SGRQ; LC-ADL; FSS; IPAQ; HADS; LCQ; attendance/satisfaction/enjoyment; adverse events
Vicenzi 2026 (47)	Italy	BREATH_IMT vs. CON	24 (4/20); IMT/sham 12/12	Systemic sclerosis-associated ILD confirmed by HRCT; stable medical therapy; severe ILD excluded (DLCO <45% and/or FVC <70%); baseline FVC 104 ± 15% (IMT) and 96 ± 16% (sham) predicted	IMT 61 ± 8; sham 63 ± 9	IMT: Airofit PRO at an individualized load of 60% MIP; sham: identical device/schedule at 5% MIP. Both groups completed 30 inspirations/session, twice daily, 7 days/week, for 5 weeks; adherence was monitored by weekly video calls and the device dashboard.	5 weeks	MIP; FVC; FEV₁; peak expiratory/inspiratory flow; 6MWD; ventilation, breathing frequency and tidal volume; VO₂/VCO₂; heart rate; dyspnea/fatigue RPE
Buschulte 2026 (48)	Germany	MIND_BODY vs. CON	16 (11/5); yoga/control 9/7; 12 completers	Fibrosing ILD (IPF, fibrosing HP, SSc-ILD, iNSIP and unclassifiable ILD); FVC 67.8 ± 19.4% (yoga) and 78.8 ± 19.1% (control) predicted	Yoga 65.1 ± 8.2; control 71.3 ± 9.3	Adapted yoga focusing on physical postures, breathing/respiratory-muscle work and mental well-being: supervised 60-min group sessions twice weekly; home practice encouraged. Control: surveillance/usual care.	12 weeks	K-BILD; HADS; LCQ; dyspnea VAS; 6MWD; FVC; DLCO; inflammatory/cardiac biomarkers; qualitative feasibility/safety
Zou 2026 (49)	China	AER_RES_BREATH vs. CON	210 randomized; 198 analyzed (198/0)	Stable pneumoconiosis, stages I–III	Rehabilitation 63.24 ± 9.73; control 62.47 ± 9.08	Individualized comprehensive PR plus routine treatment: respiratory-muscle training, dumbbell/quadriceps resistance, cycling, treadmill and walking generally 4 days/week with staged progression, plus education, nutritional and psychological intervention. Control: routine treatment.	52 weeks	FVC; FEV₁; FEV₁/FVC; PEF/MEF; MIP/MEP; 6MWD; CAT; mMRC; Borg; HADS; MNA-SF

Extraction was performed at the independent-trial level. Interventions were classified by their explicitly prescribed active training components rather than by the label “pulmonary rehabilitation,” delivery setting, or supervision format. The final taxonomy comprised AER, AER_RES, BREATH_IMT, AER_RES_BREATH, MIND_BODY, and CON; no eligible standalone RES arm remained. Trials in which passive NMES or whole-body vibration was the defining intervention without a structured active-exercise component were excluded from the final analysis. Shared controls were retained once, same-node arms within a trial were pooled before NMA, and companion publications were linked to one independent trial. NR indicates information not reported or not confirmable; no unreported study-characteristic values were imputed. Detailed source pages and transformation records are provided in Supplementary Appendix S2 and the final extraction workbook. Outcomes listed in this table are those reported in the original trials. Only 6MWD, FVC% predicted, and standard SGRQ Total contributed to the prespecified network meta-analyses; absolute FVC in litres, SGRQ-I, and SGRQ domain scores were not pooled.

AER, aerobic training; AER_RES, aerobic plus resistance training; AER_RES_BREATH, aerobic plus resistance plus breathing/inspiratory muscle training; BREATH_IMT, respiratory-focused training comprising breathing exercises or inspiratory muscle training; CON, control/usual care/sham; MIND_BODY, mind-body exercise; 1RM, one-repetition maximum; 6MWD/6MWT, 6-minute walk distance/test; AVG, active video game; BDI, Baseline Dyspnea Index; CAT, COPD Assessment Test; CPET, cardiopulmonary exercise test; CRQ/CRDQ, Chronic Respiratory (Disease) Questionnaire; CTD-ILD, connective tissue disease-associated interstitial lung disease; DLCO, diffusing capacity of the lung for carbon monoxide; ESWT, endurance shuttle walk test; FAS/FSS, Fatigue Assessment/Severity Scale; FEV₁, forced expiratory volume in 1 s; FVC, forced vital capacity; GAD-7, Generalized Anxiety Disorder-7; HADS, Hospital Anxiety and Depression Scale; HP, hypersensitivity pneumonitis; ILD, interstitial lung disease; IMT, inspiratory muscle training; IPAQ, International Physical Activity Questionnaire; IPF, idiopathic pulmonary fibrosis; ISWT, incremental shuttle walk test; K-BILD, King's Brief Interstitial Lung Disease questionnaire; LC-ADL, London Chest Activities of Daily Living Scale; LCQ, Leicester Cough Questionnaire; MEP, maximal expiratory pressure; MIP, maximal inspiratory pressure; mMRC, modified Medical Research Council; MNA-SF, Mini Nutritional Assessment-Short Form; NR, not reported/confirmable; NSIP, nonspecific interstitial pneumonia; PImax/PEmax, maximal inspiratory/expiratory pressure; PR, pulmonary rehabilitation; RM, repetition maximum; SF-36, 36-Item Short Form Health Survey; SGRQ, St George's Respiratory Questionnaire; SGRQ-I, IPF-specific SGRQ; SSc-ILD, systemic sclerosis-associated ILD; TAE, traditional aerobic exercise; TLC, total lung capacity; UCSD-SOBQ, University of California San Diego Shortness of Breath Questionnaire; VAPA, Virtual Autonomous Physiotherapist Agent.

### Risk of bias

For the randomization process domain, 16 trials were judged to be at low risk of bias and 10 raised some concerns, primarily because of insufficient reporting of random sequence generation or allocation concealment. All 26 trials were judged to be at low risk of bias for deviations from the intended interventions.

For missing outcome data, 19 trials were judged to be at low risk of bias, four raised some concerns, and three were judged to be at high risk because of substantial missing data, imbalanced attrition between groups, or inadequate methods for handling missing data. For outcome measurement, 23 trials were judged to be at low risk of bias and three raised some concerns, mainly because the absence of blinded assessment may have influenced patient-reported outcomes or exercise performance measures. All 26 trials were judged to be at low risk of bias for selection of the reported result.

Overall, 10 trials (38.5%) were judged to be at low risk of bias, 13 (50.0%) raised some concerns, and three (11.5%) were judged to be at high risk of bias (Figure 2).

**Figure 2:**
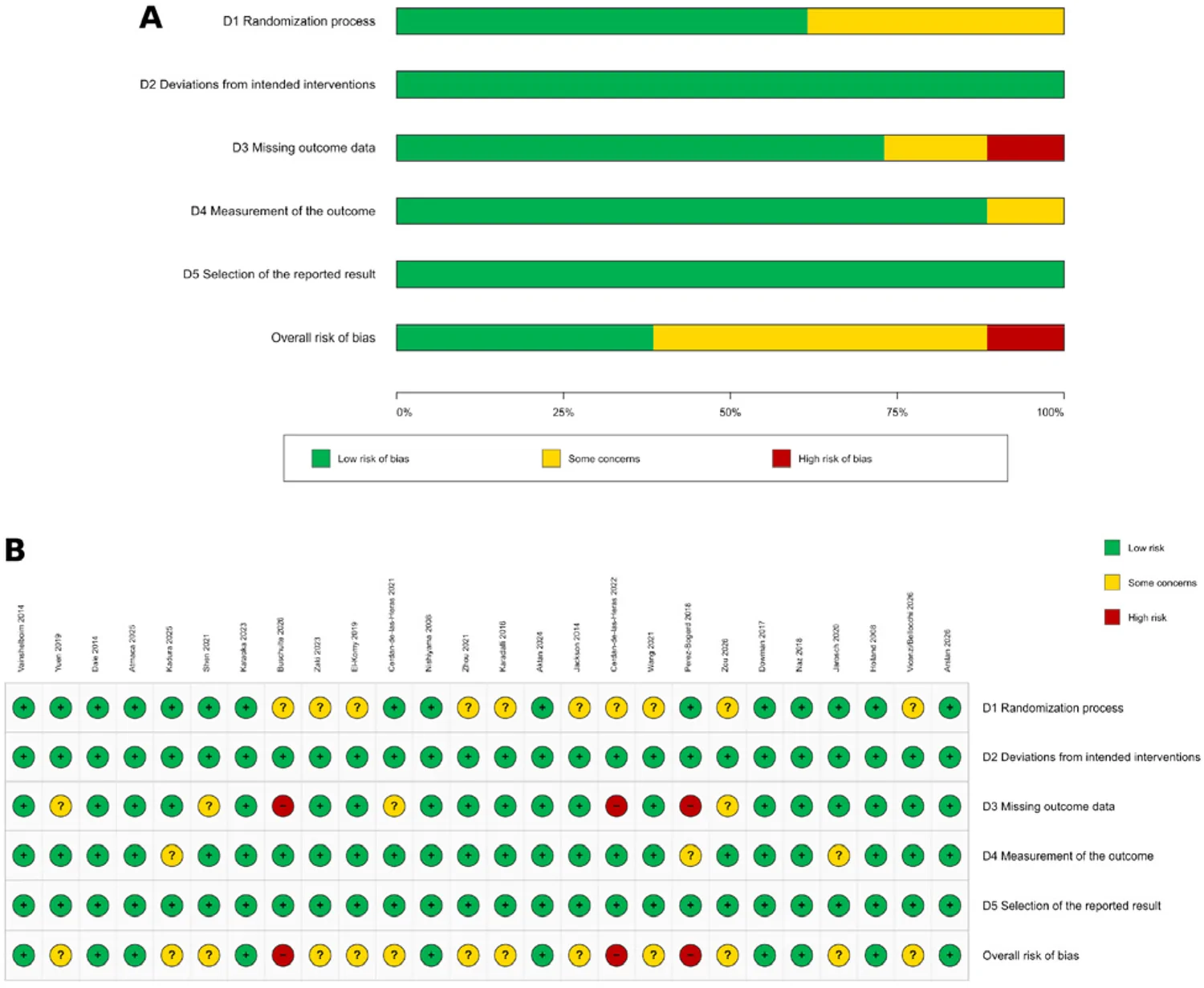
Risk-of-bias assessment of the included randomized controlled trials using the revised cochrane risk-of-bias tool for randomized trials (roB 2). **(A)** Distribution of risk-of-bias judgments across individual domains and overall risk of bias; **(B)** traffic-light plot showing the domain-level and overall judgments for each included trial.

### Network meta-analysis results

All three outcomes formed connected networks, with usual care or a non-exercise control (CON) serving as the principal common comparator. The FVC analysis included only percentage predicted FVC and did not pool these data with absolute FVC values reported in liters. Similarly, the SGRQ analysis included only the total score of the standard SGRQ and did not combine it with SGRQ-I scores or domain-specific scores.

The 6MWD network included 26 studies, 53 analytical arms, and 1,339 participants. The FVC% predicted network included 11 studies, 22 analytical arms, and 446 participants, whereas the SGRQ total score network included 11 studies, 22 analytical arms, and 431 participants. Each network comprised six nodes: CON, AER, AER_RES, BREATH_IMT, AER_RES_BREATH, and MIND_BODY. Most of the evidence was derived from direct comparisons between active interventions and CON, whereas head-to-head comparisons among active interventions were limited. Consequently, all three networks showed an approximately star-shaped structure centered on CON (Figure 3; Supplementary Table S1).

**Figure 3:**
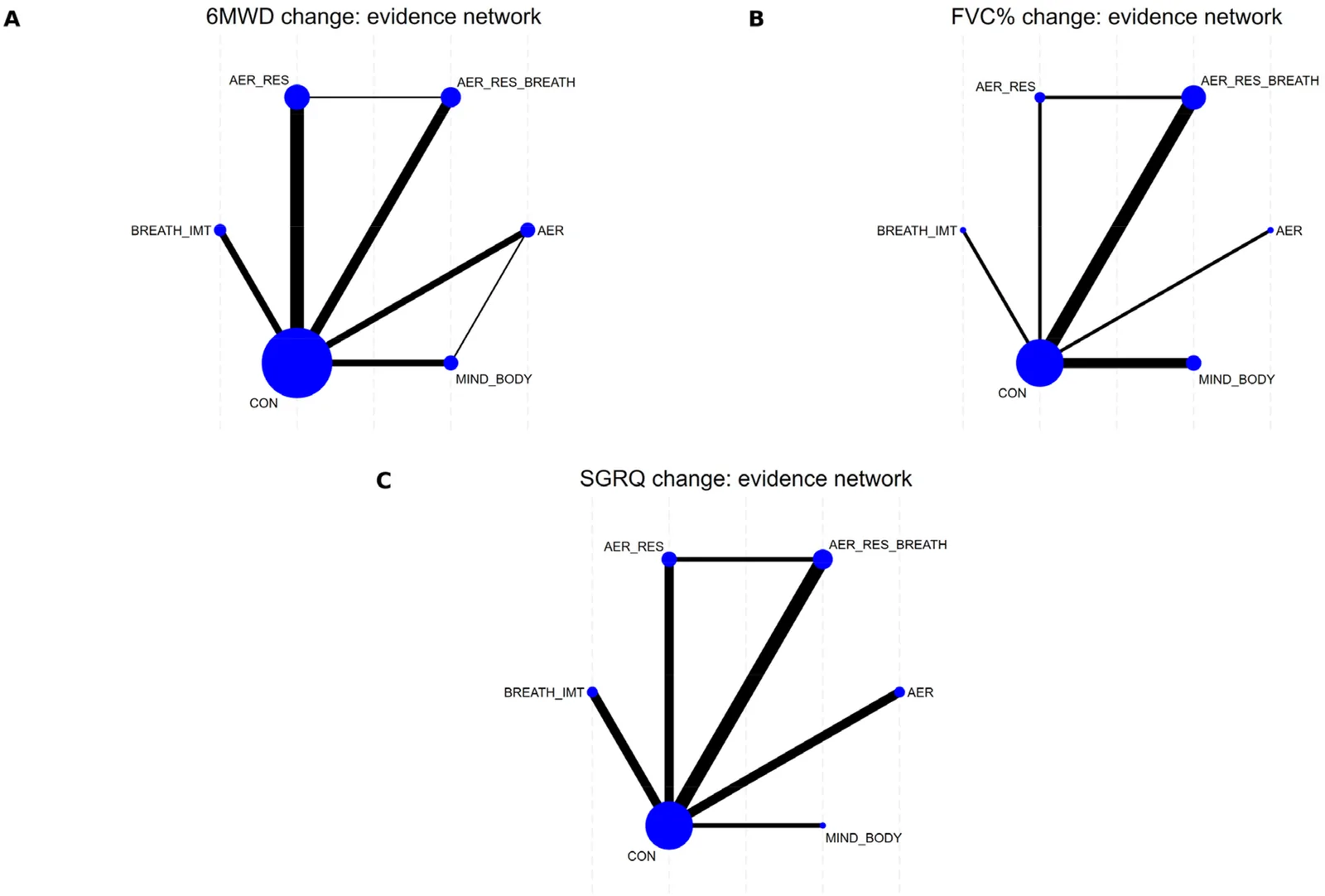
Evidence network plots for the three primary outcomes. **(A)** Six-minute walk distance; **(B)** percent-predicted forced vital capacity; and **(C)** St George’s Respiratory Questionnaire total score. The size of each node is proportional to the number of participants receiving the corresponding intervention, and the thickness of each connecting line is proportional to the amount of direct comparative evidence.

### Assessment of transitivity

The distributions of age, baseline outcome values, and selected pulmonary function measures showed some overlap across sets of direct evidence; however, the distributions of disease type, baseline severity, intervention duration, delivery setting, and level of supervision were not fully balanced. Trials involving AER_RES_BREATH generally enrolled younger participants with relatively poorer baseline pulmonary function and were predominantly conducted in center- or hospital-based settings with a high level of direct supervision. The only trial directly comparing AER_RES_BREATH with AER_RES specifically enrolled patients with inspiratory muscle weakness. The FVC% predicted and SGRQ total-score networks were relatively sparse, with some direct comparisons supported by only a single study, and reporting of supplemental oxygen use and baseline inspiratory muscle function was incomplete. Overall, the transitivity assumption was considered reasonably plausible, although some uncertainty remained, particularly for comparisons among active interventions that relied predominantly on indirect evidence (Supplementary Table S1B).

### Six-minute walk distance

The random-effects network meta-analysis showed that, compared with CON, AER (MD = 29.46 m, 95% CI 11.82 to 47.10), AER_RES (MD = 31.94 m, 95% CI 17.61 to 46.27), BREATH_IMT (MD = 48.59 m, 95% CI 20.18 to 77.01), AER_RES_BREATH (MD = 57.12 m, 95% CI 41.76 to 72.48), and MIND_BODY (MD = 43.48 m, 95% CI 20.91 to 66.05) were all associated with greater improvements in 6MWD (Figure 4B; Supplementary Figure S1A).

**Figure 4:**
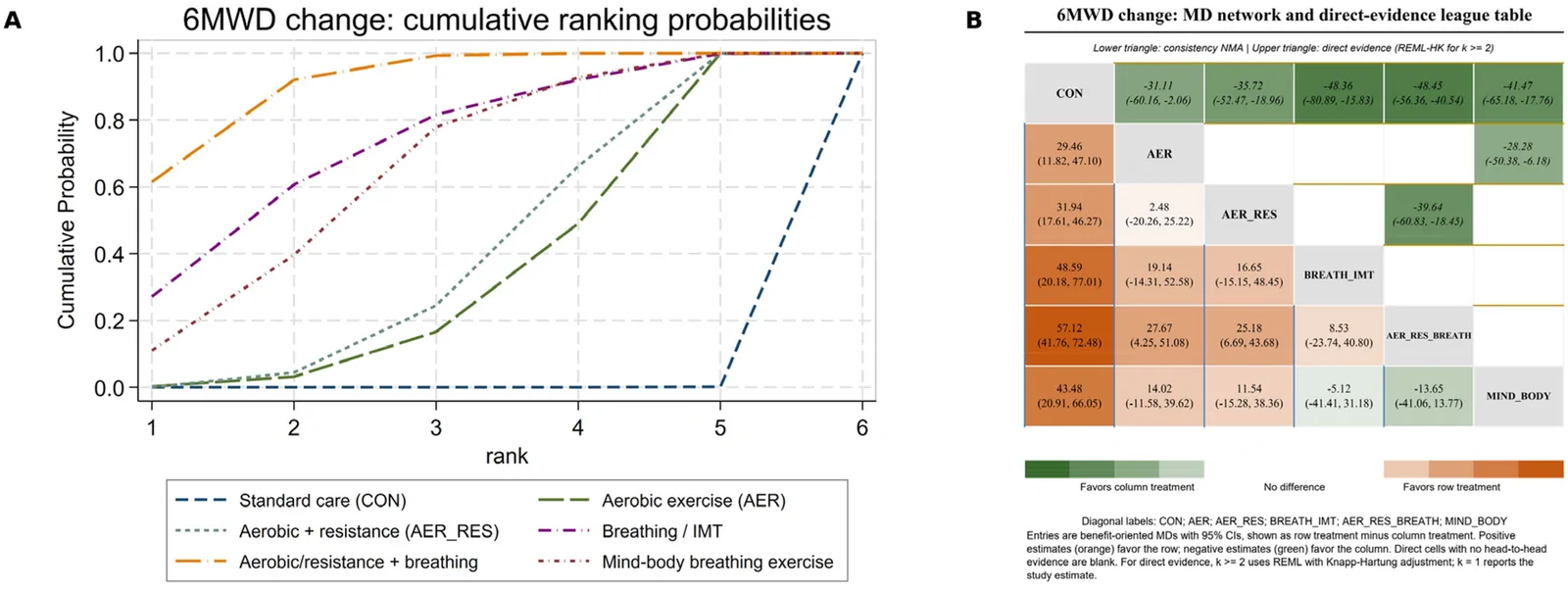
Main network meta-analysis results for the six-minute walk distance. **(A)** Cumulative ranking probability plot; and **(B)** league table of pairwise treatment comparisons. The lower triangle presents estimates from the random-effects network meta-analysis, whereas the upper triangle presents estimates from direct comparisons. Treatment effects are reported as mean differences with 95% confidence intervals. 6MWD, six-minute walk distance; AER, aerobic training; AER_RES, combined aerobic and resistance training; BREATH_IMT, respiratory-focused training comprising breathing exercises or inspiratory muscle training; AER_RES_BREATH, combined aerobic, resistance, and breathing or inspiratory muscle training; MIND_BODY, mind–body exercise; CON, control; MD, mean difference; CI, confidence interval.

Network estimates for comparisons among active interventions indicated that AER_RES_BREATH may produce greater improvements in 6MWD than AER (MD = 27.67 m, 95% CI 4.25 to 51.08) and AER_RES (MD = 25.18 m, 95% CI 6.69 to 43.68). No clear differences were observed in the remaining comparisons among active interventions. Because direct head-to-head evidence between active interventions was limited, these estimates were derived predominantly from indirect evidence and should therefore be interpreted with caution (Figure 4B).

AER_RES_BREATH ranked first, with a SUCRA value of 90.57% and a mean rank of 1.47. BREATH_IMT and MIND_BODY ranked second and third, with SUCRA values of 72.28% and 64.27%, respectively. These rankings should be interpreted alongside the corresponding relative effect estimates, confidence intervals, amount of direct evidence, and certainty of the evidence (Figure 4A; Supplementary Table S6).

### Percentage predicted forced vital capacity

Compared with CON, the random-effects estimate for AER_RES_BREATH was 3.08 percentage points (95% CI 0.05 to 6.12). The confidence intervals for AER (MD = −0.40 percentage points, 95% CI −5.47 to 4.67), AER_RES (MD = 3.12 percentage points, 95% CI −1.12 to 7.36), BREATH_IMT (MD = −1.00 percentage points, 95% CI −13.11 to 11.11), and MIND_BODY (MD = −0.69 percentage points, 95% CI −6.80 to 5.42) all included the null value (Figure 5B; Supplementary Figure S1B).

**Figure 5:**
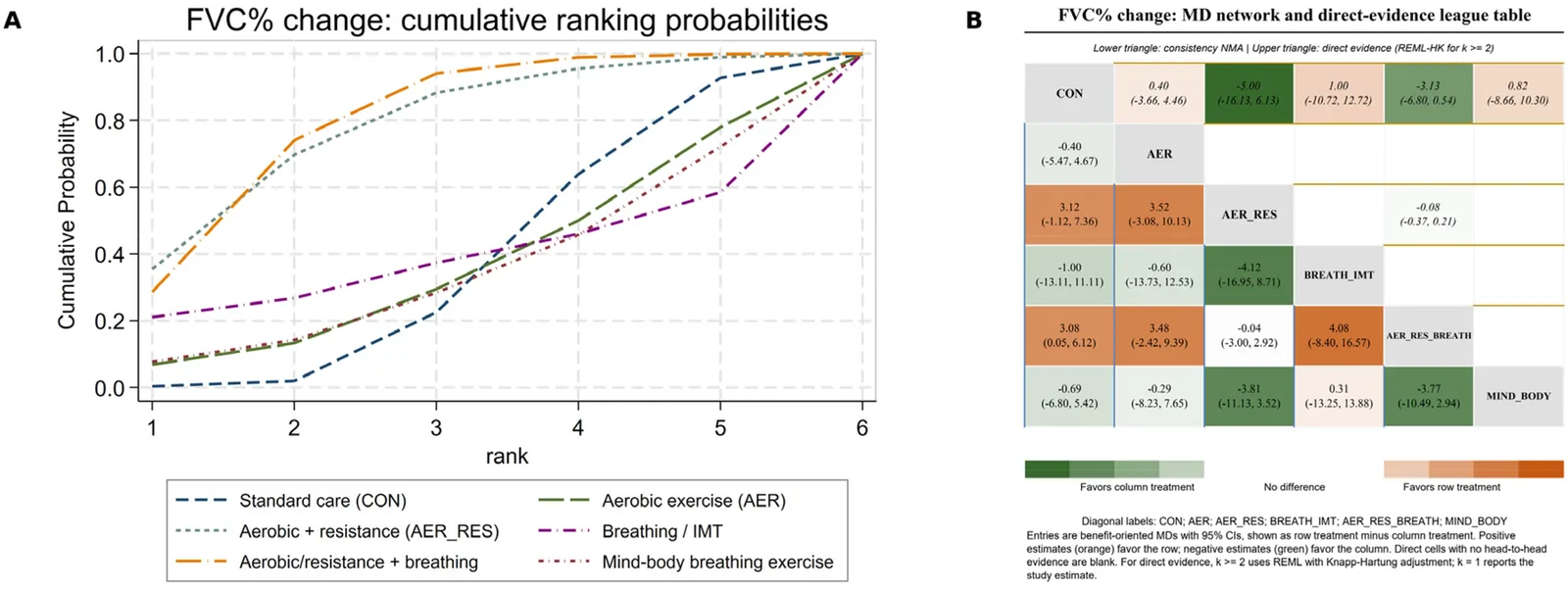
Main network meta-analysis results for percent-predicted forced vital capacity. **(A)** Cumulative ranking probability plot; and **(B)** league table of pairwise treatment comparisons. The lower triangle presents estimates from the random-effects network meta-analysis, whereas the upper triangle presents estimates from direct comparisons. Treatment effects are reported as mean differences with 95% confidence intervals. FVC, forced vital capacity; AER, aerobic training; AER_RES, combined aerobic and resistance training; BREATH_IMT, respiratory-focused training comprising breathing exercises or inspiratory muscle training; AER_RES_BREATH, combined aerobic, resistance, and breathing or inspiratory muscle training; MIND_BODY, mind–body exercise; CON, control; MD, mean difference; CI, confidence interval.

No clear differences were observed among the active interventions. In particular, the estimated difference between AER_RES_BREATH and AER_RES was close to zero (MD = −0.04 percentage points, 95% CI −3.00 to 2.92) (Figure 5B). AER_RES_BREATH and AER_RES had SUCRA values of 79.08% and 77.57%, respectively. However, given the similar relative effects and the low certainty of the evidence, these rankings should not be interpreted as indicating a definitive advantage in pulmonary function (Figure 5A; Supplementary Table S6).

### SGRQ total score

Changes in SGRQ scores were reverse-coded before analysis; therefore, a positive MD indicated a reduction in the SGRQ total score and an improvement in health-related quality of life. Compared with CON, AER_RES (MD = 10.08 points, 95% CI 2.83 to 17.33), AER_RES_BREATH (MD = 11.88 points, 95% CI 5.80 to 17.95), and MIND_BODY (MD = 15.20 points, 95% CI 0.18 to 30.22) were associated with greater improvements in the SGRQ total score. The confidence intervals for AER (MD = 6.55 points, 95% CI −1.57 to 14.66) and BREATH_IMT (MD = 6.60 points, 95% CI −1.85 to 15.06) included the null value (Figure 6B; Supplementary Figure S1C).

**Figure 6:**
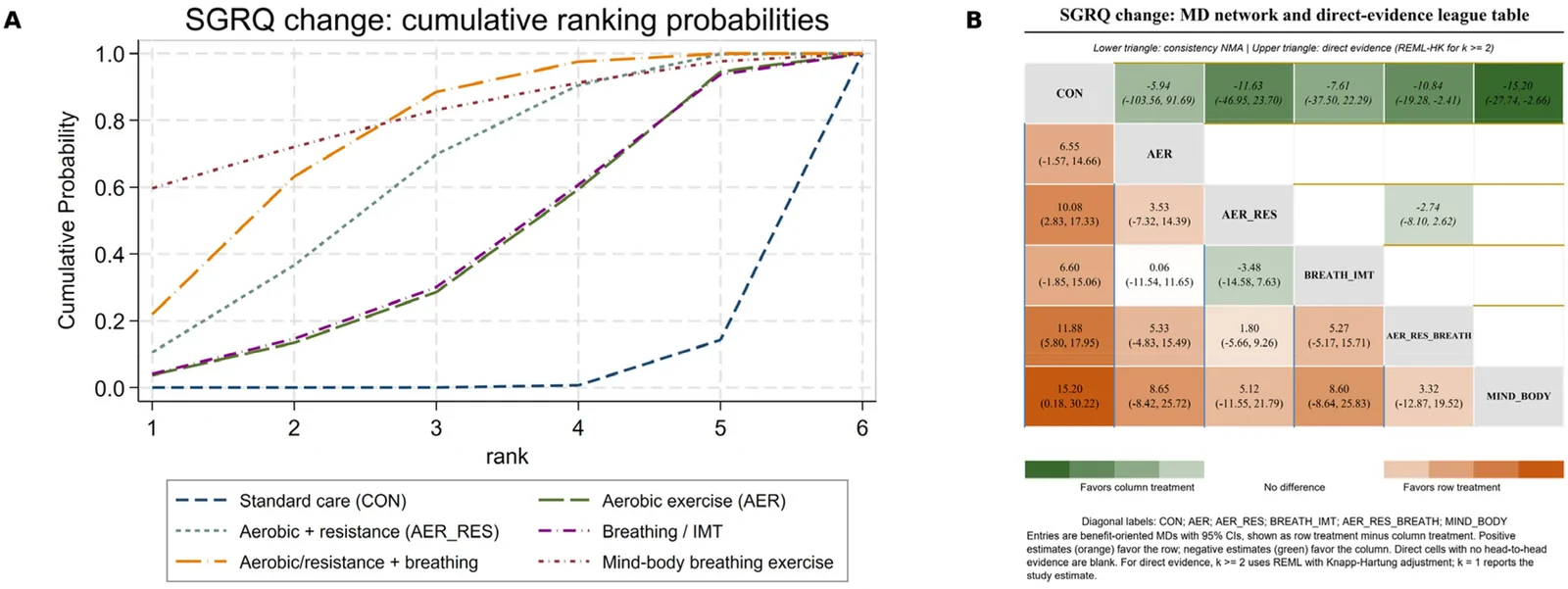
Main network meta-analysis results for the st george’s respiratory questionnaire total score. **(A)** Cumulative ranking probability plot; and **(B)** league table of pairwise treatment comparisons. The lower triangle presents estimates from the random-effects network meta-analysis, whereas the upper triangle presents estimates from direct comparisons. The direction of the St George’s Respiratory Questionnaire effect was reversed before analysis; therefore, positive mean differences indicate a greater reduction in the questionnaire score and favor the corresponding intervention. SGRQ, St George’s Respiratory Questionnaire; AER, aerobic training; AER_RES, combined aerobic and resistance training; BREATH_IMT, respiratory-focused training comprising breathing exercises or inspiratory muscle training; AER_RES_BREATH, combined aerobic, resistance, and breathing or inspiratory muscle training; MIND_BODY, mind–body exercise; CON, control; MD, mean difference; CI, confidence interval.

No clear differences were observed in any network comparison among the active interventions (Figure 6B). MIND_BODY had the highest SUCRA value at 80.75%, followed by AER_RES_BREATH at 74.22% and AER_RES at 61.47%. However, MIND_BODY was supported by only one analytical arm involving 14 participants in the SGRQ network, and its effect estimate had a wide confidence interval. Therefore, its apparent ranking should not be interpreted as evidence that it is superior to the other active interventions (Figure 6A; Supplementary Tables S1, S6).

### Sensitivity analyses

The robustness of the findings was evaluated using a fixed-effect MD model, a random-effects SMD model, leave-one-out analyses, and a restricted analysis including only trials that directly reported mean change scores and their corresponding standard deviations for all study arms.

For 6MWD, the primary-analysis estimate for AER_RES_BREATH versus CON was 57.12 m (95% CI 41.76 to 72.48). The corresponding fixed-effect MD was 50.74 m (95% CI 43.49 to 57.99), and the random-effects SMD was 1.05 (95% CI 0.71 to 1.40). After each of the 26 trials was excluded in turn, the MDs ranged from 52.93 to 63.10 m, and none of the 26 corresponding 95% CIs included the null value. The directions of effect and statistical conclusions for AER, AER_RES, and BREATH_IMT versus CON also remained unchanged. The direction of effect for MIND_BODY was consistent, although the confidence intervals included the null value in some leave-one-out analyses.

For FVC% predicted, the fixed-effect MD for AER_RES_BREATH versus CON was 2.00 percentage points (95% CI 1.03 to 2.96), and the random-effects SMD was 0.60 (95% CI 0.30 to 0.89). After each of the 11 trials was excluded in turn, the MDs ranged from 2.30 to 5.13 percentage points; however, only 7 of the 11 analyses yielded 95% CIs that excluded the null value, indicating that the random-effects primary estimate was sensitive to the exclusion of individual studies.

For the SGRQ total score, the fixed-effect MD for AER_RES_BREATH versus CON was 11.05 points (95% CI 7.93 to 14.17), and the random-effects SMD was 1.01 (95% CI 0.45 to 1.57). In the leave-one-out analyses of the 11 trials, the estimated improvement ranged from 10.40 to 13.52 points, and all 95% CIs excluded the null value. The direction of effect for AER_RES remained consistent, although the confidence intervals included the null value in some leave-one-out analyses.

When the analysis was restricted to trials in which all study arms directly reported both mean change scores and change-score standard deviations, the 6MWD network retained eight trials, 17 analytical arms, and 530 participants. The MD for AER_RES_BREATH versus CON was 56.28 m (95% CI 26.81 to 85.74). The FVC% predicted network retained four trials, eight analytical arms, and 188 participants. Because the between-study variance could not be identified, a fixed-effect model was used, yielding an MD of 5.84 percentage points for AER_RES_BREATH versus CON (95% CI 0.33 to 11.34). The SGRQ network retained five trials, 10 analytical arms, and 207 participants. Although the direction of effect for AER_RES_BREATH was consistent with the primary analysis, the confidence interval widened substantially and included the null value (MD = 9.69 points, 95% CI −10.39 to 29.77).

Overall, the 6MWD findings were the most robust across alternative models, effect measures, leave-one-out analyses, and the restricted data analysis. The primary SGRQ findings were generally stable under alternative models and leave-one-out analyses, although precision was substantially reduced in the restricted analysis because of the smaller number of studies and increased between-study heterogeneity. For FVC% predicted, the direction of effect was broadly consistent across several analyses; however, the lower confidence limit in the primary analysis was close to the null value, the leave-one-out results were not fully stable, and the restricted analysis required a fixed-effect model. These findings should therefore be interpreted cautiously (Supplementary Tables S2 and S3; Supplementary Figures S3–S7).

### Exploratory subgroup analyses and network meta-regression

Exploratory network meta-regression showed that the omnibus test for intervention duration was nominally statistically significant for 6MWD (*P* = 0.009), whereas no clear effect modification was observed for intervention setting (*P* = 0.906), disease type (*P* = 0.706), or level of supervision (*P* = 0.903). For FVC% predicted, none of the omnibus tests indicated clear effect modification by intervention setting (*P* = 0.631), intervention duration (*P* = 0.750), disease type (*P* = 0.559), or level of supervision (*P* = 0.631).

For the SGRQ total score, the omnibus tests for intervention setting (*P* = 0.013), disease type (*P* = 0.032), and level of supervision (*P* = 0.013) reached nominal statistical significance, whereas intervention duration showed no clear evidence of effect modification (*P* = 0.657). Treatment-specific coefficients suggested that the improvement associated with AER versus CON may have been smaller when the intervention was delivered at home or remotely than when it was delivered in a center or hospital (*β* = −15.37 points, 95% CI −26.59 to −4.16). The improvement associated with AER versus CON may also have been greater in studies involving other or mixed ILD populations than in studies restricted to patients with IPF (*β* = 15.37 points, 95% CI 4.16 to 26.59). Full results of the exploratory subgroup analyses and network meta-regressions are presented in Supplementary Table S4.

### Heterogeneity and inconsistency

Between-study heterogeneity was estimated as *τ* = 12.02 m and *τ*^2^ = 144.55 for the 6MWD network (*Q* = 28.07, df = 23, *P* = 0.213); *τ* = 1.55 percentage points and *τ*^2^ = 2.40 for the FVC% predicted network (*Q* = 5.57, df = 7, *P* = 0.591); and *τ* = 4.22 points and *τ*^2^ = 17.79 for the SGRQ total-score network (*Q* = 11.45, df = 7, *P* = 0.120).

The global inconsistency tests showed no statistically clear evidence of inconsistency for 6MWD (*χ*^2^ = 3.80, df = 3, *P* = 0.284), FVC% predicted (*χ*^2^ = 0.06, df = 1, *P* = 0.815), or the SGRQ total score (*χ*^2^ = 0.05, df = 1, *P* = 0.823). The minimum *P* values from the node-splitting analyses were 0.116, 0.750, and 0.822, respectively. However, because the networks contained few closed loops and limited direct comparisons among active interventions, particularly in the FVC% predicted and SGRQ networks, the statistical power of the inconsistency tests was limited. Accordingly, the absence of detected inconsistency should not be interpreted as definitive confirmation of the consistency assumption. Results of the heterogeneity and inconsistency assessments are presented in Supplementary Table S5, and prediction intervals are shown in Supplementary Figure S2.

### Publication bias

The comparison-adjusted funnel plots did not show clear systematic asymmetry for any of the three outcomes, and the study estimates for 6MWD appeared relatively symmetrically distributed. However, the FVC% predicted and SGRQ total-score analyses each included only 11 trials, limiting the ability of funnel plots to detect asymmetry or small-study effects. Therefore, although the available plots did not indicate clear small-study effects, publication bias could not be excluded (Figure 7).

**Figure 7:**
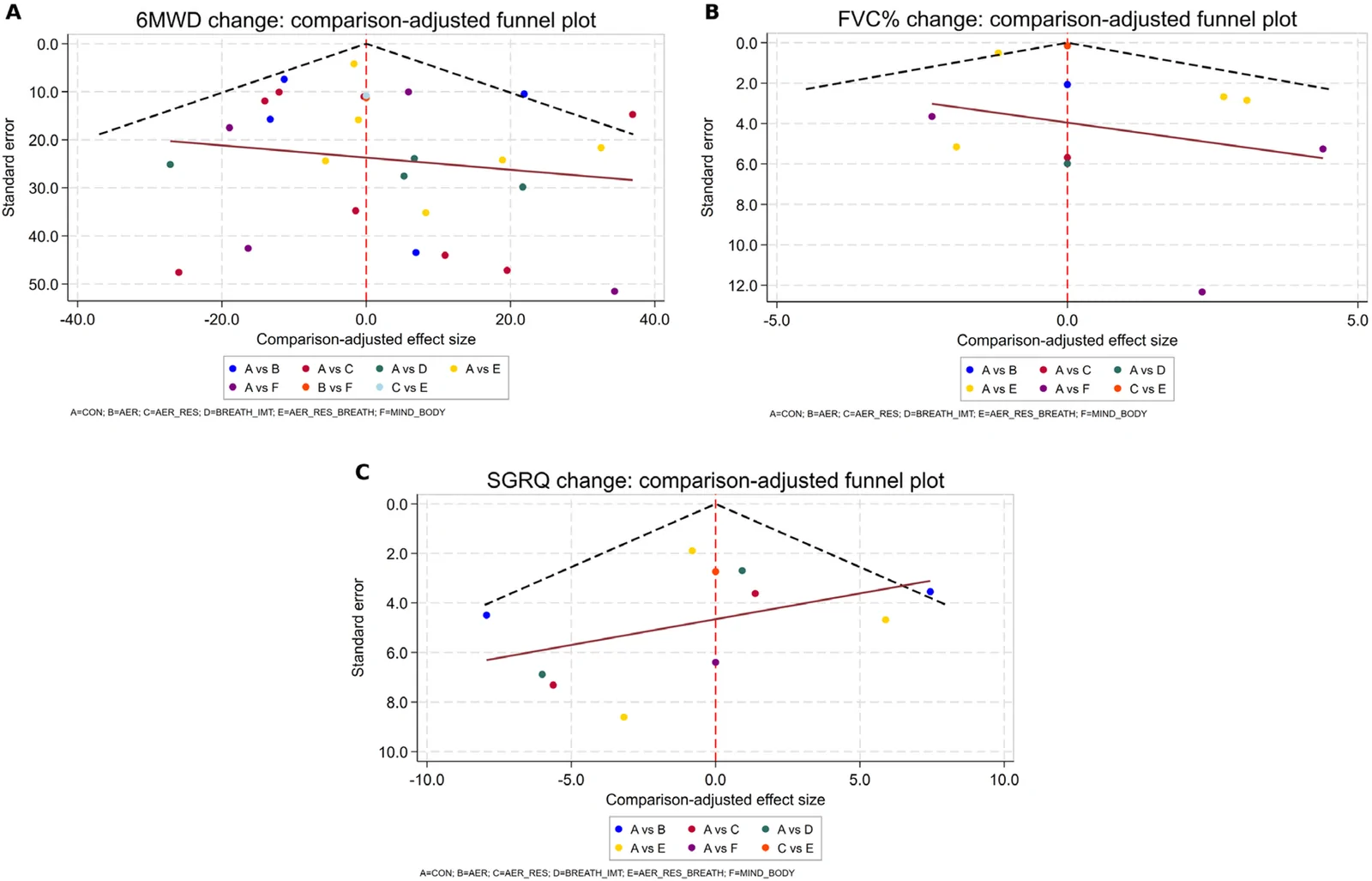
Comparison-adjusted funnel plots for the three primary outcomes. **(A)** Six-minute walk distance; **(B)** percent-predicted forced vital capacity; and **(C)** St George’s Respiratory Questionnaire total score. The vertical dashed line represents the null effect, and the diagonal dashed lines indicate the approximate 95% confidence limits. 6MWD, six-minute walk distance; FVC, forced vital capacity; SGRQ, St George’s Respiratory Questionnaire; MD, mean difference.

### Confidence in the evidence

The CINeMA assessment indicated that, for 6MWD, the confidence in the evidence was high for the comparison of BREATH_IMT versus CON and moderate for the comparisons of AER, AER_RES, AER_RES_BREATH, and MIND_BODY versus CON. For FVC% predicted, confidence in the evidence was low for AER_RES and AER_RES_BREATH versus CON and very low for AER, BREATH_IMT, and MIND_BODY versus CON. For the SGRQ total score, confidence in the evidence was moderate for AER_RES_BREATH versus CON, low for AER_RES, BREATH_IMT, and MIND_BODY versus CON, and very low for AER versus CON.

Downgrading was primarily attributable to within-study bias and imprecision. Concerns regarding within-study bias included inadequate reporting of randomization or allocation concealment, missing outcome data, and the potential influence of unblinded assessment on patient-reported or exercise performance outcomes. Imprecision was mainly related to the small number of studies and participants supporting some nodes, wide confidence intervals, and limited direct comparisons among active interventions. Most comparisons for FVC% predicted and the SGRQ total score were further downgraded because of the limited amount of information available. Accordingly, both relative treatment effects and intervention rankings should be interpreted in conjunction with the confidence in the evidence (Figure 8; Supplementary Figures S8 and S9).

**Figure 8:**
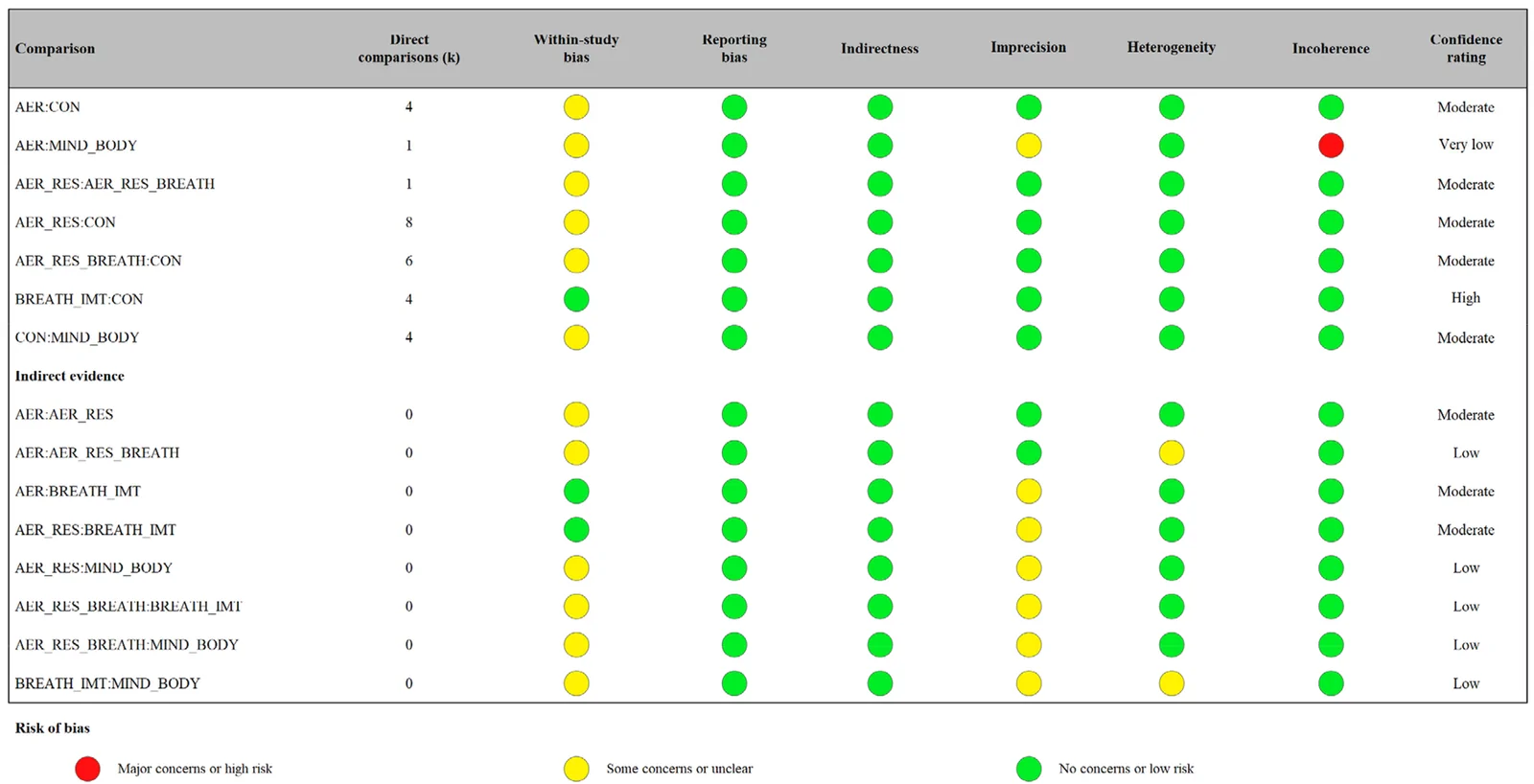
CINeMA assessment of confidence in the network meta-analysis results for the six-minute walk distance. Confidence was evaluated across six domains: within-study bias, reporting bias, indirectness, imprecision, heterogeneity, and incoherence. Green, yellow, and red circles indicate no concerns, some concerns, and major concerns, respectively. The overall confidence for each treatment comparison was rated as high, moderate, low, or very low. CINeMA, Confidence in Network Meta-Analysis; 6MWD, six-minute walk distance.

## Discussion

### Principal findings

This network meta-analysis included 26 randomized controlled trials and constructed the evidence network according to the active training components explicitly included in each intervention. All forms of structured exercise training were associated with improvements in six-minute walk distance (6MWD) among patients with interstitial lung disease (ILD). Combined aerobic, resistance, and breathing or inspiratory-muscle training (AER_RES_BREATH) produced the largest estimated effect and remained comparatively robust across multiple sensitivity analyses. AER_RES, AER_RES_BREATH, and mind–body exercise were associated with improvements in the St George's Respiratory Questionnaire (SGRQ) total score. For pulmonary function, the evidence was considerably less certain. Only AER_RES_BREATH showed a small difference in FVC% predicted compared with control, but this estimate had limited robustness and low confidence. Because exercise training primarily targets functional exercise capacity, peripheral and respiratory muscle function, and exercise efficiency rather than the underlying restrictive pulmonary impairment, substantial short-term improvements in FVC% predicted were not necessarily expected. Overall, the available evidence more strongly supports improvements in functional exercise capacity and health-related quality of life than improvements in pulmonary function. No single training program can yet be considered optimal for all patients with ILD.

### Comparison with previous evidence

These findings are broadly consistent with the previous Cochrane review and the American Thoracic Society clinical practice guideline, both of which support pulmonary rehabilitation for improving exercise capacity, symptoms, and quality of life in patients with ILD (6, 8). However, previous reviews generally grouped heterogeneous programs under the broad categories of pulmonary rehabilitation or exercise training and primarily addressed whether exercise was effective. By defining intervention nodes according to their actual training components, the present study further compared aerobic, resistance, breathing, and combined training and therefore extended the evidence question to which components might provide additional benefit. Because most of this incremental information was derived from indirect comparisons, it should be regarded as preliminary evidence for individualized exercise prescription rather than a definitive therapeutic hierarchy.

### Clinical interpretation and potential mechanisms

Among the evaluated outcomes, 6MWD had the largest evidence base and yielded the most robust findings. AER_RES_BREATH improved 6MWD by a mean of 57.12 m compared with control, exceeding the minimal important difference of approximately 29–34 m previously proposed for patients with ILD and suggesting a potentially clinically meaningful group-level effect (50). However, this threshold does not demonstrate that every patient achieved an important improvement and cannot replace an individual responder analysis. Exercise limitation in ILD is multifactorial and may involve impaired gas exchange, exertional hypoxemia, ventilatory and circulatory constraints, peripheral muscle dysfunction, and physical deconditioning (4, 51). Aerobic and resistance training primarily target cardiorespiratory endurance and peripheral muscle function, whereas respiratory-focused interventions may act through breathing-pattern regulation, ventilatory control, and/or improvements in respiratory-muscle performance. These complementary but not necessarily equivalent mechanisms may contribute to the functional effects observed with multicomponent exercise programs. Exercise training primarily targets functional reserve, peripheral and respiratory muscle function, and exercise efficiency rather than the underlying parenchymal abnormalities responsible for restrictive ventilatory impairment. Therefore, substantial short-term changes in FVC% predicted were not necessarily expected. The small difference observed for AER_RES_BREATH should accordingly be interpreted as an uncertain physiological signal rather than evidence that exercise training modifies the underlying disease process. Health-related quality of life is additionally influenced by dyspnea, fatigue, emotional status, and social participation and therefore should not be interpreted solely through changes in pulmonary function.

### Confidence in the network evidence

The findings should be interpreted in light of the structure of the evidence network. Most evidence was connected through the control node, whereas direct head-to-head evidence between active interventions was limited. Trials evaluating AER_RES_BREATH may also have differed from other comparisons in participant age, baseline disease severity, supervision, and inspiratory-muscle function. Consequently, the absence of detected statistical inconsistency does not fully establish the transitivity assumption. Surface under the cumulative ranking curve values and mean ranks represent relative ranking probabilities under the current model and evidence base. They should be interpreted together with effect sizes, confidence intervals, the amount of direct evidence, and CINeMA confidence ratings and should not independently determine the preferred intervention (23, 52).

### Strengths and limitations

The strengths of this review include its restriction to randomized controlled trials, definition of intervention nodes according to actual training components, use of original-scale effect estimates, and implementation of multiple sensitivity analyses and outcome-specific assessments of transitivity and confidence. Several limitations should nevertheless be acknowledged. The FVC% predicted and standard SGRQ total-score networks were sparse, and some nodes were supported by only a small number of studies. The subgroup analyses and network meta-regressions were also limited by the small number of studies and sparse network structure. Several study-level covariates overlapped substantially, and multiple omnibus and treatment-specific interaction tests were conducted without adjustment for multiple comparisons. These exploratory findings may therefore be susceptible to chance, ecological bias, and residual confounding and should be regarded as hypothesis-generating rather than definitive evidence of treatment-effect modification. Some trials had concerns related to inadequate reporting of randomization methods, missing outcome data, or unblinded outcome assessment. Some change-score data required transformation or estimation, while oxygen supplementation, exercise adherence, and adverse events were incompletely reported. Because breathing exercises and IMT differ in training intensity, mechanisms, and clinical application, their grouping may have introduced within-node clinical heterogeneity. Moreover, the analysis focused on outcomes measured immediately after the intervention and could not determine whether the observed benefits were maintained over time. Previous long-term evidence suggests that some rehabilitation benefits may diminish after supervised training is reduced or discontinued (41).

### Clinical and research implications

For patients with clinically stable ILD who are suitable for exercise, structured exercise training should be considered an important component of comprehensive disease management. Aerobic training may serve as a foundational component, with resistance or respiratory-muscle training added according to peripheral muscle weakness, inspiratory-muscle weakness, exertional hypoxemia, comorbidities, and patient preferences. A uniform program should not be prescribed to all patients solely on the basis of treatment rankings. Future adequately powered head-to-head trials should standardize the reporting of training frequency, intensity, time, type, progression, oxygen supplementation, achieved exercise dose, adherence, and safety. They should also report the proportion of patients achieving the minimal important difference and include long-term clinical outcomes. Factorial trials or component network meta-analysis may further distinguish the independent and synergistic effects of individual training components (53).

## Conclusion

Structured exercise training improves functional exercise capacity in patients with ILD and may also improve health-related quality of life, with combined aerobic, resistance, and breathing or inspiratory-muscle training showing the greatest potential benefit. However, evidence for improvement in pulmonary function remains limited, and comparisons between active interventions are largely based on indirect evidence. Given the sparse network structure, clinical heterogeneity, and low confidence in several outcome-specific estimates, the current evidence is insufficient to establish a single optimal training program for all patients with ILD. In clinical practice, aerobic exercise may serve as the foundation of training, with resistance and respiratory-muscle components individualized according to peripheral muscle strength, respiratory-muscle function, exertional hypoxemia, comorbidities, and patient preferences. Future adequately powered head-to-head randomized trials should use standardized intervention reporting and evaluate long-term effectiveness, adherence, safety, and individual clinically meaningful responses.

## Data Availability

Publicly available datasets were analyzed in this study. This data can be found here: Publicly available data were analyzed in this study. The data were extracted from the published studies cited in the article.

## References

[1] Maher TM. Interstitial lung disease: a review. Jama. (2024) 331(19):1655–65. 10.1001/jama.2024.3669 38648021

[2] Ma X Zhu L Kurche JS Xiao H Dai H Wang C. Global and regional burden of interstitial lung disease and pulmonary sarcoidosis from 1990 to 2019: results from the global burden of disease study 2019. Thorax. (2022) 77(6):596–605. 10.1136/thoraxjnl-2020-216732 34556551

[3] Zeng Q Jiang D. Global trends of interstitial lung diseases from 1990 to 2019: an age-period-cohort study based on the global burden of disease study 2019, and projections until 2030. Front Med (Lausanne). (2023) 10:1141372. 10.3389/fmed.2023.1141372 37554509 PMC10404716

[4] Holland AE. Exercise limitation in interstitial lung disease—mechanisms, significance and therapeutic options. Chron Respir Dis. (2010) 7(2):101–11. 10.1177/1479972309354689 20056733

[5] Panagiotou M Polychronopoulos V Strange C. Respiratory and lower limb muscle function in interstitial lung disease. Chron Respir Dis. (2016) 13(2):162–72. 10.1177/1479972315626014 26768011 PMC5734592

[6] Rochester CL Alison JA Carlin B Jenkins AR Cox NS Bauldoff G. Pulmonary rehabilitation for adults with chronic respiratory disease: an official American thoracic society clinical practice guideline. Am J Respir Crit Care Med. (2023) 208(4):e7–e26. 10.1164/rccm.202306-1066ST 37581410 PMC10449064

[7] Mendes RG Castello-Simões V Trimer R Garcia-Araújo AS Gonçalves Da Silva AL Dixit S. Exercise-based pulmonary rehabilitation for interstitial lung diseases: a review of components, prescription, efficacy, and safety. Front Rehabil Sci. (2021) 2:744102. 10.3389/fresc.2021.744102 36188788 PMC9397914

[8] Dowman L Hill CJ May A Holland AE. Pulmonary rehabilitation for interstitial lung disease. Cochrane Database Syst Rev. (2021) 2(2):Cd006322. 10.1002/14651858.CD006322.pub4 34559419 PMC8094410

[9] Reina-Gutiérrez S Torres-Costoso A Martínez-Vizcaíno V Núñez de Arenas-Arroyo S Fernández-Rodríguez R Pozuelo-Carrascosa DP. Effectiveness of pulmonary rehabilitation in interstitial lung disease, including coronavirus diseases: a systematic review and meta-analysis. Arch Phys Med Rehabil. (2021) 102(10):1989–97.e3. 10.1016/j.apmr.2021.03.035 PMC8474048 33932361

[10] Hoffman M. Inspiratory muscle training in interstitial lung disease: a systematic scoping review. J Bras Pneumol. (2021) 47(4):e20210089. 10.36416/1806-3756/e20210089 34495175 PMC8979670

[11] Fan J Chang Y Cheng S Liang B Qu D. Effect of breathing exercises on patients with interstitial lung disease: a systematic review and meta-analysis. Qual Life Res. (2024) 33(9):2335–47. 10.1007/s11136-024-03679-z 38907831

[12] Salazar JJ Mirza FT Uzzaman MN Shunmugam RH Shazana NZ Pinnock H. Characteristics of pulmonary rehabilitation programs and their effects on exercise capacity and health related quality of life (hrqol) in patients with interstitial lung disease: a systematic review and meta-analysis. Respir Med. (2025) 237:107936. 10.1016/j.rmed.2024.107936 39756486

[13] Song J Wang Q Zhao C Wang L Li L Kang D. Comparative efficacy of pulmonary rehabilitation delivery models on dyspnoea, exercise capacity and health-related quality of life in chronic respiratory disease: a systematic review and network meta-analysis. NPJ Prim Care Respir Med. (2025) 36(1):8. 10.1038/s41533-025-00472-4 41461647 PMC12852677

[14] Jansen JP Naci H. Is network meta-analysis as valid as standard pairwise meta-analysis? It all depends on the distribution of effect modifiers. BMC Med. (2013) 11:159. 10.1186/1741-7015-11-159 23826681 PMC3707819

[15] Page MJ McKenzie JE Bossuyt PM Boutron I Hoffmann TC Mulrow CD. The prisma 2020 statement: an updated guideline for reporting systematic reviews. Br Med J. (2021) 372:n71. 10.1136/bmj.n71 PMC8005924 33782057

[16] Hutton B Salanti G Caldwell DM Chaimani A Schmid CH Cameron C. The prisma extension statement for reporting of systematic reviews incorporating network meta-analyses of health care interventions: checklist and explanations. Ann Intern Med. (2015) 162(11):777–84. 10.7326/m14-2385 26030634

[17] Wan X Wang W Liu J Tong T. Estimating the sample mean and standard deviation from the sample size, median, range and/or interquartile range. BMC Med Res Methodol. (2014) 14:135. 10.1186/1471-2288-14-135 25524443 PMC4383202

[18] Follmann D Elliott P Suh I Cutler J. Variance imputation for overviews of clinical trials with continuous response. J Clin Epidemiol. (1992) 45(7):769–73. 10.1016/0895-4356(92)90054-q 1619456

[19] Sterne JAC Savović J Page MJ Elbers RG Blencowe NS Boutron I. Rob 2: a revised tool for assessing risk of bias in randomised trials. Br Med J. (2019) 366:l4898. 10.1136/bmj.l4898 31462531

[20] White IR. Network meta-analysis. Stata J. (2015) 15(4):951–85. 10.1177/1536867X1501500403

[21] Salanti G Ades AE Ioannidis JP. Graphical methods and numerical summaries for presenting results from multiple-treatment meta-analysis: an overview and tutorial. J Clin Epidemiol. (2011) 64(2):163–71. 10.1016/j.jclinepi.2010.03.016 20688472

[22] IntHout J Ioannidis JP Borm GF. The hartung-knapp-sidik-jonkman method for random effects meta-analysis is straightforward and considerably outperforms the standard dersimonian-laird method. BMC Med Res Methodol. (2014) 14:25. 10.1186/1471-2288-14-25 24548571 PMC4015721

[23] Nikolakopoulou A Higgins JPT Papakonstantinou T Chaimani A Del Giovane C Egger M. Cinema: an approach for assessing confidence in the results of a network meta-analysis. PLoS Med. (2020) 17(4):e1003082. 10.1371/journal.pmed.1003082 32243458 PMC7122720

[24] Holland AE Hill CJ Conron M Munro P McDonald CF. Short term improvement in exercise capacity and symptoms following exercise training in interstitial lung disease. Thorax. (2008) 63(6):549–54. 10.1136/thx.2007.088070 18245143

[25] Nishiyama O Kondoh Y Kimura T Kato K Kataoka K Ogawa T. Effects of pulmonary rehabilitation in patients with idiopathic pulmonary fibrosis. Respirology. (2008) 13(3):394–9. 10.1111/j.1440-1843.2007.01205.x 18399862

[26] Dale MT McKeough ZJ Munoz PA Corte P Bye PT Alison JA. Exercise training for asbestos-related and other dust-related respiratory diseases: a randomised controlled trial. BMC Pulm Med. (2014) 14:180. 10.1186/1471-2466-14-180 25407957 PMC4247671

[27] Jackson RM Gómez-Marín OW Ramos CF Sol CM Cohen MI Gaunaurd IA. Exercise limitation in ipf patients: a randomized trial of pulmonary rehabilitation. Lung. (2014) 192(3):367–76. 10.1007/s00408-014-9566-9 24705678

[28] Vainshelboim B Oliveira J Yehoshua L Weiss I Fox BD Fruchter O. Exercise training-based pulmonary rehabilitation program is clinically beneficial for idiopathic pulmonary fibrosis. Respiration. (2014) 88(5):378–88. 10.1159/000367899 25341682

[29] Karadallı MN Boşnak-Güçlü M Camcıoğlu B Kokturk N Türktaş H. Effects of inspiratory muscle training in subjects with sarcoidosis: a randomized controlled clinical trial. Respir Care. (2016) 61(4):483–94. 10.4187/respcare.04312 26715771

[30] Dowman LM McDonald CF Hill CJ Lee AL Barker K Boote C. The evidence of benefits of exercise training in interstitial lung disease: a randomised controlled trial. Thorax. (2017) 72(7):610–9. 10.1136/thoraxjnl-2016-208638 28213592

[31] Naz I Ozalevli S Ozkan S Sahin H. Efficacy of a structured exercise program for improving functional capacity and quality of life in patients with stage 3 and 4 sarcoidosis: a randomized controlled trial. J Cardiopulm Rehabil Prev. (2018) 38(2):124–30. 10.1097/hcr.0000000000000307 29401114

[32] Perez-Bogerd S Wuyts W Barbier V Demeyer H Van Muylem A Janssens W. Short and long-term effects of pulmonary rehabilitation in interstitial lung diseases: a randomised controlled trial. Respir Res. (2018) 19(1):182. 10.1186/s12931-018-0884-y 30236104 PMC6149060

[33] El-Komy HM Awad M Mansour W Elsayed EI. Impact of pulmonary rehabilitation on patients with interstitial lung diseases: an Egyptian experience. Egyptian Journal of Bronchology. (2019) 13(2):219–25. 10.4103/ejb.ejb_64_18

[34] Yuen HK Lowman JD Oster RA De Andrade JA. Home-based pulmonary rehabilitation for patients with idiopathic pulmonary fibrosis: a pilot study. J Cardiopulm Rehabil Prev. (2019) 39(4):281–4. 10.1097/hcr.0000000000000418 31241519

[35] Jarosch I Schneeberger T Gloeckl R Kreuter M Frankenberger M Neurohr C. Short-term effects of comprehensive pulmonary rehabilitation and its maintenance in patients with idiopathic pulmonary fibrosis: a randomized controlled trial. J Clin Med. (2020) 9(5):1567. 10.3390/jcm9051567 32455843 PMC7290850

[36] Cerdan-de-Las-Heras J Balbino F Løkke A Catalán-Matamoros D Hilberg O Bendstrup E. Tele-rehabilitation program in idiopathic pulmonary fibrosis—a single-center randomized trial. Int J Environ Res Public Health. (2021) 18(19):10016. 10.3390/ijerph181910016 34639313 PMC8508000

[37] Shen L Zhang Y Su Y Weng D Zhang F Wu Q. New pulmonary rehabilitation exercise for pulmonary fibrosis to improve the pulmonary function and quality of life of patients with idiopathic pulmonary fibrosis: a randomized control trial. Ann Palliat Med. (2021) 10(7):7289–97. 10.21037/apm-21-71 34353031

[38] Wang L Sun B Cui H Wang W Ren Q Sun Y. Long-term effects of home-based pulmonary rehabilitation on idiopathic interstitial pneumonia patients. All Life. (2021) 14(1):181–6. 10.1080/26895293.2021.1898051

[39] Zhou M Zhang H Li F Yu Z Yuan C Oliver B. Pulmonary daoyin as a traditional Chinese medicine rehabilitation programme for patients with ipf: a randomized controlled trial. Respirology. (2021) 26(4):360–9. 10.1111/resp.13972 33164264 PMC8048896

[40] Cerdan de Las Heras J Balbino F Catalán-Matamoros D Løkke A Hilberg O Bendstrup E. Effect of a telerehabilitation program in sarcoidosis. Sarcoidosis Vasc Diffuse Lung Dis. (2022) 39(1):e2022003. 10.36141/svdld.v39i1.12526 35494172 PMC9007024

[41] Kataoka K Nishiyama O Ogura T Mori Y Kozu R Arizono S. Long-term effect of pulmonary rehabilitation in idiopathic pulmonary fibrosis: a randomised controlled trial. Thorax. (2023) 78(8):784–91. 10.1136/thorax-2022-219792 37012071

[42] Zaki S Moiz JA Mujaddadi A Ali MS Talwar D. Does inspiratory muscle training provide additional benefits during pulmonary rehabilitation in people with interstitial lung disease? A randomized control trial. Physiother Theory Pract. (2023) 39(3):518–28. 10.1080/09593985.2021.2024311 35001815

[43] Aktan R Tertemiz KC Yiğit S Özalevli S Ozgen Alpaydin A Uçan ES. Effects of home-based telerehabilitation-assisted inspiratory muscle training in patients with idiopathic pulmonary fibrosis: a randomized controlled trial. Respirology. (2024) 29(12):1077–84. 10.1111/resp.14810 39129185

[44] Atmaca SN Demir R Mustafaoğlu R Atahan E. The effect of Baduanjin exercises on the functional capacity, respiratory function, and quality of life of patients with idiopathic pulmonary fibrosis: a randomized controlled trial. Biopsychosoc Med. (2025) 19(1):24. 10.1186/s13030-025-00346-8 41184899 PMC12581411

[45] Kadura S Purkayastha S Benditt J Anand A Collins B Quadros MD. Yoga effect on quality-of-life study among patients with idiopathic pulmonary fibrosis (Yes-Ipf). medRxiv (2025) 25327762. 10.1101/2025.05.20.25327762 42874938

[46] Arslan MS Ozer AY Bingöl Z Okumuş G. Effectiveness of active video games as a training tool in patients with interstitial lung disease: a randomised controlled trial. Respirology. (2026) 31(3):265–75. 10.1002/resp.70166 41344887

[47] Vicenzi M Bellocchi C Deboeck G Beretta L Tobaldini E Montano N. Inspiratory muscle training improves exercise performance and breathing pattern during 6-min walking test in systemic sclerosis-associated interstitial lung disease. ERJ Open Res. (2026) 12(2):00659-2025. 10.1183/23120541.00659-2025 42111381 PMC13152346

[48] Buschulte K Kotzerke M Waßmuth L Ganter C Koczulla R Höger P. Yoga in fibrosing interstitial lung diseases: a feasibility trial. Pneumologie. (2026). 10.1055/a-2831-6649 41927024

[49] Zou S Ye S Luo C Lin B Deng W Hua H. Effects of individualized pulmonary rehabilitation on respiratory function and exercise endurance in pneumoconiosis patients. BMC Pulm Med. (2026) 26(1):164. 10.1186/s12890-026-04105-5 41514419 PMC13067624

[50] Holland AE Hill CJ Conron M Munro P McDonald CF. Small changes in six-minute walk distance are important in diffuse parenchymal lung disease. Respir Med. (2009) 103(10):1430–5. 10.1016/j.rmed.2009.04.024 19477109

[51] Bonini M Fiorenzano G. Exertional dyspnoea in interstitial lung diseases: the clinical utility of cardiopulmonary exercise testing. Eur Respir Rev. (2017) 26(143):160099. 10.1183/16000617.0099-2016 28223398 PMC9489119

[52] Chiocchia V White IR Salanti G. The complexity underlying treatment rankings: how to use them and what to look at. BMJ Evid Based Med. (2023) 28(3):180–2. 10.1136/bmjebm-2021-111904 35501121 PMC7614655

[53] Ward TJC Latimer L Daynes E Freeman SC Ward S Xu J. Impact of pulmonary rehabilitation programme design on effectiveness in COPD: a systematic review and component network meta-analysis. eClinicalMed. (2025) 87:103433. 10.1016/j.eclinm.2025.103433 PMC12396583 40896469

