# Supplementary material for: Comparative effects of different exercise training modalities on exercise capacity, pulmonary function, and health-related quality of life in patients with interstitial lung disease: a systematic review and network meta-analysis
Source: Front Med (Lausanne). 2026 Oct 8;13:1953668. doi: 10.3389/fmed.2026.1953668 (PMC13687353; doi:10.3389/fmed.2026.1953668)
Supplement: Supplementary file 1 [file Supplementaryfile1.docx]

Supporting Information

**Comparative Effectiveness of Different Exercise Training Modalities for Exercise Capacity, Pulmonary Function, and Health-Related Quality of Life in Interstitial Lung Disease: A Systematic Review and Network Meta-Analysis**

# Contents

| **Item** | **English title** |
| --- | --- |
| Supplementary Appendix S1 | Full search strategies for all databases |
| Supplementary Appendix S2 | Intervention classification, outcome harmonization, and data transformation methods |
| Supplementary Table S1 | Treatment-node definitions and network composition |
| Supplementary Table S2 | Sensitivity analyses using alternative effect models and exclusion of non-directly reported change data |
| Supplementary Table S3 | Leave-one-out sensitivity analyses for the three primary outcomes |
| Supplementary Table S4 | Subgroup analyses and network meta-regression results |
| Supplementary Table S5 | Heterogeneity, global inconsistency, and node-splitting analyses |
| Supplementary Table S6 | Treatment-ranking results for the three primary outcomes |
| Supplementary Figure S1 | Random-effects network meta-analysis forest plots |
| Supplementary Figure S2 | Prediction-interval plots |
| Supplementary Figure S3 | Leave-one-out sensitivity analyses across all intervention nodes |
| Supplementary Figure S4 | Detailed leave-one-out analysis for 6MWD: AER_RES_BREATH versus CON |
| Supplementary Figure S5 | Detailed leave-one-out analysis for FVC% predicted: AER_RES_BREATH versus CON |
| Supplementary Figure S6 | Detailed leave-one-out analysis for SGRQ Total: AER_RES_BREATH versus CON |
| Supplementary Figure S7 | Primary analyses versus analyses restricted to directly reported change data |
| Supplementary Figure S8 | CINeMA assessment for FVC% predicted |
| Supplementary Figure S9 | CINeMA assessment for SGRQ Total |

# Supplementary Appendix S1. Full Search Strategies for All Databases

| **Database / Date of search** | **Search string** | **Results** |
| --- | --- | --- |
| PubMed June 11, 2026 | #1 ("Lung Diseases, Interstitial"[MeSH Terms] OR "Idiopathic Pulmonary Fibrosis"[MeSH Terms] OR "Pulmonary Fibrosis"[MeSH Terms] OR "Alveolitis, Extrinsic Allergic"[MeSH Terms] OR "Pneumoconiosis"[MeSH Terms] OR "Sarcoidosis, Pulmonary"[MeSH Terms] OR "Lung Diseases, Interstitial"[Title/Abstract] OR "Diffuse Parenchymal Lung Diseases"[Title/Abstract] OR "Interstitial Lung Diseases"[Title/Abstract] OR "Interstitial Lung Disease"[Title/Abstract] OR "Lung Disease, Interstitial"[Title/Abstract] OR "Diffuse Parenchymal Lung Disease"[Title/Abstract] OR "Pneumonia,  Pneumonias"[Title/Abstract] OR "Pneumonias, Interstitial"[Title/Abstract] OR "Pneumonitis,  Pneumonitis"[Title/Abstract] OR "Pneumonitides, Interstitial"[Title/Abstract] OR "Idiopathic Pulmonary Fibrosis"[Title/Abstract] OR "Idiopathic Pulmonary Fibroses"[Title/Abstract] OR "Pulmonary Fibroses, Idiopathic"[Title/Abstract] OR "Cryptogenic Fibrosing Alveolitis"[Title/Abstract] OR "Cryptogenic Fibrosing Alveolitides"[Title/Abstract] OR "Fibrosing Alveolitides, Cryptogenic"[Title/Abstract] OR "Pulmonary Fibrosis, Idiopathic"[Title/Abstract] OR "Fibrosing Alveolitis, Cryptogenic"[Title/Abstract] OR "Fibrocystic Pulmonary Dysplasia"[Title/Abstract] OR "Dysplasia, Fibrocystic Pulmonary"[Title/Abstract] OR "Fibrocystic Pulmonary Dysplasias"[Title/Abstract] OR "Pulmonary Dysplasia, Fibrocystic"[Title/Abstract] OR "Idiopathic Fibrosing Alveolitis, Chronic Form"[Title/Abstract] OR "Familial Idiopathic Pulmonary Fibrosis"[Title/Abstract] OR "Idiopathic Pulmonary Fibrosis, Familial"[Title/Abstract] OR "Usual Interstitial Pneumonia"[Title/Abstract] OR "Interstitial Pneumonia, Usual"[Title/Abstract] OR "Usual Interstitial Pneumonias"[Title/Abstract] OR "Interstitial Pneumonitis, Usual"[Title/Abstract] OR "Pneumonitides, Usual Interstitial"[Title/Abstract] OR "Pneumonitis, Usual Interstitial"[Title/Abstract] OR "Usual Interstitial Pneumonitides"[Title/Abstract] OR "Usual Interstitial Pneumonitis"[Title/Abstract] OR "Pulmonary Fibrosis"[Title/Abstract] OR "Fibroses, Pulmonary"[Title/Abstract] OR "Fibrosis, Pulmonary"[Title/Abstract] OR "Pulmonary Fibroses"[Title/Abstract] OR "Alveolitis, Fibrosing"[Title/Abstract] OR "Alveolitides,  Alveolitis"[Title/Abstract] OR "Idiopathic Diffuse Interstitial Pulmonary Fibrosis"[Title/Abstract] OR "Alveolitis, Extrinsic Allergic"[Title/Abstract] OR "Allergic Alveolitis, Extrinsic"[Title/Abstract] OR "Allergic Alveolitides, Extrinsic"[Title/Abstract] OR "Alveolitides, Extrinsic Allergic"[Title/Abstract] OR "Extrinsic Allergic Alveolitides"[Title/Abstract] OR "Extrinsic Allergic Alveolitis"[Title/Abstract] OR "Hypersensitivity Pneumonitis"[Title/Abstract] OR "Hypersensitivity Pneumonitides"[Title/Abstract] OR "Pneumonitides, Hypersensitivity"[Title/Abstract] OR "Pneumonitis, Hypersensitivity"[Title/Abstract] OR "Pneumoconiosis"[Title/Abstract] OR "Pneumoconioses"[Title/Abstract] OR "Bagassosis"[Title/Abstract] OR "Sarcoidosis, Pulmonary"[Title/Abstract] OR "Pulmonary Sarcoidosis"[Title/Abstract] OR "Pulmonary Sarcoidoses"[Title/Abstract] OR "Sarcoidoses, Pulmonary"[Title/Abstract]) #2 ("Respiratory Therapy"[MeSH Terms] OR "Exercise"[MeSH Terms] OR "Exercise Therapy"[MeSH Terms] OR "Breathing Exercises"[MeSH Terms] OR "High-Intensity Interval Training"[MeSH Terms] OR "Resistance Training"[MeSH Terms] OR "Oxygen Inhalation Therapy"[MeSH Terms] OR "Mind-Body Therapies"[MeSH Terms] OR "Walking"[MeSH Terms] OR "Running"[MeSH Terms] OR "Swimming"[MeSH Terms] OR "Jogging"[MeSH Terms] OR "Tai Ji"[MeSH Terms] OR "Qigong"[MeSH Terms] OR "Yoga"[MeSH Terms] OR "Bicycling"[MeSH Terms] OR "Daoyin"[MeSH Terms] OR "Rehabilitation"[MeSH Terms] OR "Endurance Training"[MeSH Terms] OR "Exercise Movement Techniques"[MeSH Terms] OR "Physical Fitness"[MeSH Terms] OR "Physical Exertion"[MeSH Terms] OR  Inhalation"[Title/Abstract] OR "Exercise"[Title/Abstract] OR "Exercises"[Title/Abstract] OR "Exercise, Physical"[Title/Abstract] OR "Exercises, Physical"[Title/Abstract] OR "Physical Exercise"[Title/Abstract] OR "Physical Exercises"[Title/Abstract] OR "Exercise, Isometric"[Title/Abstract] OR "Exercises, Isometric"[Title/Abstract] OR "Isometric Exercises"[Title/Abstract] OR "Isometric Exercise"[Title/Abstract] OR "Exercise, Aerobic"[Title/Abstract] OR "Aerobic Exercise"[Title/Abstract] OR "Aerobic  Exercises"[Title/Abstract] OR "Exercises, Aerobic"[Title/Abstract] OR "Exercise Training"[Title/Abstract] OR "Exercise Trainings"[Title/Abstract] OR "Training, Exercise"[Title/Abstract] OR "Trainings, Exercise"[Title/Abstract] OR "Physical Activity"[Title/Abstract] OR "Activities, Physical"[Title/Abstract] OR "Activity, Physical"[Title/Abstract] OR "Physical Activities"[Title/Abstract] OR "Active Breaks"[Title/Abstract] OR "Activity Breaks"[Title/Abstract] OR "Acute Exercise"[Title/Abstract] OR "Acute Exercises"[Title/Abstract] OR "Exercise, Acute"[Title/Abstract] OR "Exercises, Acute"[Title/Abstract] OR "Exercise Therapy"[Title/Abstract] OR "Exercise Prescription"[Title/Abstract] OR "Rehabilitation  Rehabilitation"[Title/Abstract] OR "Rehabilitation Exercises"[Title/Abstract] OR "Remedial Exercise"[Title/Abstract] OR "Exercise, Remedial"[Title/Abstract] OR "Exercises, Remedial"[Title/Abstract] OR "Remedial Exercises"[Title/Abstract] OR "Therapy, Exercise"[Title/Abstract] OR "Exercise Therapies"[Title/Abstract] OR "Therapies, Exercise"[Title/Abstract] OR "Physical Activity  Breathing"[Title/Abstract] OR "Respiratory Muscle Training"[Title/Abstract] OR "Muscle Training, Respiratory"[Title/Abstract] OR "Training, Respiratory Muscle"[Title/Abstract] OR "High-Intensity Interval Training"[Title/Abstract] OR "High Intensity Interval Training"[Title/Abstract] OR "High-Intensity Interval Trainings"[Title/Abstract] OR "Interval Training, High-Intensity"[Title/Abstract] OR "Interval Trainings, High-Intensity"[Title/Abstract] OR "Training, High-Intensity Interval"[Title/Abstract] OR "Trainings, High-Intensity Interval"[Title/Abstract] OR "High-Intensity Intermittent Exercise"[Title/Abstract] OR "Exercise, High-Intensity Intermittent"[Title/Abstract] OR "Exercises, High-Intensity Intermittent"[Title/Abstract] OR "High-Intensity Intermittent Exercises"[Title/Abstract] OR "Sprint Interval Training"[Title/Abstract] OR "Sprint Interval Trainings"[Title/Abstract] OR "Resistance Training"[Title/Abstract] OR "Training, Resistance"[Title/Abstract] OR "Strength Training"[Title/Abstract] OR "Training, Strength"[Title/Abstract] OR "Weight-Lifting Strengthening Program"[Title/Abstract] OR "Strengthening Programs, Weight-Lifting"[Title/Abstract] OR "Strengthening Program, Weight-Lifting"[Title/Abstract] OR "Weight Lifting Strengthening Program"[Title/Abstract] OR "Weight-Lifting Strengthening Programs"[Title/Abstract] OR "Weight-Lifting Exercise Program"[Title/Abstract] OR "Exercise Programs, Weight-Lifting"[Title/Abstract] OR "Exercise Program, Weight-Lifting"[Title/Abstract] OR "Weight Lifting Exercise Program"[Title/Abstract] OR "Weight-Lifting Exercise Programs"[Title/Abstract] OR "Weight-Bearing Strengthening Program"[Title/Abstract] OR "Strengthening Programs, Weight-Bearing"[Title/Abstract] OR "Strengthening Program, Weight-Bearing"[Title/Abstract] OR "Weight Bearing Strengthening Program"[Title/Abstract] OR "Weight-Bearing Strengthening Programs"[Title/Abstract] OR "Weight-Bearing Exercise Program"[Title/Abstract] OR "Exercise Programs, Weight-Bearing"[Title/Abstract] OR "Exercise Program, Weight-Bearing"[Title/Abstract] OR "Weight Bearing Exercise Program"[Title/Abstract] OR "Weight-Bearing Exercise Programs"[Title/Abstract] OR "Oxygen Inhalation Therapy"[Title/Abstract] OR "Inhalation Therapy, Oxygen"[Title/Abstract] OR "Inhalation Therapies, Oxygen"[Title/Abstract] OR "Oxygen Inhalation Therapies"[Title/Abstract] OR "Therapies, Oxygen Inhalation"[Title/Abstract] OR "Therapy, Oxygen Inhalation"[Title/Abstract] OR "Mind-Body Therapies"[Title/Abstract] OR "Mind Body  Medicine"[Title/Abstract] OR "Mind Body Medicine"[Title/Abstract] OR "Walking"[Title/Abstract] OR "Ambulation"[Title/Abstract] OR "Running"[Title/Abstract] OR "Runnings"[Title/Abstract] OR "Swimming"[Title/Abstract] OR "Jogging"[Title/Abstract] OR "Joggings"[Title/Abstract] OR "Tai Ji"[Title/Abstract] OR "Tai-ji"[Title/Abstract] OR "Tai Chi"[Title/Abstract] OR "Chi, Tai"[Title/Abstract] OR "Tai Chi Chuan"[Title/Abstract] OR "Taiji"[Title/Abstract] OR "Taijiquan"[Title/Abstract] OR "T'ai Chi"[Title/Abstract] OR "Tai Ji Quan"[Title/Abstract] OR "Ji Quan, Tai"[Title/Abstract] OR "Quan, Tai Ji"[Title/Abstract] OR "Qigong"[Title/Abstract] OR "Ch'i Kung"[Title/Abstract] OR "Qi  "Daoyin"[Title/Abstract] OR "Rehabilitation"[Title/Abstract] OR "Habilitation"[Title/Abstract] OR "Endurance Training"[Title/Abstract] OR "Training, Endurance"[Title/Abstract] OR "Exercise Movement Techniques"[Title/Abstract] OR "Movement Techniques, Exercise"[Title/Abstract] OR "Exercise Movement  Pilates-Based"[Title/Abstract] OR "Pilates Based Exercises"[Title/Abstract] OR "Pilates Training"[Title/Abstract] OR "Training, Pilates"[Title/Abstract] OR "Physical Fitness"[Title/Abstract] OR "Fitness, Physical"[Title/Abstract] OR "Physical Exertion"[Title/Abstract] OR "Exertion, Physical"[Title/Abstract] OR "Exertions, Physical"[Title/Abstract] OR "Physical Exertions"[Title/Abstract] OR "Exercise Intensity"[Title/Abstract] OR "Physical Effort"[Title/Abstract] OR "Effort, Physical"[Title/Abstract] OR "Efforts, Physical"[Title/Abstract] OR "Oxygen"[Title/Abstract] OR "Oxygen-16"[Title/Abstract] OR "Oxygen 16"[Title/Abstract] OR "Dioxygen"[Title/Abstract]) #3 ("Randomized Controlled Trial"[Publication Type] OR "Controlled Clinical Trial"[Publication Type] OR "Clinical Trial"[Publication Type] OR "Cross-Over Studies"[Mesh]) #4 #1 AND #2 AND #3 | 737 |
| Web of Science June 11, 2026 | #1 TS=("Lung Diseases, Interstitial" OR "Diffuse Parenchymal Lung Diseases" OR "Interstitial Lung Diseases" OR "Interstitial Lung Disease" OR "Lung Disease, Interstitial" OR "Diffuse Parenchymal Lung Disease" OR "Pneumonia, Interstitial" OR "Interstitial Pneumonia" OR "Interstitial Pneumonias" OR "Pneumonias, Interstitial" OR "Pneumonitis, Interstitial" OR "Interstitial Pneumonitides" OR "Interstitial Pneumonitis" OR "Pneumonitides, Interstitial" OR "Idiopathic Pulmonary Fibrosis" OR "Idiopathic Pulmonary  Fibroses" OR "Pulmonary Fibroses, Idiopathic" OR "Cryptogenic Fibrosing Alveolitis" OR "Cryptogenic Fibrosing Alveolitides" OR "Fibrosing Alveolitides, Cryptogenic" OR "Pulmonary Fibrosis, Idiopathic" OR "Fibrosing Alveolitis, Cryptogenic" OR "Fibrocystic Pulmonary Dysplasia" OR "Dysplasia, Fibrocystic Pulmonary" OR "Fibrocystic Pulmonary Dysplasias" OR "Pulmonary Dysplasia, Fibrocystic" OR "Idiopathic Fibrosing Alveolitis, Chronic Form" OR "Familial Idiopathic Pulmonary Fibrosis" OR "Idiopathic Pulmonary Fibrosis, Familial" OR "Usual Interstitial Pneumonia" OR "Interstitial Pneumonia, Usual" OR "Usual Interstitial Pneumonias" OR "Interstitial Pneumonitis, Usual" OR "Pneumonitides, Usual Interstitial" OR "Pneumonitis, Usual Interstitial" OR "Usual Interstitial Pneumonitides" OR "Usual Interstitial Pneumonitis" OR "Pulmonary Fibrosis" OR "Fibroses, Pulmonary" OR "Fibrosis, Pulmonary" OR "Pulmonary Fibroses" OR "Alveolitis, Fibrosing" OR "Alveolitides, Fibrosing" OR "Fibrosing Alveolitides" OR "Fibrosing Alveolitis" OR "Idiopathic Diffuse Interstitial Pulmonary Fibrosis" OR "Alveolitis, Extrinsic Allergic" OR "Allergic Alveolitis, Extrinsic" OR "Allergic Alveolitides, Extrinsic" OR "Alveolitides, Extrinsic Allergic" OR "Extrinsic Allergic Alveolitides" OR "Extrinsic Allergic Alveolitis" OR "Hypersensitivity Pneumonitis" OR "Hypersensitivity Pneumonitides" OR "Pneumonitides, Hypersensitivity" OR "Pneumonitis, Hypersensitivity" OR "Pneumoconiosis" OR "Pneumoconioses" OR "Bagassosis" OR "Sarcoidosis, Pulmonary" OR "Pulmonary Sarcoidosis" OR "Pulmonary Sarcoidoses" OR "Sarcoidoses, Pulmonary") #2 TS=("Respiratory Therapy" OR "Therapy, Respiratory" OR "Respiratory Therapies" OR "Therapies, Respiratory" OR "Inhalation Therapy" OR "Therapy, Inhalation" OR "Inhalation Therapies" OR "Therapies, Inhalation" OR "Exercise" OR "Exercises" OR "Exercise, Physical" OR "Exercises, Physical" OR "Physical Exercise" OR "Physical Exercises" OR "Exercise, Isometric" OR "Exercises, Isometric" OR "Isometric Exercises" OR "Isometric Exercise" OR "Exercise, Aerobic" OR "Aerobic Exercise" OR "Aerobic Exercises" OR "Exercises, Aerobic" OR "Exercise Training" OR "Exercise Trainings" OR "Training, Exercise" OR "Trainings, Exercise" OR "Physical Activity" OR "Activities, Physical" OR "Activity, Physical" OR "Physical Activities" OR "Active Breaks" OR "Activity Breaks" OR "Acute Exercise" OR "Acute Exercises" OR "Exercise, Acute" OR "Exercises, Acute" OR "Exercise Therapy" OR "Exercise Prescription" OR "Rehabilitation Exercise" OR "Exercise, Rehabilitation" OR "Exercises, Rehabilitation" OR "Rehabilitation Exercises" OR "Remedial Exercise" OR "Exercise, Remedial" OR "Exercises, Remedial" OR "Remedial Exercises" OR "Therapy, Exercise" OR "Exercise Therapies" OR "Therapies, Exercise" OR "Physical Activity Intervention" OR "Breathing Exercises" OR "Exercise, Breathing" OR "Respiratory Muscle Training" OR "Muscle Training, Respiratory" OR "Training, Respiratory Muscle" OR "High-Intensity Interval Training" OR "High Intensity Interval Training" OR "High-Intensity Interval Trainings" OR "Interval Training, High-Intensity" OR "Interval Trainings, High-Intensity" OR "Training, High-Intensity Interval" OR "Trainings, High-Intensity Interval" OR "High-Intensity Intermittent Exercise" OR "Exercise, High-Intensity Intermittent" OR "Exercises, High-Intensity Intermittent" OR "High-Intensity Intermittent Exercises" OR "Sprint Interval Training" OR "Sprint Interval Trainings" OR "Resistance Training" OR "Training, Resistance" OR "Strength Training" OR "Training, Strength" OR "Weight-Lifting Strengthening Program" OR "Strengthening Programs, Weight-Lifting" OR "Strengthening Program, Weight-Lifting" OR "Weight Lifting Strengthening Program" OR "Weight-Lifting Strengthening Programs" OR "Weight-Lifting Exercise Program" OR "Exercise Programs, Weight-Lifting" OR "Exercise Program, Weight-Lifting" OR "Weight Lifting Exercise Program" OR "Weight-Lifting Exercise Programs" OR "Weight-Bearing Strengthening Program" OR "Strengthening Programs, Weight-Bearing" OR "Strengthening Program, Weight-Bearing" OR "Weight Bearing Strengthening Program" OR "Weight-Bearing Strengthening Programs" OR "Weight-Bearing Exercise Program" OR "Exercise Programs, Weight-Bearing" OR "Exercise Program, Weight-Bearing" OR "Weight Bearing Exercise Program" OR "Weight-Bearing Exercise Programs" OR "Oxygen Inhalation Therapy" OR "Inhalation Therapy, Oxygen" OR "Inhalation Therapies, Oxygen" OR "Oxygen Inhalation Therapies" OR "Therapies, Oxygen Inhalation" OR "Therapy, Oxygen Inhalation" OR "Mind-Body Therapies" OR "Mind Body Therapies" OR "Mind-Body Therapy" OR "Therapies, Mind-Body" OR "Therapy, Mind-Body" OR "Mind-Body Medicine" OR "Mind Body Medicine" OR "Walking" OR "Ambulation" OR "Running" OR "Runnings" OR "Swimming" OR "Jogging" OR "Joggings" OR "Tai Ji" OR "Tai-ji" OR "Tai Chi" OR "Chi, Tai" OR "Tai Chi Chuan" OR "Taiji" OR "Taijiquan" OR "T'ai Chi" OR "Tai Ji Quan" OR "Ji Quan, Tai" OR "Quan, Tai Ji" OR "Qigong" OR "Ch'i Kung" OR "Qi Gong" OR "Yoga" OR "Bicycling" OR "Daoyin" OR "Rehabilitation" OR "Habilitation" OR "Endurance Training" OR "Training, Endurance" OR "Exercise Movement Techniques" OR "Movement Techniques, Exercise" OR "Exercise Movement Technics" OR "Pilates-Based Exercises" OR "Exercises, Pilates-Based" OR "Pilates Based Exercises" OR "Pilates Training" OR "Training, Pilates" OR "Physical Fitness" OR "Fitness, Physical" OR "Physical Exertion" OR "Exertion, Physical" OR "Exertions, Physical" OR "Physical Exertions" OR "Exercise Intensity" OR "Physical Effort" OR "Effort, Physical" OR "Efforts, Physical" OR "Oxygen" OR "Oxygen-16" OR "Oxygen 16" OR "Dioxygen") #3 TS=("Randomized Controlled Trial" OR "Controlled Clinical Trial" OR "Clinical Trial" OR "Cross-Over Studies") #4 #1 AND #2 AND #3 | 398 |
| Scopus June 11, 2026 | #1 TITLE-ABS-KEY("Lung Diseases, Interstitial" OR "Diffuse Parenchymal Lung Diseases" OR "Interstitial Lung Diseases" OR "Interstitial Lung Disease" OR "Lung Disease, Interstitial" OR "Diffuse Parenchymal Lung Disease" OR "Pneumonia, Interstitial" OR "Interstitial Pneumonia" OR "Interstitial Pneumonias" OR "Pneumonias, Interstitial" OR "Pneumonitis, Interstitial" OR "Interstitial Pneumonitides" OR "Interstitial Pneumonitis" OR "Pneumonitides, Interstitial" OR "Idiopathic Pulmonary Fibrosis" OR "Idiopathic Pulmonary Fibroses" OR "Pulmonary Fibroses, Idiopathic" OR "Cryptogenic Fibrosing Alveolitis" OR "Cryptogenic  Fibrosing Alveolitides" OR "Fibrosing Alveolitides, Cryptogenic" OR "Pulmonary Fibrosis, Idiopathic" OR "Fibrosing Alveolitis, Cryptogenic" OR "Fibrocystic Pulmonary Dysplasia" OR "Dysplasia, Fibrocystic Pulmonary" OR "Fibrocystic Pulmonary Dysplasias" OR "Pulmonary Dysplasia, Fibrocystic" OR "Idiopathic Fibrosing Alveolitis, Chronic Form" OR "Familial Idiopathic Pulmonary Fibrosis" OR "Idiopathic Pulmonary Fibrosis, Familial" OR "Usual Interstitial Pneumonia" OR "Interstitial Pneumonia, Usual" OR "Usual Interstitial Pneumonias" OR "Interstitial Pneumonitis, Usual" OR "Pneumonitides, Usual Interstitial" OR "Pneumonitis, Usual Interstitial" OR "Usual Interstitial Pneumonitides" OR "Usual Interstitial Pneumonitis" OR "Pulmonary Fibrosis" OR "Fibroses, Pulmonary" OR "Fibrosis, Pulmonary" OR "Pulmonary Fibroses" OR "Alveolitis, Fibrosing" OR "Alveolitides, Fibrosing" OR "Fibrosing Alveolitides" OR "Fibrosing Alveolitis" OR "Idiopathic Diffuse Interstitial Pulmonary Fibrosis" OR "Alveolitis, Extrinsic Allergic" OR "Allergic Alveolitis, Extrinsic" OR "Allergic Alveolitides, Extrinsic" OR "Alveolitides, Extrinsic Allergic" OR "Extrinsic Allergic Alveolitides" OR "Extrinsic Allergic Alveolitis" OR "Hypersensitivity Pneumonitis" OR "Hypersensitivity Pneumonitides" OR "Pneumonitides, Hypersensitivity" OR "Pneumonitis, Hypersensitivity" OR "Pneumoconiosis" OR "Pneumoconioses" OR "Bagassosis" OR "Sarcoidosis, Pulmonary" OR "Pulmonary Sarcoidosis" OR "Pulmonary Sarcoidoses" OR "Sarcoidoses, Pulmonary") #2 TITLE-ABS-KEY("Respiratory Therapy" OR "Therapy, Respiratory" OR "Respiratory Therapies" OR "Therapies, Respiratory" OR "Inhalation Therapy" OR "Therapy, Inhalation" OR "Inhalation Therapies" OR "Therapies, Inhalation" OR "Exercise" OR "Exercises" OR "Exercise, Physical" OR "Exercises, Physical" OR "Physical Exercise" OR "Physical Exercises" OR "Exercise, Isometric" OR "Exercises, Isometric" OR "Isometric Exercises" OR "Isometric Exercise" OR "Exercise, Aerobic" OR "Aerobic Exercise" OR "Aerobic Exercises" OR "Exercises, Aerobic" OR "Exercise Training" OR "Exercise Trainings" OR "Training, Exercise" OR "Trainings, Exercise" OR "Physical Activity" OR "Activities, Physical" OR "Activity, Physical" OR "Physical Activities" OR "Active Breaks" OR "Activity Breaks" OR "Acute Exercise" OR "Acute Exercises" OR "Exercise, Acute" OR "Exercises, Acute" OR "Exercise Therapy" OR "Exercise Prescription" OR "Rehabilitation Exercise" OR "Exercise, Rehabilitation" OR "Exercises, Rehabilitation" OR "Rehabilitation Exercises" OR "Remedial Exercise" OR "Exercise, Remedial" OR "Exercises, Remedial" OR "Remedial Exercises" OR "Therapy, Exercise" OR "Exercise Therapies" OR "Therapies, Exercise" OR "Physical Activity Intervention" OR "Breathing Exercises" OR "Exercise, Breathing" OR "Respiratory Muscle Training" OR "Muscle Training, Respiratory" OR "Training, Respiratory Muscle" OR "High-Intensity Interval Training" OR "High Intensity Interval Training" OR "High-Intensity Interval Trainings" OR "Interval Training, High-Intensity" OR "Interval Trainings, High-Intensity" OR "Training, High-Intensity Interval" OR "Trainings, High-Intensity Interval" OR "High-Intensity Intermittent Exercise" OR "Exercise, High-Intensity Intermittent" OR "Exercises, High-Intensity Intermittent" OR "High-Intensity Intermittent Exercises" OR "Sprint Interval Training" OR "Sprint Interval Trainings" OR "Resistance Training" OR "Training, Resistance" OR "Strength Training" OR "Training, Strength" OR "Weight-Lifting Strengthening Program" OR "Strengthening Programs, Weight-Lifting" OR "Strengthening Program, Weight-Lifting" OR "Weight Lifting Strengthening Program" OR "Weight-Lifting Strengthening Programs" OR "Weight-Lifting Exercise Program" OR "Exercise Programs, Weight-Lifting" OR "Exercise Program, Weight-Lifting" OR "Weight Lifting Exercise Program" OR "Weight-Lifting Exercise Programs" OR "Weight-Bearing Strengthening Program" OR "Strengthening Programs, Weight-Bearing" OR "Strengthening Program, Weight-Bearing" OR "Weight Bearing Strengthening Program" OR "Weight-Bearing Strengthening Programs" OR "Weight-Bearing Exercise Program" OR "Exercise Programs, Weight-Bearing" OR "Exercise Program, Weight-Bearing" OR "Weight Bearing Exercise Program" OR "Weight-Bearing Exercise Programs" OR "Oxygen Inhalation Therapy" OR "Inhalation Therapy, Oxygen" OR "Inhalation Therapies, Oxygen" OR "Oxygen Inhalation Therapies" OR "Therapies, Oxygen Inhalation" OR "Therapy, Oxygen Inhalation" OR "Mind-Body Therapies" OR "Mind Body Therapies" OR "Mind-Body Therapy" OR "Therapies, Mind-Body" OR "Therapy, Mind-Body" OR "Mind-Body Medicine" OR "Mind Body Medicine" OR "Walking" OR "Ambulation" OR "Running" OR "Runnings" OR "Swimming" OR "Jogging" OR "Joggings" OR "Tai Ji" OR "Tai-ji" OR "Tai Chi" OR "Chi, Tai" OR "Tai Chi Chuan" OR "Taiji" OR "Taijiquan" OR "T'ai Chi" OR "Tai Ji Quan" OR "Ji Quan, Tai" OR "Quan, Tai Ji" OR "Qigong" OR "Ch'i Kung" OR "Qi Gong" OR "Yoga" OR "Bicycling" OR "Daoyin" OR "Rehabilitation" OR "Habilitation" OR "Endurance Training" OR "Training, Endurance" OR "Exercise Movement Techniques" OR "Movement Techniques, Exercise" OR "Exercise Movement Technics" OR "Pilates-Based Exercises" OR "Exercises, Pilates-Based" OR "Pilates Based Exercises" OR "Pilates Training" OR "Training, Pilates" OR "Physical Fitness" OR "Fitness, Physical" OR "Physical Exertion" OR "Exertion, Physical" OR "Exertions, Physical" OR "Physical Exertions" OR "Exercise Intensity" OR "Physical Effort" OR "Effort, Physical" OR "Efforts, Physical" OR "Oxygen" OR "Oxygen-16" OR "Oxygen 16" OR "Dioxygen") #3 TITLE-ABS-KEY("Randomized Controlled Trial" OR "Controlled Clinical Trial" OR "Clinical Trial" OR "Cross-Over Studies") #4 #1 AND #2 AND #3 | 1,690 |
| Embase June 11, 2026 | #1 ('respiratory care'/exp OR 'Exercise'/exp OR 'Kinesiotherapy'/exp OR 'breathing exercise'/exp OR 'high intensity interval training'/exp OR 'resistance training'/exp OR 'oxygen therapy'/exp OR 'alternative medicine'/exp OR 'Walking'/exp OR 'Running'/exp OR 'Swimming'/exp OR 'Jogging'/exp OR 'Tai Chi'/exp OR 'Qigong'/exp OR 'Yoga'/exp OR 'Cycling'/exp OR 'Daoyin'/exp OR 'Rehabilitation'/exp OR 'endurance training'/exp OR 'Fitness'/exp OR 'oxygen'/exp) #2 ('respiratory care' OR 'respiration care' OR 'respiratory therapy' OR 'Exercise' OR 'biometric exercise' OR 'effort' OR 'exercise capacity' OR 'exercise performance' OR 'exercise training' OR 'exertion' OR 'fitness  training' OR 'fitness workout' OR 'physical conditioning, human' OR 'physical effort' OR 'physical exercise' OR 'physical exertion' OR 'physical work-out' OR 'physical workout' OR 'Kinesiotherapy' OR 'corrective exercise' OR 'exercise movement techniques' OR 'exercise therapy' OR 'exercise treatment' OR 'kinesiotherapeutic intervention' OR 'kinesiotherapeutic method' OR 'kinesiotherapeutic procedure' OR 'kinesiotherapeutic technique' OR 'kinesiotherapeutical treatment' OR 'kinesitherapeutic exercises' OR 'kinesitherapeutic intervention' OR 'kinesitherapeutic method' OR 'kinesitherapeutic methodology' OR 'kinesitherapeutic procedure' OR 'kinesitherapeutic technique' OR 'kinesitherapeutic treatment' OR 'kinesitherapeutical treatment' OR 'kinesitherapy' OR 'SKTM (specialized kinesitherapeutic methodology)' OR 'specialised kinesitherapeutic methodology' OR 'specialized kinesitherapeutic methodology' OR 'therapeutic exercise' OR 'therapy, exercise' OR 'treatment, exercise' OR 'breathing exercise' OR 'breathing exercises' OR 'breathing therapy' OR 'chest physical therapy' OR 'chest physiotherapy' OR 'exercise, breathing' OR 'exercise, respiratory' OR 'respiration exercise' OR 'respiration therapy' OR 'respiratory exercise' OR 'respiratory physiotherapy' OR 'high intensity interval training' OR 'high-intensity intermittent exercise' OR 'high-intensity intermittent training' OR 'high-intensity interval exercise' OR 'high-intensity interval training' OR 'HIIE (exercise)' OR 'HIIT' OR 'intermittent high-intensity training' OR 'interval high-intensity training' OR 'resistance training' OR 'resistance exercise' OR 'resistance exercise training' OR 'resistance-type exercise' OR 'resistance-type training' OR 'strength training' OR 'strength-type exercise' OR 'strength-type training' OR 'oxygen therapy' OR 'o2 administration' OR 'o2 therapy' OR 'oxygen administration' OR 'oxygen inhalation therapy' OR 'oxygen insufflation' OR 'oxygen treatment' OR 'alternative medicine' OR 'alternative therapies' OR 'alternative therapy' OR 'complementary medicine' OR 'complementary therapies' OR 'eclectic medicine' OR 'eclecticism, historical' OR 'historical eclecticism' OR 'mental healing' OR 'metaphysical mind-body relations' OR 'mind body technique' OR 'mind body therapies' OR 'mind body therapy' OR 'mind-body relations (metaphysics)' OR 'mind-body relations, metaphysical' OR 'mind-body therapies' OR 'polarity therapy' OR 'radiaesthesia' OR 'radiesthesia' OR 'therapeutic cults' OR 'Thomsonian medicine' OR 'Walking' OR 'forest walking' OR 'Running' OR 'Swimming' OR 'bathing beaches' OR 'swimmer' OR 'swimming test' OR 'Jogging' OR 'Tai Chi' OR 'Tai Chi Chuan' OR 'Tai Ji' OR 'Taiji quan' OR 'Taijiquan' OR 'Qigong' OR 'chi kung' OR 'chigung' OR 'qi gong' OR 'Yoga' OR 'yogic meditation' OR 'Cycling' OR 'bicycling' OR 'Daoyin' OR 'Rehabilitation' OR 'functional readaptation' OR 'medical rehabilitation' OR 'readaption' OR 'readjustment' OR 'rehabilitation concept' OR 'rehabilitation engineering' OR 'rehabilitation potential' OR 'rehabilitation process' OR 'rehabilitation program' OR 'rehabilitation programme' OR 'rehabilitation, medical' OR 'rehabilitational program' OR 'rehabilitational programme' OR 'rehabilitational treatment' OR 'rehabilitative treatment' OR 'resocialisation' OR 'resocialisation therapy' OR 'resocialization' OR 'resocialization therapy' OR 'revalidation' OR 'endurance training' OR 'endurance exercise' OR 'endurance exercise training' OR 'endurance workout' OR 'endurance-type exercise' OR 'endurance-type training' OR 'Fitness' OR 'fitness, physical' OR 'physical fitness' OR 'oxygen' OR '16O' OR 'air oxygen' OR 'dioxygen' OR 'liquid oxygen' OR 'O 16' OR 'O 2' OR 'O2' OR 'oxigen' OR 'oxygen 16' OR 'oxygen ion' OR 'oxygen isotope' OR 'oxygen isotopes' OR 'oxygen radioisotopes'):ti,ab,kw #3 #1 OR #2 #4 ('interstitial lung disease'/exp OR 'fibrosing alveolitis'/exp OR 'lung fibrosis'/exp OR 'allergic pneumonitis'/exp OR 'Pneumoconiosis'/exp OR 'lung sarcoidosis'/exp) #5 ('interstitial lung disease' OR 'diffuse interstitial pneumopathy' OR 'diffuse parenchyma lung disease' OR 'diffuse parenchymal lung disease' OR 'diffuse parenchymal pulmonary disease' OR 'diffuse parenchymal pulmonary disorder' OR 'interstitial lung diseases' OR 'interstitial lung disorder' OR 'interstitial pneumopathy' OR 'interstitial pulmonary disease' OR 'interstitial pulmonary disorder' OR 'lung disease, interstitial' OR 'lung diseases, interstitial' OR 'pneumopathy, interstitial' OR 'fibrosing alveolitis' OR 'alveolitis, fibrosing' OR 'chronic diffuse interstitial fibrosis' OR 'chronic interstitial pneumonia' OR 'cryptogenic fibrosing alveolitis' OR 'diffuse interstitial fibrosis' OR 'diffuse interstitial lung fibrosis' OR 'diffuse interstitial pulmonary fibrosis' OR 'diffuse lung fibrosis' OR 'diffuse pulmonary fibrosis' OR 'hamman rich disease' OR 'Hamman Rich syndrome' OR 'Hamman-Rich syndrome' OR 'idiopathic fibrosing alveolitis' OR 'idiopathic interstitial fibrosis' OR 'idiopathic interstitial lung fibrosis' OR 'idiopathic lung fibrosis' OR 'idiopathic pulmonary fibrosis' OR 'interstitial fibrosis (lung)' OR 'interstitial lung fibrosis' OR 'interstitial pulmonary fibrosis' OR 'IPF (idiopathic pulmonary fibrosis)' OR 'lung alveolar fibrosis' OR 'lung fibrosing alveolitis' OR 'lung fibrosis, interstitial' OR 'lung interstitial fibrosis' OR 'pulmonary fibrosis, interstitial' OR 'pulmonary interstitial fibrosis' OR 'lung fibrosis' OR 'fibrosis pulmonis' OR 'fibrosis, lung' OR 'lung parenchymal fibrosis' OR 'lung sclerosis' OR 'pneumosclerosis' OR 'pulmonary fibrosis' OR 'pulmonary sclerosis' OR 'allergic pneumonitis' OR 'allergic alveolitis' OR 'allergic alveolitis, extrinsic' OR 'allergic extrinsic alveolitis' OR 'allergic pneumonia' OR 'alveolitis, allergic' OR 'alveolitis, extrinsic allergic' OR 'extrinsic allergic alveolitis' OR 'hypersensitive pneumonitis' OR 'hypersensitivity alveolitis' OR 'hypersensitivity pneumonitis' OR 'pneumonitis, allergic' OR 'Pneumoconiosis' OR 'Caplan Colinet syndrome' OR 'Caplan disease' OR 'Caplan syndrome' OR 'Caplan`s syndrome' OR 'disease, caplan' OR 'lung coniosis' OR 'pneumoconiosis, rheumatoid' OR 'pneumoconiotic lesion' OR 'pneumokoniosis' OR 'pneumonoconiosis' OR 'pneumonokoniosis' OR 'rheumatoid pneumoconiosis' OR 'silicoarthritis' OR 'syndrome, caplan' OR 'lung sarcoidosis' OR 'fibrotic pulmonary sarcoidosis' OR 'lung sarcoid' OR 'pulmonary fibrotic sarcoidosis' OR 'pulmonary sarcoid disease' OR 'pulmonary sarcoidosis' OR 'sarcoid lung' OR 'sarcoid lung disease' OR 'sarcoidosis of the lung' OR 'sarcoidosis pulmonum' OR 'sarcoidosis, pulmonary'):ti,ab,kw #6 #4 OR #5 #7 ('randomized controlled trial'/exp OR 'controlled clinical trial'/exp OR 'clinical trial'/exp OR 'crossover  procedure'/exp OR ('randomized controlled trial' OR 'controlled clinical trial' OR 'clinical trial' OR 'cross-over studies'):ti,ab,kw) #8 #3 AND #6 AND #7 | 3,138 |
| Cochrane Library June 11, 2026 | #1 ([mh "Respiratory Therapy"] OR [mh "Exercise"] OR [mh "Exercise Therapy"] OR [mh "Breathing Exercises"] OR [mh "High-Intensity Interval Training"] OR [mh "Resistance Training"] OR [mh "Oxygen Inhalation Therapy"] OR [mh "Mind-Body Therapies"] OR [mh "Walking"] OR [mh "Running"] OR [mh "Swimming"] OR [mh "Jogging"] OR [mh "Tai Ji"] OR [mh "Qigong"] OR [mh "Yoga"] OR [mh "Bicycling"] OR [mh "Daoyin"] OR [mh "Rehabilitation"] OR [mh "Endurance Training"] OR [mh "Exercise Movement Techniques"] OR [mh "Physical Fitness"] OR [mh "Physical Exertion"] OR [mh "Oxygen"]) #2 ("Respiratory Therapy" OR "Therapy, Respiratory" OR "Respiratory Therapies" OR "Therapies, Respiratory" OR "Inhalation Therapy" OR "Therapy, Inhalation" OR "Inhalation Therapies" OR "Therapies, Inhalation" OR "Exercise" OR "Exercises" OR "Exercise, Physical" OR "Exercises, Physical" OR "Physical Exercise" OR "Physical Exercises" OR "Exercise, Isometric" OR "Exercises, Isometric" OR "Isometric Exercises" OR "Isometric Exercise" OR "Exercise, Aerobic" OR "Aerobic Exercise" OR "Aerobic Exercises" OR "Exercises, Aerobic" OR "Exercise Training" OR "Exercise Trainings" OR "Training, Exercise" OR "Trainings, Exercise" OR "Physical Activity" OR "Activities, Physical" OR "Activity, Physical" OR "Physical Activities" OR "Active Breaks" OR "Activity Breaks" OR "Acute Exercise" OR "Acute Exercises" OR "Exercise, Acute" OR "Exercises, Acute" OR "Exercise Therapy" OR "Exercise Prescription" OR "Rehabilitation Exercise" OR "Exercise, Rehabilitation" OR "Exercises, Rehabilitation" OR "Rehabilitation Exercises" OR "Remedial Exercise" OR "Exercise, Remedial" OR "Exercises, Remedial" OR "Remedial Exercises" OR "Therapy, Exercise" OR "Exercise Therapies" OR "Therapies, Exercise" OR "Physical Activity Intervention" OR "Breathing Exercises" OR "Exercise, Breathing" OR "Respiratory Muscle Training" OR "Muscle Training, Respiratory" OR "Training, Respiratory Muscle" OR "High-Intensity Interval Training" OR "High Intensity Interval Training" OR "High-Intensity Interval Trainings" OR "Interval Training, High-Intensity" OR "Interval Trainings, High-Intensity" OR "Training, High-Intensity Interval" OR "Trainings, High-Intensity Interval" OR "High-Intensity Intermittent Exercise" OR "Exercise, High-Intensity Intermittent" OR "Exercises, High-Intensity Intermittent" OR "High-Intensity Intermittent Exercises" OR "Sprint Interval Training" OR "Sprint Interval Trainings" OR "Resistance Training" OR "Training, Resistance" OR "Strength Training" OR "Training, Strength" OR "Weight-Lifting Strengthening Program" OR "Strengthening Programs, Weight-Lifting" OR "Strengthening Program, Weight-Lifting" OR "Weight Lifting Strengthening Program" OR "Weight-Lifting Strengthening Programs" OR "Weight-Lifting Exercise Program" OR "Exercise Programs, Weight-Lifting" OR "Exercise Program, Weight-Lifting" OR "Weight Lifting Exercise Program" OR "Weight-Lifting Exercise Programs" OR "Weight-Bearing Strengthening Program" OR "Strengthening Programs, Weight-Bearing" OR "Strengthening Program, Weight-Bearing" OR "Weight Bearing Strengthening Program" OR "Weight-Bearing Strengthening Programs" OR "Weight-Bearing Exercise Program" OR "Exercise Programs, Weight-Bearing" OR "Exercise Program, Weight-Bearing" OR "Weight Bearing Exercise Program" OR "Weight-Bearing Exercise Programs" OR "Oxygen Inhalation Therapy" OR "Inhalation Therapy, Oxygen" OR "Inhalation Therapies, Oxygen" OR "Oxygen Inhalation Therapies" OR "Therapies, Oxygen Inhalation" OR "Therapy, Oxygen Inhalation" OR "Mind-Body Therapies" OR "Mind Body Therapies" OR "Mind-Body Therapy" OR "Therapies, Mind-Body" OR "Therapy, Mind-Body" OR "Mind-Body Medicine" OR "Mind Body Medicine" OR "Walking" OR "Ambulation" OR "Running" OR "Runnings" OR "Swimming" OR "Jogging" OR "Joggings" OR "Tai Ji" OR "Tai-ji" OR "Tai Chi" OR "Chi, Tai" OR "Tai Chi Chuan" OR "Taiji" OR "Taijiquan" OR "T'ai Chi" OR "Tai Ji Quan" OR "Ji Quan, Tai" OR "Quan, Tai Ji" OR "Qigong" OR "Ch'i Kung" OR "Qi Gong" OR "Yoga" OR "Bicycling" OR "Daoyin" OR "Rehabilitation" OR "Habilitation" OR "Endurance Training" OR "Training, Endurance" OR "Exercise Movement Techniques" OR "Movement Techniques, Exercise" OR "Exercise Movement Technics" OR "Pilates-Based Exercises" OR "Exercises, Pilates-Based" OR "Pilates Based Exercises" OR "Pilates Training" OR "Training, Pilates" OR "Physical Fitness" OR "Fitness, Physical" OR "Physical Exertion" OR "Exertion, Physical" OR "Exertions, Physical" OR "Physical Exertions" OR "Exercise Intensity" OR "Physical Effort" OR "Effort, Physical" OR "Efforts, Physical" OR "Oxygen" OR "Oxygen-16" OR "Oxygen 16" OR "Dioxygen"):ti,ab,kw #3 #1 OR #2 #4 ([mh "Lung Diseases, Interstitial"] OR [mh "Idiopathic Pulmonary Fibrosis"] OR [mh "Pulmonary Fibrosis"] OR [mh "Alveolitis, Extrinsic Allergic"] OR [mh "Pneumoconiosis"] OR [mh "Sarcoidosis, Pulmonary"]) #5 ("Lung Diseases, Interstitial" OR "Diffuse Parenchymal Lung Diseases" OR "Interstitial Lung Diseases" OR "Interstitial Lung Disease" OR "Lung Disease, Interstitial" OR "Diffuse Parenchymal Lung Disease" OR "Pneumonia, Interstitial" OR "Interstitial Pneumonia" OR "Interstitial Pneumonias" OR "Pneumonias, Interstitial" OR "Pneumonitis, Interstitial" OR "Interstitial Pneumonitides" OR "Interstitial Pneumonitis" OR "Pneumonitides, Interstitial" OR "Idiopathic Pulmonary Fibrosis" OR "Idiopathic Pulmonary Fibroses" OR "Pulmonary Fibroses, Idiopathic" OR "Cryptogenic Fibrosing Alveolitis" OR "Cryptogenic Fibrosing Alveolitides" OR "Fibrosing Alveolitides, Cryptogenic" OR "Pulmonary Fibrosis, Idiopathic" OR "Fibrosing Alveolitis, Cryptogenic" OR "Fibrocystic Pulmonary Dysplasia" OR "Dysplasia, Fibrocystic Pulmonary" OR "Fibrocystic Pulmonary Dysplasias" OR "Pulmonary Dysplasia, Fibrocystic" OR "Idiopathic Fibrosing Alveolitis, Chronic Form" OR "Familial Idiopathic Pulmonary Fibrosis" OR "Idiopathic Pulmonary Fibrosis, Familial" OR "Usual Interstitial Pneumonia" OR "Interstitial Pneumonia, Usual" OR "Usual Interstitial Pneumonias" OR "Interstitial Pneumonitis, Usual" OR "Pneumonitides, Usual Interstitial" OR "Pneumonitis,  Usual Interstitial" OR "Usual Interstitial Pneumonitides" OR "Usual Interstitial Pneumonitis" OR "Pulmonary Fibrosis" OR "Fibroses, Pulmonary" OR "Fibrosis, Pulmonary" OR "Pulmonary Fibroses" OR "Alveolitis, Fibrosing" OR "Alveolitides, Fibrosing" OR "Fibrosing Alveolitides" OR "Fibrosing Alveolitis" OR "Idiopathic Diffuse Interstitial Pulmonary Fibrosis" OR "Alveolitis, Extrinsic Allergic" OR "Allergic Alveolitis, Extrinsic" OR "Allergic Alveolitides, Extrinsic" OR "Alveolitides, Extrinsic Allergic" OR "Extrinsic Allergic Alveolitides" OR "Extrinsic Allergic Alveolitis" OR "Hypersensitivity Pneumonitis" OR "Hypersensitivity Pneumonitides" OR "Pneumonitides, Hypersensitivity" OR "Pneumonitis, Hypersensitivity" OR "Pneumoconiosis" OR "Pneumoconioses" OR "Bagassosis" OR "Sarcoidosis, Pulmonary" OR "Pulmonary Sarcoidosis" OR "Pulmonary Sarcoidoses" OR "Sarcoidoses, Pulmonary"):ti,ab,kw #6 #4 OR #5 #7 #3 AND #6 | 1,536 |
| PEDro June 11, 2026 | #1 Abstract & Title: "interstitial lung disease"; Method: clinical trial; When Searching: Match all search terms (AND) #2 Abstract & Title: "interstitial lung diseases"; Method: clinical trial; When Searching: Match all search terms (AND) #3 Abstract & Title: "idiopathic pulmonary fibrosis"; Method: clinical trial; When Searching: Match all search terms (AND) #4 Abstract & Title: pulmonary fibrosis; Method: clinical trial; When Searching: Match all search terms (AND) #5 Abstract & Title: fibrotic; Method: clinical trial; When Searching: Match all search terms (AND) #6 Abstract & Title: sarcoidosis; Method: clinical trial; When Searching: Match all search terms (AND) | 180 |

# Supplementary Appendix S2. Intervention Classification, Outcome Harmonization, and Data Transformation Methods

This appendix documents the final data-preparation framework used for 26 independent randomized controlled trials meeting the prespecified eligibility criteria. The audit workbook retained trial identifiers, linked publications, node assignments, arm-level analytical sample sizes, baseline and post-intervention data, change-score inputs, source pages, verbatim source text, conversion methods, and provenance flags. Only end-of-intervention data were used in the primary network meta-analyses. Analytical arm sizes may therefore differ from the numbers originally randomized when outcome data were unavailable.

## S2.1 Intervention classification framework

Interventions were classified according to explicitly prescribed active training components, irrespective of setting, supervision, or delivery format. Centre-based, home-based, inpatient, and telerehabilitation programmes were assigned to the same node when their core active training components were comparable. Programmes described as pulmonary rehabilitation were not assigned to a separate PR node.

| **Node code** | **Intervention category** | **Operational definition** | **Examples from included trials** |
| --- | --- | --- | --- |
| CON | Conventional control | Usual care, standard medical care, health education, waiting-list control, no additional structured exercise, or sham training without an active therapeutic load. Background exercise advice or other co-interventions were permitted when applied equally across all randomized groups. | Usual care; no exercise; sham IMT |
| AER | Aerobic training | Structured endurance or aerobic exercise without a substantial resistance or breathing-muscle component. | Walking, cycling, or active exergaming |
| AER_RES | Combined aerobic and resistance training | Programmes explicitly prescribing both aerobic/endurance and resistance/strengthening exercise. | Supervised or home/telerehabilitation aerobic plus strengthening exercise |
| BREATH_IMT | Breathing or inspiratory muscle training | Breathing exercises or inspiratory muscle training used as the principal active intervention. | Threshold IMT; structured respiratory exercise |
| AER_RES_BREATH | Combined aerobic, resistance, and breathing training | Multicomponent programmes containing aerobic exercise, resistance training, and breathing or inspiratory-muscle training. | Multicomponent pulmonary rehabilitation |
| MIND_BODY | Mind-body exercise | Structured movement integrating breathing and attentional or meditative elements. | Baduanjin, yoga, Pulmonary Daoyin |

*No eligible standalone resistance-training node remained. Same-node intervention arms within a multi-arm trial were pooled before network analysis; shared controls were retained once.*

## S2.2 Outcome harmonization, hierarchy, and direction of effect

The primary synthesis used end-of-intervention change scores. Directly reported change means and change SDs were prioritized. When unavailable, inputs were derived from baseline and post-intervention values, standard errors, confidence intervals, medians and interquartile ranges, digitized figures, or model-derived estimates. All 6MWD values were expressed in metres; FVC% predicted was expressed in percentage points and kept separate from absolute FVC in litres; standard SGRQ Total was kept separate from SGRQ-I and domain scores. For 6MWD and FVC% predicted, change was post-intervention minus baseline. SGRQ changes were multiplied by -1 before analysis so that positive values consistently indicated improvement.

## S2.3 Provenance of extracted change-score records before within-study pooling

| **Outcome** | Extracted arm records before same-node pooling | **Directly reported** | **Imputed from baseline/post** | **Median/IQR conversion** | **Digitized** | **Other conversion** | **Model-derived/adjusted** |
| --- | --- | --- | --- | --- | --- | --- | --- |
| Six-minute walk distance (6MWD) | 54 | 18 | 20 | 2 | 6 | 4 | 4 |
| Percent-predicted forced vital capacity (FVC% predicted) | 23 | 9 | 10 | 2 | 0 | 0 | 2 |
| St George's Respiratory Questionnaire (SGRQ) total score | 23 | 11 | 8 | 2 | 0 | 0 | 2 |

*Before same-node pooling, the extraction datasets contained 54 arm records for 6MWD and 23 arm records for each of FVC% predicted and SGRQ Total. After the two aerobic arms in the Sarıtaş Arslan trial were pooled within study, the final network datasets comprised 53, 22, and 22 analytical arms, respectively. Participant totals were unchanged by pooling.* *This multi-arm trial remained eligible because it included comparisons between the pooled AER node and CON; the within-node comparison between the two aerobic arms was not analyzed separately.*

## S2.4 Transformation rules and assumptions

| **Procedure** | **Formula or operation** | **Use and assumption** |
| --- | --- | --- |
| Change mean from baseline and post values | Mean change = mean post – mean baseline | Used when arm-level baseline and post means were available but change means were not reported. |
| Change SD from baseline and post SDs | SDchange = sqrt(SDbaseline² + SDpost² – 2r × SDbaseline × SDpost) | The primary calculation assumed r = 0.50; alternate r values were retained in the audit workbook where applicable. |
| SE or SEM to SD | SD = SE × sqrt(n) | Applied only when the dispersion measure was explicitly reported as SE/SEM. |
| 95% CI to SD | SE = (upper – lower)/(2 × 1.96); SD = SE × sqrt(n) | Applied to arm-level means or changes under a conventional two-sided 95% CI. |
| Median and IQR to mean and SD | Wan-type conversion using sample-size-dependent formulae | Direct paired-change distributions were prioritized over separate baseline/post conversions. |
| Unit conversion | 1 foot = 0.3048 m | Both mean and SD were multiplied by 0.3048. |
| Digitized figures | Values extracted from vector/high-resolution figures; error bars interpreted according to the report | Digitized records were flagged explicitly; SE or CI error bars were converted to SD. |
| Adjusted/model-derived values | Adjusted post estimate combined with reported baseline where no direct change was available | These records were flagged because adjusted and raw quantities were combined; change SD was imputed with r = 0.50 when required. |

*Calculations retained the available numerical precision in the audit workbook; displayed values below are rounded. No contrast-only estimate was entered as an additional arm-level observation.*

## S2.5 Arm-level transformation log: Six-minute walk distance (6MWD)

Positive change values indicate improvement.

| **Trial** | **Arm (node)** | **n** | **Source** | **Original data basis** | **Transformation / assumption** | **Change mean used** | **Change SD used** |
| --- | --- | --- | --- | --- | --- | --- | --- |
| Vainshelboim 2014 | regular medical treatment (CON) | 17 | p6 | change: Δ 6MWD -10.6 ± 35.4 vs 70.4 ± 77 | Directly reported change mean and change SD; no change-score reconstruction was required. | -10.600 | 35.40 |
| Vainshelboim 2014 | exercise training-based PR (AER_RES_BREATH) | 15 | p6 | change: Δ 6MWD -10.6 ± 35.4 vs 70.4 ± 77 | Directly reported change mean and change SD; no change-score reconstruction was required. | 70.400 | 77.00 |
| Yuen 2019 | video game control (CON) | 8 | p3 | change: 6MWD change -22 ± 56 vs -60 ± 111; post: 6MWD post 305 ± 108 vs 372 ± 125 | Directly reported change mean and change SD; no change-score reconstruction was required. | -60.000 | 111.00 |
| Yuen 2019 | Wii Fit home exercise (AER) | 9 | p3 | change: 6MWD change -22 ± 56 vs -60 ± 111; post: 6MWD post 305 ± 108 vs 372 ± 125 | Directly reported change mean and change SD; no change-score reconstruction was required. | -22.000 | 56.00 |
| Dale 2014 | usual care (CON) | 17 | p6 | change: 6MWD change 73 ± 29 vs 20 ± 32; post: 6MWD week 8 post 548 ± 72 vs 489 ± 83 | Directly reported change mean and change SD; no change-score reconstruction was required. | 20.000 | 32.00 |
| Dale 2014 | exercise training (AER) | 17 | p6 | change: 6MWD change 73 ± 29 vs 20 ± 32; post: 6MWD week 8 post 548 ± 72 vs 489 ± 83 | Directly reported change mean and change SD; no change-score reconstruction was required. | 73.000 | 29.00 |
| Atmaca 2025 | control (CON) | 14 | p6 | post: 6MWD post 425.25 ± 171.37 vs 346.14 ± 109.29 | The change mean was calculated as post minus baseline; the change SD was imputed from baseline and post-intervention SDs assuming r = 0.50. | -19.720 | 108.75 |
| Atmaca 2025 | Baduanjin exercise (MIND_BODY) | 14 | p6 | post: 6MWD post 425.25 ± 171.37 vs 346.14 ± 109.29 | The change mean was calculated as post minus baseline; the change SD was imputed from baseline and post-intervention SDs assuming r = 0.50. | 56.870 | 159.17 |
| Kadura 2025 | usual standard of care (CON) | 30 | Table E2 | 6MWT feet: MYP 1268.79(403.62) to 1195.37(383.21), change -69.92(143.71); Control 1311.07(405.74) to 1166.76(526.65), change -146.07(279.52) | The directly reported change mean and SD were converted from feet to metres by multiplying both by 0.3048. | -44.522 | 85.20 |
| Kadura 2025 | modified yoga program (MIND_BODY) | 30 | Table E2 | 6MWT feet: MYP 1268.79(403.62) to 1195.37(383.21), change -69.92(143.71); Control 1311.07(405.74) to 1166.76(526.65), change -146.07(279.52) | The directly reported change mean and SD were converted from feet to metres by multiplying both by 0.3048. | -21.312 | 43.80 |
| Shen 2021 | control (CON) | 29 | p6 | change: 12-month 6MWD change -27.272 ± 91.668 vs -82.297 ± 91.753 | Directly reported change mean and change SD; no change-score reconstruction was required. | -82.297 | 91.75 |
| Shen 2021 | RRPF/LHP rehabilitation (BREATH_IMT) | 30 | p6 | change: 12-month 6MWD change -27.272 ± 91.668 vs -82.297 ± 91.753 | Directly reported change mean and change SD; no change-score reconstruction was required. | -27.272 | 91.67 |
| Kataoka 2023 | usual care (CON) | 43 | p4 text/Table 1 | 12-week 6MWD changes: PR 18 m (95% CI 4 to 32); control -5 m (95% CI -19 to 9) | The directly reported change mean was used; the change SD was derived from the reported 95% CI using SD = SE × √n. | -5.000 | 46.84 |
| Kataoka 2023 | programmed pulmonary rehabilitation (AER_RES) | 45 | p4 text/Table 1 | 12-week 6MWD changes: PR 18 m (95% CI 4 to 32); control -5 m (95% CI -19 to 9) | The directly reported change mean was used; the change SD was derived from the reported 95% CI using SD = SE × √n. | 18.000 | 47.92 |
| Buschulte 2026 | control (CON) | 7 | p6 | post: 6MWD post 393.4 ± 54.1 vs 340.8 ± 64.8 | The change mean was calculated as post minus baseline; the change SD was imputed from baseline and post-intervention SDs assuming r = 0.50. | -44.200 | 57.27 |
| Buschulte 2026 | yoga (MIND_BODY) | 5 | p6 | post: 6MWD post 393.4 ± 54.1 vs 340.8 ± 64.8 | The change mean was calculated as post minus baseline; the change SD was imputed from baseline and post-intervention SDs assuming r = 0.50. | -18.400 | 82.04 |
| Zaki 2023 | PR (AER_RES) | 25 | p7 | change: 6MWD change 74.64 ± 21.00 vs 35.00 ± 49.97; post: 6MWD post 383.36 ± 40.32 vs 335.46 ± 55.65 | Directly reported change mean and change SD; no change-score reconstruction was required. | 35.000 | 49.97 |
| Zaki 2023 | PR + IMT (AER_RES_BREATH) | 26 | p7 | change: 6MWD change 74.64 ± 21.00 vs 35.00 ± 49.97; post: 6MWD post 383.36 ± 40.32 vs 335.46 ± 55.65 | Directly reported change mean and change SD; no change-score reconstruction was required. | 74.640 | 21.00 |
| El-Komy 2019 | control (CON) | 25 | p6 | post: 6MWD post 478.5 ± 54.1 vs 433.04 ± 56.1 | The change mean was calculated as post minus baseline; the change SD was imputed from baseline and post-intervention SDs assuming r = 0.50. | 9.040 | 56.50 |
| El-Komy 2019 | pulmonary rehabilitation (AER_RES_BREATH) | 25 | p6 | post: 6MWD post 478.5 ± 54.1 vs 433.04 ± 56.1 | The change mean was calculated as post minus baseline; the change SD was imputed from baseline and post-intervention SDs assuming r = 0.50. | 56.400 | 55.45 |
| Cerdán-de-las-Heras 2021 | control (CON) | 14 | p6 | post: 3-month 6MWD post 469.91 ± 115.63 vs 420.80 ± 70.16 | The change mean was calculated as post minus baseline; the change SD was imputed from baseline and post-intervention SDs assuming r = 0.50. | -25.200 | 67.12 |
| Cerdán-de-las-Heras 2021 | VAPA telerehabilitation (AER_RES) | 15 | p6 | post: 3-month 6MWD post 469.91 ± 115.63 vs 420.80 ± 70.16 | The change mean was calculated as post minus baseline; the change SD was imputed from baseline and post-intervention SDs assuming r = 0.50. | 8.410 | 115.32 |
| NISHIYAMA 2008 | control (CON) | 15 | p4 | post: 6MWD post 472 ± 130 vs 427 ± 84 | The change mean was calculated as post minus baseline; the change SD was imputed from baseline and post-intervention SDs assuming r = 0.50. | -4.000 | 129.01 |
| NISHIYAMA 2008 | pulmonary rehabilitation (AER_RES) | 13 | p4 | post: 6MWD post 472 ± 130 vs 427 ± 84 | The change mean was calculated as post minus baseline; the change SD was imputed from baseline and post-intervention SDs assuming r = 0.50. | 42.000 | 103.77 |
| Zhou 2021 | control (CON) | 31 | Table 1 and Table 2 | Table 2 6MWD T2-T0: Control 12.42(21.20), Exercise 32.16(35.45), PD 60.44(52.63) | Directly reported change mean and change SD; no change-score reconstruction was required. | 12.420 | 21.20 |
| Zhou 2021 | stationary cycle exercise (AER) | 31 | Table 1 and Table 2 | Table 2 6MWD T2-T0: Control 12.42(21.20), Exercise 32.16(35.45), PD 60.44(52.63) | Directly reported change mean and change SD; no change-score reconstruction was required. | 32.160 | 35.45 |
| Zhou 2021 | Pulmonary Daoyin (MIND_BODY) | 32 | Table 1 and Table 2 | Table 2 6MWD T2-T0: Control 12.42(21.20), Exercise 32.16(35.45), PD 60.44(52.63) | Directly reported change mean and change SD; no change-score reconstruction was required. | 60.440 | 52.63 |
| Karadallı 2016 | sham (CON) | 15 | p7 | post: 6MWT post 623.3 ± 80.2 vs 566.3 ± 61.0 | The change mean was calculated as post minus baseline; the change SD was imputed from baseline and post-intervention SDs assuming r = 0.50. | 12.100 | 60.41 |
| Karadallı 2016 | IMT (BREATH_IMT) | 15 | p7 | post: 6MWT post 623.3 ± 80.2 vs 566.3 ± 61.0 | The change mean was calculated as post minus baseline; the change SD was imputed from baseline and post-intervention SDs assuming r = 0.50. | 65.700 | 88.04 |
| Aktan 2024 | sham/no-resistance IMT (CON) | 14 | p4 | Table 1 baseline mean(SD): control 412.5(80.5), IMT 427.9(68.6); Table 2 adjusted post EMM±SE: control 423.7±6.0, IMT 460.3±6.0; SD_post=6.0*sqrt(14)=22.45; change_mean=post-baseline; change_sd via Follmann rho=0.5. | The change mean was reconstructed from the adjusted post-intervention estimated marginal mean and the raw baseline mean; adjusted SE was converted to SD and the change SD was imputed assuming r = 0.50. | 11.200 | 71.95 |
| Aktan 2024 | telerehabilitation-assisted IMT (BREATH_IMT) | 14 | p4 | Table 1 baseline mean(SD): control 412.5(80.5), IMT 427.9(68.6); Table 2 adjusted post EMM±SE: control 423.7±6.0, IMT 460.3±6.0; SD_post=6.0*sqrt(14)=22.45; change_mean=post-baseline; change_sd via Follmann rho=0.5. | The change mean was reconstructed from the adjusted post-intervention estimated marginal mean and the raw baseline mean; adjusted SE was converted to SD and the change SD was imputed assuming r = 0.50. | 32.400 | 60.58 |
| Jackson 2014 | control (CON) | 10 | Table 6 | Table 6 mean±SEM: Control 339.4(34.4) to 324.1(35.5); Rehab 360.6(16.5) to 354.4(37.2) | Baseline and post-intervention SEMs were converted to SDs; the change mean was calculated as post minus baseline and the change SD was imputed assuming r = 0.50. | -15.300 | 110.56 |
| Jackson 2014 | pulmonary rehabilitation (AER_RES) | 11 | Table 6 | Table 6 mean±SEM: Control 339.4(34.4) to 324.1(35.5); Rehab 360.6(16.5) to 354.4(37.2) | Baseline and post-intervention SEMs were converted to SDs; the change mean was calculated as post minus baseline and the change SD was imputed assuming r = 0.50. | -6.200 | 107.08 |
| Cerdan de las Heras 2022 | control (CON) | 15 | p8 | post: 3-month 6MWD post 567.3 ± 103.6 vs 541.5 ± 108.6 | The change mean was calculated as post minus baseline; the change SD was imputed from baseline and post-intervention SDs assuming r = 0.50. | 14.000 | 144.12 |
| Cerdan de las Heras 2022 | VAPA telerehabilitation (AER_RES) | 15 | p8 | post: 3-month 6MWD post 567.3 ± 103.6 vs 541.5 ± 108.6 | The change mean was calculated as post minus baseline; the change SD was imputed from baseline and post-intervention SDs assuming r = 0.50. | 68.600 | 112.16 |
| Wang 2021 | drug treatment control (CON) | 30 | p5 | post: 12-week 6MWD post 345.9 ± 76.5 vs 403.6 ± 100.3 | The change mean was calculated as post minus baseline; the change SD was imputed from baseline and post-intervention SDs assuming r = 0.50. | 12.300 | 84.81 |
| Wang 2021 | home-based pulmonary rehabilitation (AER_RES_BREATH) | 30 | p5 | post: 12-week 6MWD post 345.9 ± 76.5 vs 403.6 ± 100.3 | The change mean was calculated as post minus baseline; the change SD was imputed from baseline and post-intervention SDs assuming r = 0.50. | 55.100 | 103.32 |
| Perez-Bogerd 2018 | usual medical care (CON) | 30 | main Table 2 | fresh full-text re-extraction; calc_id=C09_6MWD_CON | The change mean was reconstructed from model-derived baseline and post-intervention estimates; the change SD was imputed assuming r = 0.50. | -23.000 | 57.00 |
| Perez-Bogerd 2018 | pulmonary rehabilitation (AER_RES) | 30 | main Table 2 | fresh full-text re-extraction; calc_id=C09_6MWD_PR | The change mean was reconstructed from model-derived baseline and post-intervention estimates; the change SD was imputed assuming r = 0.50. | 49.000 | 57.00 |
| Zou 2026 | routine treatment (CON) | 95 | 5 | fresh full-text re-extraction; calc_id=C16_6MWD_CON | Directly reported change mean and change SD; no change-score reconstruction was required. | -9.130 | 30.76 |
| Zou 2026 | individualized pulmonary rehabilitation (AER_RES_BREATH) | 103 | 5 | fresh full-text re-extraction; calc_id=C16_6MWD_PR | Directly reported change mean and change SD; no change-score reconstruction was required. | 37.630 | 28.51 |
| Dowman 2017 | usual care/telephone support (CON) | 64 | 5 | fresh full-text re-extraction; calc_id=C22_6MWD_CON | The change mean and SE were digitized from the figure; SE was converted to SD using SD = SE × √n. | 2.053 | 69.92 |
| Dowman 2017 | supervised aerobic and resistance training (AER_RES) | 71 | 5 | fresh full-text re-extraction; calc_id=C22_6MWD_EX | The change mean and SE were digitized from the figure; SE was converted to SD using SD = SE × √n. | 23.077 | 68.33 |
| Naz 2018 | usual care (CON) | 9 | 5 | fresh full-text re-extraction; calc_id=C23_6MWD_CON | The directly reported change-score median and IQR were converted to a mean and SD using the Wan method. | -23.000 | 67.35 |
| Naz 2018 | structured exercise (AER_RES_BREATH) | 9 | 5 | fresh full-text re-extraction; calc_id=C23_6MWD_EX | The directly reported change-score median and IQR were converted to a mean and SD using the Wan method. | 44.333 | 27.12 |
| Jarosch 2020 | usual care (CON) | 17 | 5 (baseline Table 1); 6 (Figure 2A) | fresh full-text re-extraction; calc_id=C28_6MWD_CON | Post-intervention values were digitized; the change mean was calculated as post minus baseline and the change SD was imputed assuming r = 0.50. | -4.327 | 110.68 |
| Jarosch 2020 | comprehensive inpatient pulmonary rehabilitation (AER_RES_BREATH) | 34 | 5 (baseline Table 1); 6 (Figure 2A) | fresh full-text re-extraction; calc_id=C28_6MWD_PR | Post-intervention values were digitized; the change mean was calculated as post minus baseline and the change SD was imputed assuming r = 0.50. | 52.380 | 132.39 |
| Holland 2008 | weekly telephone support (CON) | 27 | 3 | fresh full-text re-extraction; calc_id=C39_6MWD_CON | The change mean and 95% CI were digitized from the figure; the CI was converted to SE and then to SD. | -3.734 | 30.52 |
| Holland 2008 | supervised aerobic and resistance exercise (AER_RES) | 30 | 3 | fresh full-text re-extraction; calc_id=C39_6MWD_EX | The change mean and 95% CI were digitized from the figure; the CI was converted to SE and then to SD. | 31.036 | 51.08 |
| Vicenzi 2026 | sham inspiratory muscle training at 5% MIP (CON) | 12 | Vicenzi Table 1 and vector Figure 1b; Bellocchi Methods | Reported baseline 6MWD 476 (69) m; vector Figure 1b digitized raw mean approximately 477.24 at baseline and 464.17 after 5 weeks. | The post-intervention dispersion was digitized; the change mean was calculated as post minus baseline and the change SD was imputed assuming r = 0.50. | -13.070 | 66.70 |
| Vicenzi 2026 | high-intensity inspiratory muscle training (BREATH_IMT) | 12 | Vicenzi Table 1 and vector Figure 1b; Bellocchi Methods | Reported baseline 6MWD 480 (72) m; vector Figure 1b digitized raw mean approximately 482.02 at baseline and 539.00 after 5 weeks. | The post-intervention dispersion was digitized; the change mean was calculated as post minus baseline and the change SD was imputed assuming r = 0.50. | 56.980 | 78.90 |
| Arslan 2026 | control group with common background advice (CON) | 15 | Table 3, p7 | Table 3, p7 directly reports baseline, post-intervention and change mean ± SD for CG, TAE and AVG. | Directly reported change mean and change SD; no change-score reconstruction was required. | 33.130 | 47.32 |
| Arslan 2026 | active video game aerobic exercise (AER) | 15 | Table 3, p7 | Table 3, p7 directly reports baseline, post-intervention and change mean ± SD for CG, TAE and AVG. | Directly reported change mean and change SD; no change-score reconstruction was required. | 69.800 | 50.17 |
| Arslan 2026 | traditional aerobic exercise (AER) | 15 | Table 3, p7 | Table 3, p7 directly reports baseline, post-intervention and change mean ± SD for CG, TAE and AVG. | Directly reported change mean and change SD; no change-score reconstruction was required. | 32.070 | 52.85 |

## S2.6 Arm-level transformation log: Percent-predicted forced vital capacity (FVC% predicted)

Positive change values indicate improvement.

| **Trial** | **Arm (node)** | **n** | **Source** | **Original data basis** | **Transformation / assumption** | **Change mean used** | **Change SD used** |
| --- | --- | --- | --- | --- | --- | --- | --- |
| Vainshelboim 2014 | regular medical treatment (CON) | 17 | p6 | change: Δ FVC% predicted -3 ± 7.6 vs 3 ± 8.4 | Directly reported change mean and change SD; no change-score reconstruction was required. | -3.000 | 7.60 |
| Vainshelboim 2014 | exercise training-based PR (AER_RES_BREATH) | 15 | p6 | change: Δ FVC% predicted -3 ± 7.6 vs 3 ± 8.4 | Directly reported change mean and change SD; no change-score reconstruction was required. | 3.000 | 8.40 |
| Atmaca 2025 | control (CON) | 14 | p6 | post: FVC% predicted post 61.86 ± 11.87 vs 62.07 ± 16.49 | The change mean was calculated as post minus baseline; the change SD was imputed from baseline and post-intervention SDs assuming r = 0.50. | -1.860 | 16.08 |
| Atmaca 2025 | Baduanjin exercise (MIND_BODY) | 14 | p6 | post: FVC% predicted post 61.86 ± 11.87 vs 62.07 ± 16.49 | The change mean was calculated as post minus baseline; the change SD was imputed from baseline and post-intervention SDs assuming r = 0.50. | 1.720 | 11.31 |
| Kadura 2025 | usual standard of care (CON) | 30 | Table E2 | Table E2 reports mean(SD) at baseline, week12, and change | Directly reported change mean and change SD; no change-score reconstruction was required. | 0.600 | 19.06 |
| Kadura 2025 | modified yoga program (MIND_BODY) | 30 | Table E2 | Table E2 reports mean(SD) at baseline, week12, and change | Directly reported change mean and change SD; no change-score reconstruction was required. | -2.550 | 6.09 |
| Buschulte 2026 | control (CON) | 7 | p6 | post: FVC% predicted post 69.4 ± 24.9 vs 75.2 ± 15.7 | The change mean was calculated as post minus baseline; the change SD was imputed from baseline and post-intervention SDs assuming r = 0.50. | -3.600 | 17.65 |
| Buschulte 2026 | yoga (MIND_BODY) | 5 | p6 | post: FVC% predicted post 69.4 ± 24.9 vs 75.2 ± 15.7 | The change mean was calculated as post minus baseline; the change SD was imputed from baseline and post-intervention SDs assuming r = 0.50. | -2.100 | 23.20 |
| Zaki 2023 | PR (AER_RES) | 25 | p7 | change: FVC% predicted change 0.24 ± 0.59 vs 0.16 ± 0.48; post: FVC% predicted post 59.80 ± 18.69 vs 58.00 ± 19.22 | Directly reported change mean and change SD; no change-score reconstruction was required. | 0.160 | 0.48 |
| Zaki 2023 | PR + IMT (AER_RES_BREATH) | 26 | p7 | change: FVC% predicted change 0.24 ± 0.59 vs 0.16 ± 0.48; post: FVC% predicted post 59.80 ± 18.69 vs 58.00 ± 19.22 | Directly reported change mean and change SD; no change-score reconstruction was required. | 0.240 | 0.59 |
| El-Komy 2019 | control (CON) | 25 | p6 | post: FVC% predicted post 58.3 ± 1.7 vs 56.88 ± 1.51 | The change mean was calculated as post minus baseline; the change SD was imputed from baseline and post-intervention SDs assuming r = 0.50. | -0.220 | 1.80 |
| El-Komy 2019 | pulmonary rehabilitation (AER_RES_BREATH) | 25 | p6 | post: FVC% predicted post 58.3 ± 1.7 vs 56.88 ± 1.51 | The change mean was calculated as post minus baseline; the change SD was imputed from baseline and post-intervention SDs assuming r = 0.50. | 1.500 | 1.83 |
| Karadallı 2016 | sham (CON) | 15 | p6 | post: FVC post 102.8 ± 12.4 vs 103.6 ± 16.9 | The change mean was calculated as post minus baseline; the change SD was imputed from baseline and post-intervention SDs assuming r = 0.50. | 3.500 | 19.88 |
| Karadallı 2016 | IMT (BREATH_IMT) | 15 | p6 | post: FVC post 102.8 ± 12.4 vs 103.6 ± 16.9 | The change mean was calculated as post minus baseline; the change SD was imputed from baseline and post-intervention SDs assuming r = 0.50. | 2.500 | 11.89 |
| Wang 2021 | drug treatment control (CON) | 30 | p5 | post: 12-week FVC% predicted post 74.2 ± 9.9 vs 79.4 ± 8.8 | The change mean was calculated as post minus baseline; the change SD was imputed from baseline and post-intervention SDs assuming r = 0.50. | 1.000 | 11.90 |
| Wang 2021 | home-based pulmonary rehabilitation (AER_RES_BREATH) | 30 | p5 | post: 12-week FVC% predicted post 74.2 ± 9.9 vs 79.4 ± 8.8 | The change mean was calculated as post minus baseline; the change SD was imputed from baseline and post-intervention SDs assuming r = 0.50. | 6.600 | 8.56 |
| Perez-Bogerd 2018 | usual medical care (CON) | 30 | supplement Table S5 | fresh full-text re-extraction; calc_id=C09_FVC_pct_CON | The change mean was reconstructed from model-derived baseline and post-intervention estimates; the change SD was imputed assuming r = 0.50. | -2.000 | 22.00 |
| Perez-Bogerd 2018 | pulmonary rehabilitation (AER_RES) | 30 | supplement Table S5 | fresh full-text re-extraction; calc_id=C09_FVC_pct_PR | The change mean was reconstructed from model-derived baseline and post-intervention estimates; the change SD was imputed assuming r = 0.50. | 3.000 | 22.00 |
| Naz 2018 | usual care (CON) | 9 | 5 | fresh full-text re-extraction; calc_id=C23_FVC_pct_CON | The directly reported change-score median and IQR were converted to a mean and SD using the Wan method. | -1.333 | 11.37 |
| Naz 2018 | structured exercise (AER_RES_BREATH) | 9 | 5 | fresh full-text re-extraction; calc_id=C23_FVC_pct_EX | The directly reported change-score median and IQR were converted to a mean and SD using the Wan method. | -0.333 | 10.50 |
| Arslan 2026 | control group with common background advice (CON) | 15 | Table 4, p8 | Table 4, p8 directly reports baseline, post-intervention and change mean ± SD for CG, TAE and AVG. | Directly reported change mean and change SD; no change-score reconstruction was required. | 0.210 | 6.75 |
| Arslan 2026 | active video game aerobic exercise (AER) | 15 | Table 4, p8 | Table 4, p8 directly reports baseline, post-intervention and change mean ± SD for CG, TAE and AVG. | Directly reported change mean and change SD; no change-score reconstruction was required. | -0.330 | 4.97 |
| Arslan 2026 | traditional aerobic exercise (AER) | 15 | Table 4, p8 | Table 4, p8 directly reports baseline, post-intervention and change mean ± SD for CG, TAE and AVG. | Directly reported change mean and change SD; no change-score reconstruction was required. | -0.050 | 7.27 |

## S2.7 Arm-level transformation log: St George's Respiratory Questionnaire (SGRQ) total score

Displayed change means are the benefit-oriented values entered into the NMA after multiplying the original SGRQ change by -1; positive values indicate improvement.

| **Trial** | **Arm (node)** | **n** | **Source** | **Original data basis** | **Transformation / assumption** | **Change mean used** | **Change SD used** |
| --- | --- | --- | --- | --- | --- | --- | --- |
| Vainshelboim 2014 | regular medical treatment (CON) | 17 | p6 | change: SGRQ total change 2.8 ± 3.6 vs -6.9 ± 6.5 | Directly reported change mean and change SD; no change-score reconstruction was required. The SGRQ change was multiplied by −1 so that positive values indicated benefit. | -2.800 | 3.60 |
| Vainshelboim 2014 | exercise training-based PR (AER_RES_BREATH) | 15 | p6 | change: SGRQ total change 2.8 ± 3.6 vs -6.9 ± 6.5 | Directly reported change mean and change SD; no change-score reconstruction was required. The SGRQ change was multiplied by −1 so that positive values indicated benefit. | 6.900 | 6.50 |
| Yuen 2019 | video game control (CON) | 10 | p3 | change: SGRQ total change 3 ± 9 vs 1 ± 11; post: SGRQ total post 47 ± 15 vs 33 ± 17 | Directly reported change mean and change SD; no change-score reconstruction was required. The SGRQ change was multiplied by −1 so that positive values indicated benefit. | -1.000 | 11.00 |
| Yuen 2019 | Wii Fit home exercise (AER) | 10 | p3 | change: SGRQ total change 3 ± 9 vs 1 ± 11; post: SGRQ total post 47 ± 15 vs 33 ± 17 | Directly reported change mean and change SD; no change-score reconstruction was required. The SGRQ change was multiplied by −1 so that positive values indicated benefit. | -3.000 | 9.00 |
| Atmaca 2025 | control (CON) | 14 | p6 | post: SGRQ total post 36.50 ± 20.90 vs 54.93 ± 10.17 | The change mean was calculated as post minus baseline; the change SD was imputed from baseline and post-intervention SDs assuming r = 0.50. The SGRQ change was multiplied by −1 so that positive values indicated benefit. | -2.070 | 12.11 |
| Atmaca 2025 | Baduanjin exercise (MIND_BODY) | 14 | p6 | post: SGRQ total post 36.50 ± 20.90 vs 54.93 ± 10.17 | The change mean was calculated as post minus baseline; the change SD was imputed from baseline and post-intervention SDs assuming r = 0.50. The SGRQ change was multiplied by −1 so that positive values indicated benefit. | 13.130 | 20.64 |
| Shen 2021 | control (CON) | 29 | p6 | change: 12-month SGRQ change -3.723 ± 10.350 vs 4.806 ± 10.359 | Directly reported change mean and change SD; no change-score reconstruction was required. The SGRQ change was multiplied by −1 so that positive values indicated benefit. | -4.806 | 10.36 |
| Shen 2021 | RRPF/LHP rehabilitation (BREATH_IMT) | 30 | p6 | change: 12-month SGRQ change -3.723 ± 10.350 vs 4.806 ± 10.359 | Directly reported change mean and change SD; no change-score reconstruction was required. The SGRQ change was multiplied by −1 so that positive values indicated benefit. | 3.723 | 10.35 |
| Zaki 2023 | PR (AER_RES) | 25 | p8 | change: SGRQ-T change -13.16 ± 7.22 vs -10.42 ± 11.7; post: SGRQ-T post 23.03 ± 6.36 vs 27.04 ± 7.04 | Directly reported change mean and change SD; no change-score reconstruction was required. The SGRQ change was multiplied by −1 so that positive values indicated benefit. | 10.420 | 11.70 |
| Zaki 2023 | PR + IMT (AER_RES_BREATH) | 26 | p8 | change: SGRQ-T change -13.16 ± 7.22 vs -10.42 ± 11.7; post: SGRQ-T post 23.03 ± 6.36 vs 27.04 ± 7.04 | Directly reported change mean and change SD; no change-score reconstruction was required. The SGRQ change was multiplied by −1 so that positive values indicated benefit. | 13.160 | 7.22 |
| NISHIYAMA 2008 | control (CON) | 15 | p4 | post: SGRQ total post 40.9 ± 20.7 vs 47.3 ± 17.4 | The change mean was calculated as post minus baseline; the change SD was imputed from baseline and post-intervention SDs assuming r = 0.50. The SGRQ change was multiplied by −1 so that positive values indicated benefit. | -3.100 | 21.77 |
| NISHIYAMA 2008 | pulmonary rehabilitation (AER_RES) | 13 | p4 | post: SGRQ total post 40.9 ± 20.7 vs 47.3 ± 17.4 | The change mean was calculated as post minus baseline; the change SD was imputed from baseline and post-intervention SDs assuming r = 0.50. The SGRQ change was multiplied by −1 so that positive values indicated benefit. | 2.900 | 16.88 |
| Karadallı 2016 | sham (CON) | 15 | p8 | post: SGRQ total post 29.2 ± 20.3 vs 31.7 ± 17.1 | The change mean was calculated as post minus baseline; the change SD was imputed from baseline and post-intervention SDs assuming r = 0.50. The SGRQ change was multiplied by −1 so that positive values indicated benefit. | 9.500 | 16.86 |
| Karadallı 2016 | IMT (BREATH_IMT) | 15 | p8 | post: SGRQ total post 29.2 ± 20.3 vs 31.7 ± 17.1 | The change mean was calculated as post minus baseline; the change SD was imputed from baseline and post-intervention SDs assuming r = 0.50. The SGRQ change was multiplied by −1 so that positive values indicated benefit. | 11.100 | 20.66 |
| Wang 2021 | drug treatment control (CON) | 30 | p5 | post: 12-week SGRQ total post 45.2 ± 15.9 vs 30.8 ± 16.7 | The change mean was calculated as post minus baseline; the change SD was imputed from baseline and post-intervention SDs assuming r = 0.50. The SGRQ change was multiplied by −1 so that positive values indicated benefit. | 0.900 | 17.47 |
| Wang 2021 | home-based pulmonary rehabilitation (AER_RES_BREATH) | 30 | p5 | post: 12-week SGRQ total post 45.2 ± 15.9 vs 30.8 ± 16.7 | The change mean was calculated as post minus baseline; the change SD was imputed from baseline and post-intervention SDs assuming r = 0.50. The SGRQ change was multiplied by −1 so that positive values indicated benefit. | 17.300 | 18.76 |
| Perez-Bogerd 2018 | usual medical care (CON) | 30 | main Table 2 | fresh full-text re-extraction; calc_id=C09_SGRQ_Total_CON | The change mean was reconstructed from model-derived baseline and post-intervention estimates; the change SD was imputed assuming r = 0.50. The SGRQ change was multiplied by −1 so that positive values indicated benefit. | -6.000 | 14.00 |
| Perez-Bogerd 2018 | pulmonary rehabilitation (AER_RES) | 30 | main Table 2 | fresh full-text re-extraction; calc_id=C09_SGRQ_Total_PR | The change mean was reconstructed from model-derived baseline and post-intervention estimates; the change SD was imputed assuming r = 0.50. The SGRQ change was multiplied by −1 so that positive values indicated benefit. | 7.000 | 14.00 |
| Naz 2018 | usual care (CON) | 9 | 6 | fresh full-text re-extraction; calc_id=C23_SGRQ_Total_CON | The directly reported change-score median and IQR were converted to a mean and SD using the Wan method. The SGRQ change was multiplied by −1 so that positive values indicated benefit. | 7.000 | 12.25 |
| Naz 2018 | structured exercise (AER_RES_BREATH) | 9 | 6 | fresh full-text re-extraction; calc_id=C23_SGRQ_Total_EX | The directly reported change-score median and IQR were converted to a mean and SD using the Wan method. The SGRQ change was multiplied by −1 so that positive values indicated benefit. | 14.333 | 22.74 |
| Arslan 2026 | control group with common background advice (CON) | 15 | Table 5, p9 | Table 5, p9 directly reports baseline, post-intervention and change mean ± SD for CG, TAE and AVG. | Directly reported change mean and change SD; no change-score reconstruction was required. The SGRQ change was multiplied by −1 so that positive values indicated benefit. | 0.230 | 7.22 |
| Arslan 2026 | active video game aerobic exercise (AER) | 15 | Table 5, p9 | Table 5, p9 directly reports baseline, post-intervention and change mean ± SD for CG, TAE and AVG. | Directly reported change mean and change SD; no change-score reconstruction was required. The SGRQ change was multiplied by −1 so that positive values indicated benefit. | 16.920 | 17.26 |
| Arslan 2026 | traditional aerobic exercise (AER) | 15 | Table 5, p9 | Table 5, p9 directly reports baseline, post-intervention and change mean ± SD for CG, TAE and AVG. | Directly reported change mean and change SD; no change-score reconstruction was required. The SGRQ change was multiplied by −1 so that positive values indicated benefit. | 10.290 | 15.58 |

## S2.8 Quality-control and reproducibility checks

• Outcome units were isolated before analysis: 6MWD in metres, FVC% predicted in percentage points, and SGRQ Total in points.

• Absolute FVC in litres, SGRQ-I, and SGRQ domain scores were not pooled with the prespecified outcomes.

• All final analytical arm records had non-missing sample size, change mean, and change SD; n > 1 and SD > 0 were verified.

• Duplicate or companion publications were merged at the independent-trial level; shared controls were retained once; same-node arms were pooled before NMA.

• The final input files passed study-by-treatment uniqueness checks and trial-level consistency checks for subgroup covariates.

• Source pages, verbatim source text, provenance flags, conversion formulae, and protocol-boundary notes were retained in the final extraction workbook.

*The complete extraction and transformation workbook is available from the corresponding author upon reasonable request.*

# Supplementary Table S1. Treatment-Node Definitions and Network Composition

| **Outcome** | **Network letter** | **Treatment code** | **Treatment-node definition** | **Arms** | **Participants** |
| --- | --- | --- | --- | --- | --- |
| 6MWD | A | CON | Usual care, standard medical care, health education, waiting-list control, no additional structured exercise, or sham training without an active therapeutic load; common background exercise advice was permitted when applied equally across randomized groups. | 25 | 603 |
| 6MWD | B | AER | Structured endurance or aerobic exercise without a substantial resistance or breathing-muscle component. | 4 | 87 |
| 6MWD | C | AER_RES | Programmes explicitly prescribing both aerobic/endurance and resistance/strengthening exercise. | 9 | 255 |
| 6MWD | D | BREATH_IMT | Breathing exercises or inspiratory muscle training used as the principal active intervention. | 4 | 71 |
| 6MWD | E | AER_RES_BREATH | Multicomponent programmes containing aerobic exercise, resistance training, and breathing or inspiratory-muscle training. | 7 | 242 |
| 6MWD | F | MIND_BODY | Structured movement integrating breathing and attentional or meditative elements. | 4 | 81 |
| FVC% predicted | A | CON | Usual care, standard medical care, health education, waiting-list control, no additional structured exercise, or sham training without an active therapeutic load; common background exercise advice was permitted when applied equally across randomized groups. | 10 | 192 |
| FVC% predicted | B | AER | Structured endurance or aerobic exercise without a substantial resistance or breathing-muscle component. | 1 | 30 |
| FVC% predicted | C | AER_RES | Programmes explicitly prescribing both aerobic/endurance and resistance/strengthening exercise. | 2 | 55 |
| FVC% predicted | D | BREATH_IMT | Breathing exercises or inspiratory muscle training used as the principal active intervention. | 1 | 15 |
| FVC% predicted | E | AER_RES_BREATH | Multicomponent programmes containing aerobic exercise, resistance training, and breathing or inspiratory-muscle training. | 5 | 105 |
| FVC% predicted | F | MIND_BODY | Structured movement integrating breathing and attentional or meditative elements. | 3 | 49 |
| SGRQ Total | A | CON | Usual care, standard medical care, health education, waiting-list control, no additional structured exercise, or sham training without an active therapeutic load; common background exercise advice was permitted when applied equally across randomized groups. | 10 | 184 |
| SGRQ Total | B | AER | Structured endurance or aerobic exercise without a substantial resistance or breathing-muscle component. | 2 | 40 |
| SGRQ Total | C | AER_RES | Programmes explicitly prescribing both aerobic/endurance and resistance/strengthening exercise. | 3 | 68 |
| SGRQ Total | D | BREATH_IMT | Breathing exercises or inspiratory muscle training used as the principal active intervention. | 2 | 45 |
| SGRQ Total | E | AER_RES_BREATH | Multicomponent programmes containing aerobic exercise, resistance training, and breathing or inspiratory-muscle training. | 4 | 80 |
| SGRQ Total | F | MIND_BODY | Structured movement integrating breathing and attentional or meditative elements. | 1 | 14 |

*Arm counts refer to analytical arms after within-study pooling of same-node intervention arms. Interventions were classified by active training components, irrespective of delivery setting or supervision format.*

# Supplementary Table S2. Sensitivity Analyses Using Alternative Effect Models and Exclusion of Non-Directly Reported Change Data

## Table S2A. Six-minute walk distance

| **Treatment vs CON** | **Primary random-effects MD (95% CI)** | **Fixed-effect MD (95% CI)** | **Random-effects SMD (95% CI)** | **Direction consistent across models** | **Significance retained** |
| --- | --- | --- | --- | --- | --- |
| AER | 29.46 (11.82, 47.10) | 28.77 (17.82, 39.71) | 0.58 (0.11, 1.05) | Yes | Yes |
| AER_RES | 31.94 (17.61, 46.27) | 28.83 (18.97, 38.69) | 0.47 (0.17, 0.78) | Yes | Yes |
| BREATH_IMT | 48.59 (20.18, 77.01) | 48.36 (22.57, 74.15) | 0.62 (0.13, 1.10) | Yes | Yes |
| AER_RES_BREATH | 57.12 (41.76, 72.48) | 50.74 (43.49, 57.99) | 1.05 (0.71, 1.40) | Yes | Yes |
| MIND_BODY | 43.48 (20.91, 66.05) | 44.01 (27.59, 60.42) | 0.74 (0.27, 1.21) | Yes | Yes |

*Positive estimates indicate benefit. “Direction consistent across models” indicates that the point estimates had the same sign under all three analyses. “Significance retained” requires all three 95% confidence intervals to exclude the null value in the same direction.*

## Table S2B. Percent-predicted forced vital capacity

| **Treatment vs CON** | **Primary random-effects MD (95% CI)** | **Fixed-effect MD (95% CI)** | **Random-effects SMD (95% CI)** | **Direction consistent across models** | **Significance retained** |
| --- | --- | --- | --- | --- | --- |
| AER | -0.40 (-5.47, 4.67) | -0.40 (-4.46, 3.66) | -0.06 (-0.68, 0.56) | Yes | No |
| AER_RES | 3.12 (-1.12, 7.36) | 1.92 (0.91, 2.92) | 0.33 (-0.07, 0.73) | Yes | No |
| BREATH_IMT | -1.00 (-13.11, 11.11) | -1.00 (-12.72, 10.72) | -0.06 (-0.78, 0.66) | Yes | No |
| AER_RES_BREATH | 3.08 (0.05, 6.12) | 2.00 (1.03, 2.96) | 0.60 (0.30, 0.89) | Yes | Yes |
| MIND_BODY | -0.69 (-6.80, 5.42) | -0.82 (-6.53, 4.89) | -0.05 (-0.45, 0.34) | Yes | No |

*Positive estimates indicate benefit. “Direction consistent across models” indicates that the point estimates had the same sign under all three analyses. “Significance retained” requires all three 95% confidence intervals to exclude the null value in the same direction.*

## Table S2C. St George's Respiratory Questionnaire total score

| **Treatment vs CON** | **Primary random-effects MD (95% CI)** | **Fixed-effect MD (95% CI)** | **Random-effects SMD (95% CI)** | **Direction consistent across models** | **Significance retained** |
| --- | --- | --- | --- | --- | --- |
| AER | 6.55 (-1.57, 14.66) | 7.48 (2.03, 12.94) | 0.45 (-0.34, 1.23) | Yes | No |
| AER_RES | 10.08 (2.83, 17.33) | 9.69 (5.21, 14.18) | 0.68 (0.06, 1.29) | Yes | Yes |
| BREATH_IMT | 6.60 (-1.85, 15.06) | 7.61 (2.69, 12.53) | 0.49 (-0.23, 1.21) | Yes | No |
| AER_RES_BREATH | 11.88 (5.80, 17.95) | 11.05 (7.93, 14.17) | 1.01 (0.45, 1.57) | Yes | Yes |
| MIND_BODY | 15.20 (0.18, 30.22) | 15.20 (2.66, 27.74) | 0.87 (-0.25, 1.99) | Yes | No |

*Positive estimates indicate benefit. “Direction consistent across models” indicates that the point estimates had the same sign under all three analyses. “Significance retained” requires all three 95% confidence intervals to exclude the null value in the same direction.*

## Table S2D. Analysis Restricted to Directly Reported Change Means and Change SDs

**Table S2D-1. Network Characteristics and Model Specification**

| **Outcome** | **Dataset** | **Studies retained** | **Arms** | **Participants** | **Nodes** | **Effect model** | **Tau** | **Heterogeneity Q (df), P** | **Global inconsistency** |
| --- | --- | --- | --- | --- | --- | --- | --- | --- | --- |
| 6MWD | Primary | 26 | 53 | 1,339 | 6 | Random-effects REML | 12.02 | 28.07 (23), P = 0.213 | χ² = 3.80 (3), P = 0.284 |
| 6MWD | Strict | 8 | 17 | 530 | 6 | Random-effects REML | 16.27 | 9.76 (5), P = 0.082 | χ² = 0.56 (1), P = 0.455 |
| FVC% predicted | Primary | 11 | 22 | 446 | 6 | Random-effects REML | 1.55 | 5.57 (7), P = 0.591 | χ² = 0.06 (1), P = 0.815 |
| FVC% predicted | Strict | 4 | 8 | 188 | 5 | Fixed-effect | Not estimated | 0.12 (1), P = 0.729 | Not estimable |
| SGRQ Total | Primary | 11 | 22 | 431 | 6 | Random-effects REML | 4.22 | 11.45 (7), P = 0.120 | χ² = 0.05 (1), P = 0.823 |
| SGRQ Total | Strict | 5 | 10 | 207 | 5 | Random-effects REML | 10.07 | 7.22 (2), P = 0.027 | Not estimable |

**Table S2D-2. Treatment Effects versus Control in the Primary and Strict Analyses**

| **Outcome** | **Treatment vs CON** | **Primary MD (95% CI)** | **Strict MD (95% CI)** | **Strict P** | **Direction consistent** | **Strict 95% CI excludes 0** |
| --- | --- | --- | --- | --- | --- | --- |
| 6MWD | AER | 29.46 (11.82, 47.10) | 31.12 (9.50, 52.75) | 0.005 | Yes | Yes |
| 6MWD | AER_RES | 31.94 (17.61, 46.27) | 16.63 (-31.68, 64.94) | 0.500 | Yes | No |
| 6MWD | BREATH_IMT | 48.59 (20.18, 77.01) | 55.02 (-1.62, 111.67) | 0.057 | Yes | No |
| 6MWD | AER_RES_BREATH | 57.12 (41.76, 72.48) | 56.28 (26.81, 85.74) | <0.001 | Yes | Yes |
| 6MWD | MIND_BODY | 43.48 (20.91, 66.05) | 53.25 (17.95, 88.55) | 0.003 | Yes | Yes |
| FVC% predicted | AER | -0.40 (-5.47, 4.67) | -0.40 (-4.46, 3.66) | 0.847 | Yes | No |
| FVC% predicted | AER_RES | 3.12 (-1.12, 7.36) | 5.76 (0.25, 11.27) | 0.041 | Yes | Yes |
| FVC% predicted | BREATH_IMT | -1.00 (-13.11, 11.11) | Not estimable | Not estimable | Not estimable | Not estimable |
| FVC% predicted | AER_RES_BREATH | 3.08 (0.05, 6.12) | 5.84 (0.33, 11.34) | 0.038 | Yes | Yes |
| FVC% predicted | MIND_BODY | -0.69 (-6.80, 5.42) | -3.15 (-10.31, 4.01) | 0.389 | Yes | No |
| SGRQ Total | AER | 6.55 (-1.57, 14.66) | 5.94 (-9.12, 20.99) | 0.440 | Yes | No |
| SGRQ Total | AER_RES | 10.08 (2.83, 17.33) | 6.95 (-21.71, 35.60) | 0.635 | Yes | No |
| SGRQ Total | BREATH_IMT | 6.60 (-1.85, 15.06) | 8.53 (-11.91, 28.97) | 0.413 | Yes | No |
| SGRQ Total | AER_RES_BREATH | 11.88 (5.80, 17.95) | 9.69 (-10.39, 29.77) | 0.344 | Yes | No |
| SGRQ Total | MIND_BODY | 15.20 (0.18, 30.22) | Not estimable | Not estimable | Not estimable | Not estimable |

*A trial-outcome dataset was retained in the strict analysis only when every included arm directly reported both the change mean and change SD. The entire trial was excluded for that outcome if any arm required imputation, statistical conversion, digitization, or model-derived reconstruction. FVC% predicted used a fixed-effect consistency model because between-study variance was not identifiable in the four-study strict network; consequently, τ was not estimated. “Not estimable” indicates that the corresponding treatment node was absent from the strict network.*

# Supplementary Table S3. Leave-One-Out Sensitivity Analyses for the Three Primary Outcomes

## Six-minute walk distance

| **Treatment vs CON** | **Full-model MD (95% CI)** | **Range of leave-one-out estimates** | **Extreme 95% CI limits** | **Successful analyses** | **All estimates positive** | **All CIs excluded 0** |
| --- | --- | --- | --- | --- | --- | --- |
| AER | 29.46 (11.82, 47.10) | 18.53 to 39.24 | 0.07 to 62.29 | 26/26 | Yes | Yes |
| AER_RES | 31.94 (17.61, 46.27) | 24.40 to 35.29 | 11.37 to 51.10 | 26/26 | Yes | Yes |
| AER_RES_BREATH | 57.12 (41.76, 72.48) | 52.93 to 63.10 | 36.22 to 81.97 | 26/26 | Yes | Yes |
| BREATH_IMT | 48.59 (20.18, 77.01) | 43.12 to 58.76 | 11.23 to 91.98 | 26/26 | Yes | Yes |
| MIND_BODY | 43.48 (20.91, 66.05) | 29.67 to 51.90 | -5.19 to 78.55 | 26/26 | Yes | No |

## Percent-predicted forced vital capacity

| **Treatment vs CON** | **Full-model MD (95% CI)** | **Range of leave-one-out estimates** | **Extreme 95% CI limits** | **Successful analyses** | **All estimates positive** | **All CIs excluded 0** |
| --- | --- | --- | --- | --- | --- | --- |
| AER | -0.40 (-5.47, 4.67) | -0.40 to -0.40 | -5.75 to 4.95 | 10/11 | No | No |
| AER_RES | 3.12 (-1.12, 7.36) | 2.31 to 5.05 | -6.62 to 16.62 | 11/11 | Yes | No |
| AER_RES_BREATH | 3.08 (0.05, 6.12) | 2.30 to 5.13 | -1.00 to 8.51 | 11/11 | Yes | No |
| BREATH_IMT | -1.00 (-13.11, 11.11) | -1.00 to -1.00 | -13.23 to 11.23 | 10/11 | No | No |
| MIND_BODY | -0.69 (-6.80, 5.42) | -2.72 to 3.24 | -10.13 to 13.06 | 11/11 | No | No |

## SGRQ total score

| **Treatment vs CON** | **Full-model MD (95% CI)** | **Range of leave-one-out estimates** | **Extreme 95% CI limits** | **Successful analyses** | **All estimates positive** | **All CIs excluded 0** |
| --- | --- | --- | --- | --- | --- | --- |
| AER | 6.55 (-1.57, 14.66) | -2.00 to 13.37 | -11.13 to 20.72 | 11/11 | No | No |
| AER_RES | 10.08 (2.83, 17.33) | 7.73 to 11.08 | -2.61 to 20.76 | 11/11 | Yes | No |
| AER_RES_BREATH | 11.88 (5.80, 17.95) | 10.40 to 13.52 | 3.01 to 22.30 | 11/11 | Yes | Yes |
| BREATH_IMT | 6.60 (-1.85, 15.06) | 1.60 to 8.53 | -14.54 to 18.85 | 11/11 | Yes | No |
| MIND_BODY | 15.20 (0.18, 30.22) | 15.20 to 15.20 | -0.79 to 31.19 | 10/11 | Yes | No |

Note: A successful leave-one-out analysis could still omit a treatment node when the excluded trial was the only study supporting that node. Thus, 10/11 does not indicate model failure; it indicates that the corresponding effect was not estimable in one reduced network.

# Supplementary Table S4. Subgroup Analyses and Network Meta-Regression Results

## Table S4A. Subgroup network characteristics

| **Outcome** | **Subgroup factor** | **Level** | **Studies** | **Arms** | **Participants** | **Nodes** |
| --- | --- | --- | --- | --- | --- | --- |
| 6MWD | Intervention setting | Centre/hospital-based | 17 | 35 | 998 | 6 |
| 6MWD | Intervention setting | Home-based/telerehabilitation | 9 | 18 | 341 | 6 |
| 6MWD | Intervention duration | <8 weeks | 4 | 9 | 199 | 5 |
| 6MWD | Intervention duration | 8–12 weeks | 17 | 34 | 764 | 6 |
| 6MWD | Intervention duration | >12 weeks | 5 | 10 | 376 | 4 |
| 6MWD | Disease type | IPF | 12 | 25 | 535 | 6 |
| 6MWD | Disease type | Other or mixed ILD | 14 | 28 | 804 | 6 |
| 6MWD | Supervision level | Higher supervision | 16 | 33 | 910 | 6 |
| 6MWD | Supervision level | Lower or remote supervision | 10 | 20 | 429 | 6 |
| FVC% predicted | Intervention setting | Centre/hospital-based | 7 | 14 | 268 | 5 |
| FVC% predicted | Intervention setting | Home-based/telerehabilitation | 4 | 8 | 178 | 4 |
| FVC% predicted | Intervention duration | <8 weeks | 1 | 2 | 30 | 2 |
| FVC% predicted | Intervention duration | 8–12 weeks | 9 | 18 | 356 | 5 |
| FVC% predicted | Intervention duration | >12 weeks | 1 | 2 | 60 | 2 |
| FVC% predicted | Disease type | IPF | 3 | 6 | 120 | 3 |
| FVC% predicted | Disease type | Other or mixed ILD | 8 | 16 | 326 | 6 |
| FVC% predicted | Supervision level | Higher supervision | 7 | 14 | 268 | 5 |
| FVC% predicted | Supervision level | Lower or remote supervision | 4 | 8 | 178 | 4 |
| SGRQ Total | Intervention setting | Centre/hospital-based | 6 | 12 | 234 | 4 |
| SGRQ Total | Intervention setting | Home-based/telerehabilitation | 5 | 10 | 197 | 5 |
| SGRQ Total | Intervention duration | <8 weeks | 1 | 2 | 30 | 2 |
| SGRQ Total | Intervention duration | 8–12 weeks | 8 | 16 | 282 | 5 |
| SGRQ Total | Intervention duration | >12 weeks | 2 | 4 | 119 | 3 |
| SGRQ Total | Disease type | IPF | 5 | 10 | 167 | 6 |
| SGRQ Total | Disease type | Other or mixed ILD | 6 | 12 | 264 | 5 |
| SGRQ Total | Supervision level | Higher supervision | 6 | 12 | 234 | 4 |
| SGRQ Total | Supervision level | Lower or remote supervision | 5 | 10 | 197 | 5 |

## Table S4B. Subgroup-specific treatment effects versus CON

### 6MWD

| **Factor** | **Level** | **Treatment vs CON** | **MD (95% CI)** | **P** | **Tau** | **Studies in subgroup network** |
| --- | --- | --- | --- | --- | --- | --- |
| Intervention setting | Centre/hospital-based | AER | 30.17 (10.47, 49.87) | 0.003 | 13.67 | 17 |
| Intervention setting | Centre/hospital-based | AER_RES | 31.92 (16.12, 47.72) | <0.001 | 13.67 | 17 |
| Intervention setting | Centre/hospital-based | AER_RES_BREATH | 58.83 (41.50, 76.17) | <0.001 | 13.67 | 17 |
| Intervention setting | Centre/hospital-based | BREATH_IMT | 70.05 (5.75, 134.35) | 0.033 | 13.67 | 17 |
| Intervention setting | Centre/hospital-based | MIND_BODY | 49.80 (20.03, 79.57) | 0.001 | 13.67 | 17 |
| Intervention setting | Home-based/telerehabilitation | AER | 38.00 (-47.18, 123.18) | 0.382 | <0.001 | 9 |
| Intervention setting | Home-based/telerehabilitation | AER_RES | 41.00 (-13.84, 95.84) | 0.143 | <0.001 | 9 |
| Intervention setting | Home-based/telerehabilitation | AER_RES_BREATH | 42.80 (-5.03, 90.63) | 0.079 | <0.001 | 9 |
| Intervention setting | Home-based/telerehabilitation | BREATH_IMT | 43.11 (14.38, 71.85) | 0.003 | <0.001 | 9 |
| Intervention setting | Home-based/telerehabilitation | MIND_BODY | 28.73 (-3.74, 61.19) | 0.083 | <0.001 | 9 |
| Intervention duration | <8 weeks | AER | 19.74 (5.20, 34.28) | 0.008 | <0.001 | 4 |
| Intervention duration | <8 weeks | AER_RES_BREATH | 56.71 (-12.20, 125.62) | 0.107 | <0.001 | 4 |
| Intervention duration | <8 weeks | BREATH_IMT | 61.18 (21.50, 100.86) | 0.003 | <0.001 | 4 |
| Intervention duration | <8 weeks | MIND_BODY | 48.02 (28.32, 67.72) | <0.001 | <0.001 | 4 |
| Intervention duration | 8–12 weeks | AER | 42.01 (25.26, 58.76) | <0.001 | <0.001 | 17 |
| Intervention duration | 8–12 weeks | AER_RES | 25.17 (13.96, 36.39) | <0.001 | <0.001 | 17 |
| Intervention duration | 8–12 weeks | AER_RES_BREATH | 61.06 (45.54, 76.58) | <0.001 | <0.001 | 17 |
| Intervention duration | 8–12 weeks | BREATH_IMT | 21.20 (-28.07, 70.47) | 0.399 | <0.001 | 17 |
| Intervention duration | 8–12 weeks | MIND_BODY | 28.34 (-1.91, 58.60) | 0.066 | <0.001 | 17 |
| Intervention duration | >12 weeks | AER_RES | 65.28 (39.75, 90.81) | <0.001 | <0.001 | 5 |
| Intervention duration | >12 weeks | AER_RES_BREATH | 46.76 (38.48, 55.04) | <0.001 | <0.001 | 5 |
| Intervention duration | >12 weeks | BREATH_IMT | 55.03 (8.21, 101.84) | 0.021 | <0.001 | 5 |
| Disease type | IPF | AER | 19.53 (5.27, 33.79) | 0.007 | <0.001 | 12 |
| Disease type | IPF | AER_RES | 24.25 (6.04, 42.46) | 0.009 | <0.001 | 12 |
| Disease type | IPF | AER_RES_BREATH | 74.32 (38.18, 110.46) | <0.001 | <0.001 | 12 |
| Disease type | IPF | BREATH_IMT | 38.98 (5.04, 72.91) | 0.024 | <0.001 | 12 |
| Disease type | IPF | MIND_BODY | 42.93 (26.09, 59.77) | <0.001 | <0.001 | 12 |
| Disease type | Other or mixed ILD | AER | 38.44 (11.03, 65.84) | 0.006 | 14.82 | 14 |
| Disease type | Other or mixed ILD | AER_RES | 34.73 (15.48, 53.98) | <0.001 | 14.82 | 14 |
| Disease type | Other or mixed ILD | AER_RES_BREATH | 55.59 (36.82, 74.36) | <0.001 | 14.82 | 14 |
| Disease type | Other or mixed ILD | BREATH_IMT | 61.32 (16.61, 106.02) | 0.007 | 14.82 | 14 |
| Disease type | Other or mixed ILD | MIND_BODY | 25.80 (-62.60, 114.20) | 0.567 | 14.82 | 14 |
| Supervision level | Higher supervision | AER | 30.12 (9.40, 50.84) | 0.004 | 14.76 | 16 |
| Supervision level | Higher supervision | AER_RES | 34.65 (16.02, 53.28) | <0.001 | 14.76 | 16 |
| Supervision level | Higher supervision | AER_RES_BREATH | 59.76 (41.59, 77.94) | <0.001 | 14.76 | 16 |
| Supervision level | Higher supervision | BREATH_IMT | 70.05 (4.83, 135.27) | 0.035 | 14.76 | 16 |
| Supervision level | Higher supervision | MIND_BODY | 49.63 (18.53, 80.73) | 0.002 | 14.76 | 16 |
| Supervision level | Lower or remote supervision | AER | 38.00 (-47.18, 123.18) | 0.382 | <0.001 | 10 |
| Supervision level | Lower or remote supervision | AER_RES | 25.08 (6.45, 43.70) | 0.008 | <0.001 | 10 |
| Supervision level | Lower or remote supervision | AER_RES_BREATH | 42.80 (-5.03, 90.63) | 0.079 | <0.001 | 10 |
| Supervision level | Lower or remote supervision | BREATH_IMT | 43.11 (14.38, 71.85) | 0.003 | <0.001 | 10 |
| Supervision level | Lower or remote supervision | MIND_BODY | 28.73 (-3.74, 61.19) | 0.083 | <0.001 | 10 |

### FVC% predicted

| **Factor** | **Level** | **Treatment vs CON** | **MD (95% CI)** | **P** | **Tau** | **Studies in subgroup network** |
| --- | --- | --- | --- | --- | --- | --- |
| Intervention setting | Centre/hospital-based | AER | -0.40 (-5.08, 4.28) | 0.867 | 1.19 | 7 |
| Intervention setting | Centre/hospital-based | AER_RES | 2.41 (-2.03, 6.85) | 0.287 | 1.19 | 7 |
| Intervention setting | Centre/hospital-based | AER_RES_BREATH | 2.39 (-1.02, 5.80) | 0.170 | 1.19 | 7 |
| Intervention setting | Centre/hospital-based | MIND_BODY | 1.50 (-22.79, 25.79) | 0.904 | 1.19 | 7 |
| Intervention setting | Home-based/telerehabilitation | AER_RES_BREATH | 5.60 (-0.39, 11.59) | 0.067 | 1.47 | 4 |
| Intervention setting | Home-based/telerehabilitation | BREATH_IMT | -1.00 (-13.07, 11.07) | 0.871 | 1.47 | 4 |
| Intervention setting | Home-based/telerehabilitation | MIND_BODY | -0.84 (-7.76, 6.07) | 0.811 | 1.47 | 4 |
| Intervention duration | 8–12 weeks | AER | -0.40 (-5.61, 4.81) | 0.880 | 1.67 | 9 |
| Intervention duration | 8–12 weeks | AER_RES | 2.97 (-1.54, 7.49) | 0.197 | 1.67 | 9 |
| Intervention duration | 8–12 weeks | AER_RES_BREATH | 3.07 (-0.08, 6.22) | 0.056 | 1.67 | 9 |
| Intervention duration | 8–12 weeks | MIND_BODY | -0.67 (-6.84, 5.51) | 0.832 | 1.67 | 9 |
| Disease type | IPF | AER_RES_BREATH | 6.00 (-0.28, 12.28) | 0.061 | 1.47 | 3 |
| Disease type | IPF | MIND_BODY | -0.84 (-7.76, 6.07) | 0.811 | 1.47 | 3 |
| Disease type | Other or mixed ILD | AER | -0.40 (-4.97, 4.17) | 0.864 | 1.07 | 8 |
| Disease type | Other or mixed ILD | AER_RES | 2.32 (-1.87, 6.52) | 0.278 | 1.07 | 8 |
| Disease type | Other or mixed ILD | AER_RES_BREATH | 2.31 (-0.93, 5.56) | 0.162 | 1.07 | 8 |
| Disease type | Other or mixed ILD | BREATH_IMT | -1.00 (-12.91, 10.91) | 0.869 | 1.07 | 8 |
| Disease type | Other or mixed ILD | MIND_BODY | 1.50 (-22.76, 25.76) | 0.904 | 1.07 | 8 |
| Supervision level | Higher supervision | AER | -0.40 (-5.08, 4.28) | 0.867 | 1.19 | 7 |
| Supervision level | Higher supervision | AER_RES | 2.41 (-2.03, 6.85) | 0.287 | 1.19 | 7 |
| Supervision level | Higher supervision | AER_RES_BREATH | 2.39 (-1.02, 5.80) | 0.170 | 1.19 | 7 |
| Supervision level | Higher supervision | MIND_BODY | 1.50 (-22.79, 25.79) | 0.904 | 1.19 | 7 |
| Supervision level | Lower or remote supervision | AER_RES_BREATH | 5.60 (-0.39, 11.59) | 0.067 | 1.47 | 4 |
| Supervision level | Lower or remote supervision | BREATH_IMT | -1.00 (-13.07, 11.07) | 0.871 | 1.47 | 4 |
| Supervision level | Lower or remote supervision | MIND_BODY | -0.84 (-7.76, 6.07) | 0.811 | 1.47 | 4 |

### SGRQ Total

| **Factor** | **Level** | **Treatment vs CON** | **MD (95% CI)** | **P** | **Tau** | **Studies in subgroup network** |
| --- | --- | --- | --- | --- | --- | --- |
| Intervention setting | Centre/hospital-based | AER | 13.38 (6.43, 20.32) | <0.001 | <0.001 | 6 |
| Intervention setting | Centre/hospital-based | AER_RES | 9.28 (4.75, 13.81) | <0.001 | <0.001 | 6 |
| Intervention setting | Centre/hospital-based | AER_RES_BREATH | 10.35 (7.03, 13.67) | <0.001 | <0.001 | 6 |
| Intervention setting | Home-based/telerehabilitation | AER | -2.00 (-10.81, 6.81) | 0.656 | <0.001 | 5 |
| Intervention setting | Home-based/telerehabilitation | AER_RES_BREATH | 16.40 (7.23, 25.57) | <0.001 | <0.001 | 5 |
| Intervention setting | Home-based/telerehabilitation | BREATH_IMT | 7.61 (2.69, 12.53) | 0.002 | <0.001 | 5 |
| Intervention setting | Home-based/telerehabilitation | MIND_BODY | 15.20 (2.66, 27.74) | 0.017 | <0.001 | 5 |
| Intervention duration | 8–12 weeks | AER | 6.37 (-2.76, 15.49) | 0.171 | 5.18 | 8 |
| Intervention duration | 8–12 weeks | AER_RES | 7.70 (-3.19, 18.59) | 0.166 | 5.18 | 8 |
| Intervention duration | 8–12 weeks | AER_RES_BREATH | 11.17 (3.87, 18.47) | 0.003 | 5.18 | 8 |
| Intervention duration | 8–12 weeks | MIND_BODY | 15.20 (-0.93, 31.33) | 0.065 | 5.18 | 8 |
| Disease type | Other or mixed ILD | AER | 13.37 (6.43, 20.32) | <0.001 | <0.001 | 6 |
| Disease type | Other or mixed ILD | AER_RES | 12.51 (6.79, 18.22) | <0.001 | <0.001 | 6 |
| Disease type | Other or mixed ILD | AER_RES_BREATH | 14.97 (9.00, 20.94) | <0.001 | <0.001 | 6 |
| Disease type | Other or mixed ILD | BREATH_IMT | 1.60 (-11.89, 15.09) | 0.816 | <0.001 | 6 |
| Supervision level | Higher supervision | AER | 13.38 (6.43, 20.32) | <0.001 | <0.001 | 6 |
| Supervision level | Higher supervision | AER_RES | 9.28 (4.75, 13.81) | <0.001 | <0.001 | 6 |
| Supervision level | Higher supervision | AER_RES_BREATH | 10.35 (7.03, 13.67) | <0.001 | <0.001 | 6 |
| Supervision level | Lower or remote supervision | AER | -2.00 (-10.81, 6.81) | 0.656 | <0.001 | 5 |
| Supervision level | Lower or remote supervision | AER_RES_BREATH | 16.40 (7.23, 25.57) | <0.001 | <0.001 | 5 |
| Supervision level | Lower or remote supervision | BREATH_IMT | 7.61 (2.69, 12.53) | 0.002 | <0.001 | 5 |
| Supervision level | Lower or remote supervision | MIND_BODY | 15.20 (2.66, 27.74) | 0.017 | <0.001 | 5 |

## Table S4C. Overall network meta-regression tests

| **Outcome** | **Moderator** | **Reference category** | **Chi-square (df)** | **P** | **Studies** | **Terms** | **Tau** |
| --- | --- | --- | --- | --- | --- | --- | --- |
| 6MWD | Intervention setting | Centre/hospital-based | 1.56 (5) | 0.906 | 26 | 5 | 12.98 |
| 6MWD | Intervention duration | <8 weeks | 18.76 (7) | 0.009 | 26 | 7 | <0.001 |
| 6MWD | Disease type | IPF | 2.96 (5) | 0.706 | 26 | 5 | 12.24 |
| 6MWD | Supervision level | Higher supervision | 1.58 (5) | 0.903 | 26 | 5 | 13.57 |
| FVC% predicted | Intervention setting | Centre/hospital-based | 0.92 (2) | 0.631 | 11 | 2 | 1.20 |
| FVC% predicted | Intervention duration | <8 weeks | 0.10 (1) | 0.750 | 11 | 1 | 1.67 |
| FVC% predicted | Disease type | IPF | 1.16 (2) | 0.559 | 11 | 2 | 1.09 |
| FVC% predicted | Supervision level | Higher supervision | 0.92 (2) | 0.631 | 11 | 2 | 1.20 |
| SGRQ Total | Intervention setting | Centre/hospital-based | 8.69 (2) | 0.013 | 11 | 2 | <0.001 |
| SGRQ Total | Intervention duration | <8 weeks | 0.84 (2) | 0.657 | 11 | 2 | 5.18 |
| SGRQ Total | Disease type | IPF | 10.54 (4) | 0.032 | 11 | 4 | <0.001 |
| SGRQ Total | Supervision level | Higher supervision | 8.69 (2) | 0.013 | 11 | 2 | <0.001 |

## Table S4D. Treatment-specific network meta-regression coefficients

### 6MWD

| **Factor** | **Moderator contrast** | **Treatment** | **Beta (95% CI)** | **P** | **Tau** |
| --- | --- | --- | --- | --- | --- |
| Intervention setting | Home/remote vs centre/hospital | AER | 7.80 (-83.11, 98.72) | 0.866 | 12.98 |
| Intervention setting | Home/remote vs centre/hospital | AER_RES | 9.49 (-50.44, 69.42) | 0.756 | 12.98 |
| Intervention setting | Home/remote vs centre/hospital | AER_RES_BREATH | -15.80 (-72.54, 40.94) | 0.585 | 12.98 |
| Intervention setting | Home/remote vs centre/hospital | BREATH_IMT | -26.93 (-98.40, 44.55) | 0.460 | 12.98 |
| Intervention setting | Home/remote vs centre/hospital | MIND_BODY | -19.00 (-68.15, 30.14) | 0.448 | 12.98 |
| Intervention duration | 8–12 weeks vs <8 weeks | AER | 22.27 (0.09, 44.45) | 0.049 | <0.001 |
| Intervention duration | 8–12 weeks vs <8 weeks | AER_RES | -40.11 (-67.99, -12.22) | 0.005 | <0.001 |
| Intervention duration | 8–12 weeks vs <8 weeks | AER_RES_BREATH | 4.35 (-66.28, 74.99) | 0.904 | <0.001 |
| Intervention duration | 8–12 weeks vs <8 weeks | BREATH_IMT | -39.98 (-103.24, 23.28) | 0.215 | <0.001 |
| Intervention duration | 8–12 weeks vs <8 weeks | MIND_BODY | -19.68 (-55.78, 16.43) | 0.285 | <0.001 |
| Intervention duration | >12 weeks vs <8 weeks | AER_RES_BREATH | -9.95 (-79.35, 59.46) | 0.779 | <0.001 |
| Intervention duration | >12 weeks vs <8 weeks | BREATH_IMT | -6.15 (-67.52, 55.21) | 0.844 | <0.001 |
| Disease type | Other/mixed ILD vs IPF | AER | 19.97 (-15.96, 55.91) | 0.276 | 12.24 |
| Disease type | Other/mixed ILD vs IPF | AER_RES | 8.77 (-22.57, 40.11) | 0.583 | 12.24 |
| Disease type | Other/mixed ILD vs IPF | AER_RES_BREATH | -18.34 (-62.29, 25.62) | 0.414 | 12.24 |
| Disease type | Other/mixed ILD vs IPF | BREATH_IMT | 22.47 (-35.01, 79.96) | 0.444 | 12.24 |
| Disease type | Other/mixed ILD vs IPF | MIND_BODY | -15.96 (-106.12, 74.21) | 0.729 | 12.24 |
| Supervision level | Lower/remote vs higher supervision | AER | 7.82 (-83.54, 99.19) | 0.867 | 13.57 |
| Supervision level | Lower/remote vs higher supervision | AER_RES | -6.96 (-40.79, 26.88) | 0.687 | 13.57 |
| Supervision level | Lower/remote vs higher supervision | AER_RES_BREATH | -16.60 (-74.02, 40.82) | 0.571 | 13.57 |
| Supervision level | Lower/remote vs higher supervision | BREATH_IMT | -26.93 (-98.96, 45.11) | 0.464 | 13.57 |
| Supervision level | Lower/remote vs higher supervision | MIND_BODY | -18.74 (-68.78, 31.30) | 0.463 | 13.57 |

### FVC% predicted

| **Factor** | **Moderator contrast** | **Treatment** | **Beta (95% CI)** | **P** | **Tau** |
| --- | --- | --- | --- | --- | --- |
| Intervention setting | Home/remote vs centre/hospital | AER_RES_BREATH | 3.21 (-3.46, 9.88) | 0.346 | 1.20 |
| Intervention setting | Home/remote vs centre/hospital | MIND_BODY | -2.38 (-27.43, 22.67) | 0.852 | 1.20 |
| Intervention duration | 8–12 weeks vs <8 weeks | AER_RES | -2.03 (-14.48, 10.43) | 0.750 | 1.67 |
| Disease type | Other/mixed ILD vs IPF | AER_RES_BREATH | -3.68 (-10.46, 3.11) | 0.288 | 1.09 |
| Disease type | Other/mixed ILD vs IPF | MIND_BODY | 2.39 (-22.63, 27.42) | 0.851 | 1.09 |
| Supervision level | Lower/remote vs higher supervision | AER_RES_BREATH | 3.21 (-3.46, 9.88) | 0.346 | 1.20 |
| Supervision level | Lower/remote vs higher supervision | MIND_BODY | -2.38 (-27.43, 22.67) | 0.852 | 1.20 |

### SGRQ Total

| **Factor** | **Moderator contrast** | **Treatment** | **Beta (95% CI)** | **P** | **Tau** |
| --- | --- | --- | --- | --- | --- |
| Intervention setting | Home/remote vs centre/hospital | AER | -15.37 (-26.59, -4.16) | 0.007 | <0.001 |
| Intervention setting | Home/remote vs centre/hospital | AER_RES_BREATH | 6.05 (-3.71, 15.80) | 0.224 | <0.001 |
| Intervention duration | 8–12 weeks vs <8 weeks | AER_RES | -5.30 (-21.79, 11.18) | 0.529 | 5.18 |
| Intervention duration | >12 weeks vs <8 weeks | BREATH_IMT | 6.93 (-13.46, 27.32) | 0.505 | 5.18 |
| Disease type | Other/mixed ILD vs IPF | AER | 15.37 (4.16, 26.59) | 0.007 | <0.001 |
| Disease type | Other/mixed ILD vs IPF | AER_RES | 6.51 (-8.93, 21.94) | 0.409 | <0.001 |
| Disease type | Other/mixed ILD vs IPF | AER_RES_BREATH | 5.27 (-1.76, 12.29) | 0.142 | <0.001 |
| Disease type | Other/mixed ILD vs IPF | BREATH_IMT | -6.93 (-21.42, 7.56) | 0.349 | <0.001 |
| Supervision level | Lower/remote vs higher supervision | AER | -15.37 (-26.59, -4.16) | 0.007 | <0.001 |
| Supervision level | Lower/remote vs higher supervision | AER_RES_BREATH | 6.05 (-3.71, 15.80) | 0.224 | <0.001 |

*All subgroup and network meta-regression analyses were exploratory. Study-level covariates overlapped across several contrasts, sparse strata could yield unstable coefficients, and nominal P values were not adjusted for multiple testing. These estimates should therefore be interpreted as hypothesis-generating rather than as confirmatory evidence of effect modification.*

# Supplementary Table S5. Heterogeneity, Global Inconsistency, and Node-Splitting Analyses

| **Outcome** | **Studies** | **Arms** | **Participants** | **Nodes** | **Tau** | **Tau²** | **Heterogeneity Q (df), P** | **Global inconsistency chi-square (df), P** |
| --- | --- | --- | --- | --- | --- | --- | --- | --- |
| 6MWD | 26 | 53 | 1339 | 6 | 12.02 | 144.55 | 28.07 (23), P = 0.213 | 3.80 (3), P = 0.284 |
| FVC% predicted | 11 | 22 | 446 | 6 | 1.55 | 2.40 | 5.57 (7), P = 0.591 | 0.06 (1), P = 0.815 |
| SGRQ Total | 11 | 22 | 431 | 6 | 4.22 | 17.79 | 11.45 (7), P = 0.120 | 0.05 (1), P = 0.823 |

## Node-splitting results

### 6MWD

| **Comparison** | **Direct effect** | **Indirect effect** | **Difference** | **P for difference** | **Tau** | **Outside evidence** |
| --- | --- | --- | --- | --- | --- | --- |
| AER vs CON | 30.98 | -15.22 | 46.21 | 0.322 | 11.94 | 0 |
| AER_RES vs CON | 35.29 | 13.28 | 22.00 | 0.258 | 11.51 | 1 |
| BREATH_IMT vs CON | Not estimable | Not estimable | Not estimable | Not estimable | Not estimable | Not estimable |
| AER_RES_BREATH vs CON | 52.93 | 74.92 | -21.99 | 0.259 | 11.51 | 1 |
| MIND_BODY vs CON | 40.30 | 84.66 | -44.35 | 0.204 | 11.21 | 0 |
| MIND_BODY vs AER | 28.34 | -10.42 | 38.76 | 0.116 | 10.12 | 1 |
| AER_RES_BREATH vs AER_RES | 39.64 | 17.64 | 22.00 | 0.259 | 11.51 | 1 |

### FVC% predicted

| **Comparison** | **Direct effect** | **Indirect effect** | **Difference** | **P for difference** | **Tau** | **Outside evidence** |
| --- | --- | --- | --- | --- | --- | --- |
| AER vs CON | Not estimable | Not estimable | Not estimable | Not estimable | Not estimable | Not estimable |
| AER_RES vs CON | 5.00 | 2.97 | 2.03 | 0.750 | 1.67 | 1 |
| BREATH_IMT vs CON | Not estimable | Not estimable | Not estimable | Not estimable | Not estimable | Not estimable |
| AER_RES_BREATH vs CON | 3.09 | 4.51 | -1.42 | 0.815 | 1.68 | 1 |
| MIND_BODY vs CON | Not estimable | Not estimable | Not estimable | Not estimable | Not estimable | Not estimable |
| AER_RES_BREATH vs AER_RES | 0.08 | -1.68 | 1.76 | 0.780 | 1.68 | 1 |

### SGRQ Total

| **Comparison** | **Direct effect** | **Indirect effect** | **Difference** | **P for difference** | **Tau** | **Outside evidence** |
| --- | --- | --- | --- | --- | --- | --- |
| AER vs CON | Not estimable | Not estimable | Not estimable | Not estimable | Not estimable | Not estimable |
| AER_RES vs CON | 10.70 | 8.75 | 1.95 | 0.822 | 5.06 | 1 |
| BREATH_IMT vs CON | Not estimable | Not estimable | Not estimable | Not estimable | Not estimable | Not estimable |
| AER_RES_BREATH vs CON | 11.50 | 13.43 | -1.94 | 0.823 | 5.07 | 1 |
| MIND_BODY vs CON | Not estimable | Not estimable | Not estimable | Not estimable | Not estimable | Not estimable |
| AER_RES_BREATH vs AER_RES | 2.74 | 0.80 | 1.94 | 0.822 | 5.07 | 1 |

*Failure to detect inconsistency should not be interpreted as proof of consistency, particularly in sparse networks with few closed loops. “Not estimable” indicates that direct and indirect evidence could not be separated for that comparison. The column “Outside evidence” reproduces the software output and is provided for auditability; inconsistency judgments should be based on the direct–indirect difference and its P value.*

# Supplementary Table S6. Treatment-Ranking Results for the Three Primary Outcomes

| **Outcome** | **SUCRA rank** | **Treatment** | **SUCRA (%)** | **Mean rank** | **Probability of being best (%)** |
| --- | --- | --- | --- | --- | --- |
| 6MWD | 1 | AER_RES_BREATH | 90.57 | 1.47 | 61.49 |
| 6MWD | 2 | BREATH_IMT | 72.28 | 2.39 | 27.15 |
| 6MWD | 3 | MIND_BODY | 64.27 | 2.79 | 11.05 |
| 6MWD | 4 | AER_RES | 39.04 | 4.05 | 0.06 |
| 6MWD | 5 | AER | 33.82 | 4.31 | 0.25 |
| 6MWD | 6 | CON | 0.03 | 6.00 | 0.00 |
| FVC% predicted | 1 | AER_RES_BREATH | 79.08 | 2.05 | 28.61 |
| FVC% predicted | 2 | AER_RES | 77.57 | 2.12 | 35.45 |
| FVC% predicted | 3 | BREATH_IMT | 37.94 | 4.10 | 21.07 |
| FVC% predicted | 4 | CON | 36.28 | 4.19 | 0.33 |
| FVC% predicted | 5 | AER | 35.50 | 4.22 | 6.86 |
| FVC% predicted | 6 | MIND_BODY | 33.62 | 4.32 | 7.68 |
| SGRQ Total | 1 | MIND_BODY | 80.75 | 1.96 | 59.69 |
| SGRQ Total | 2 | AER_RES_BREATH | 74.22 | 2.29 | 21.92 |
| SGRQ Total | 3 | AER_RES | 61.47 | 2.93 | 10.54 |
| SGRQ Total | 4 | BREATH_IMT | 40.62 | 3.97 | 4.08 |
| SGRQ Total | 5 | AER | 39.94 | 4.00 | 3.77 |
| SGRQ Total | 6 | CON | 2.99 | 5.85 | 0.00 |

*Ranking probabilities describe uncertainty in relative ranks and should be interpreted together with treatment effects, confidence intervals, the amount of direct evidence, and CINeMA confidence ratings. A high SUCRA value alone does not establish clinical superiority.*

Abbreviations: 6MWD, six-minute walk distance; AER, aerobic training; AER_RES, combined aerobic and resistance training; AER_RES_BREATH, combined aerobic, resistance, and breathing or inspiratory-muscle training; BREATH_IMT, breathing or inspiratory-muscle training; CI, confidence interval; CINeMA, Confidence in Network Meta-Analysis; CON, control; FVC, forced vital capacity; IMT, inspiratory-muscle training; MD, mean difference; MIND_BODY, mind–body exercise; NMA, network meta-analysis; SGRQ, St George’s Respiratory Questionnaire; SMD, standardized mean difference; SUCRA, surface under the cumulative ranking curve.

# Supplementary Figure S1. Random-Effects Network Meta-Analysis Forest Plots

Panels A-C show network estimates versus CON for 6MWD, FVC% predicted, and SGRQ Total, respectively. Positive estimates indicate benefit; SGRQ was reverse-coded before analysis.

# Supplementary Figure S2. Prediction-Interval Plots

Panels A-C show 95% confidence intervals and 95% prediction intervals for 6MWD, FVC% predicted, and SGRQ Total, respectively.

# Supplementary Figure S3. Leave-One-Out Sensitivity Analyses Across All Intervention Nodes

Panels A-C summarize leave-one-out estimates for all intervention nodes versus CON for the three outcomes.

# Supplementary Figure S4. Detailed Leave-One-Out Analysis for 6MWD: AER_RES_BREATH versus CON

Each row omits one trial. The vertical reference corresponds to the full-network estimate.

# Supplementary Figure S5. Detailed Leave-One-Out Analysis for FVC% Predicted: AER_RES_BREATH versus CON

Each row omits one trial. Four of 11 leave-one-out confidence intervals crossed the null value, indicating limited robustness of the random-effects primary estimate.

# Supplementary Figure S6. Detailed Leave-One-Out Analysis for SGRQ Total: AER_RES_BREATH versus CON

Each row omits one trial. Positive values indicate improvement because SGRQ changes were reverse-coded.

# Supplementary Figure S7. Primary Analyses versus Analyses Restricted to Directly Reported Change Data

Panels A-C compare primary estimates with analyses retaining only trial-outcome datasets in which all arms directly reported change means and change SDs. The strict FVC analysis used a fixed-effect consistency model.

# Supplementary Figure S8. CINeMA Assessment for FVC% Predicted

Confidence was evaluated across within-study bias, reporting bias, indirectness, imprecision, heterogeneity, and incoherence. Overall confidence for AER_RES and AER_RES_BREATH versus CON was low; the remaining active interventions versus CON were rated very low.

# Supplementary Figure S9. CINeMA Assessment for SGRQ Total

Overall confidence for AER_RES_BREATH versus CON was moderate; AER_RES, BREATH_IMT, and MIND_BODY were low, and AER was very low.
