# Supplementary material for: Comparative effects of different exercise training modalities on exercise capacity, pulmonary function, and health-related quality of life in patients with interstitial lung disease: a systematic review and network meta-analysis
Source: Front Med (Lausanne). 2026 Oct 8;13:1953668. doi: 10.3389/fmed.2026.1953668 (PMC13687353; doi:10.3389/fmed.2026.1953668)
Supplement: Supplementary file 2 [file Supplementaryfile2.docx]

|  | **Identification of studies via databases** |  |
| --- | --- | --- |

| Identification |  | **Records identified from:** • Embase (n = 3,138) • Scopus (n = 1,690) • Cochrane Library (n = 1,536) • PubMed (n = 737) • Web of Science (n = 398) • PEDro (n = 180) Total (n = 7,679) | → | **Records removed before screening:** • Duplicate records removed (n = 2,276) |
| --- | --- | --- | --- | --- |
|  |  | ↓ |  |  |
| Screening |  | **Records screened by title and abstract** (n = 5,403) | → | **Records excluded after title and abstract screening** (n = 5,353) |
|  |  | ↓ |  |  |
|  |  | **Reports sought for retrieval** (n = 50) | → | **Reports not retrieved** (n = 3) |
|  |  | ↓ |  |  |
|  |  | **Reports assessed for eligibility** (n = 47) | → | **Reports excluded (n = 21):** • Non-randomized or randomization status unconfirmed (n = 3) • Same prespecified intervention node only (n = 4) • Secondary, follow-up, or companion report of the same trial (n = 6) • Insufficient prespecified outcome data (n = 8) |
|  |  | ↓ |  |  |
| Included |  | **Randomized trials included** (26 primary reports; 26 independent trials) One primary report retained per trial |  |  |
